# Wise reasoning, intergroup positivity, and attitude polarization across contexts

**DOI:** 10.1038/s41467-021-23432-1

**Published:** 2021-06-03

**Authors:** Justin P. Brienza, Franki Y. H. Kung, Melody M. Chao

**Affiliations:** 1 University of Queensland Business School, St. Lucia, QLD Australia; 2 Department of Psychological Sciences, Purdue University, West Lafayette, IN USA; 3 Department of Management, Hong Kong University of Science and Technology, Clear Water Bay, Kowloon Hong Kong

**Keywords:** Human behaviour, Decision making, Society

## Abstract

We hypothesized that a wisdom-based reasoning process comprised of epistemic humility, accounting for context, and integrating different perspectives and interests, would be helpful in overcoming intergroup bias and attitude polarization in societal conflicts. Here we test the hypothesis using both the Situated Wise Reasoning Scale and experimental induction. In each study, we recruited participants who self-identified as members of a group implicated in an ongoing intergroup situation. In five correlational studies (Studies 1-5) we examined the relations between measured wise reasoning and intergroup positivity and attitude polarization. In two experiments, we tested the effects of a brief online wise-reasoning thought exercise on intergroup positivity and polarization (Studies 6-7), and charitable behaviors to an outgroup (Study 6). We found that wise reasoning relates to more positivity toward outgroups and less attitude polarization across different groups and conflicts. The results have implications for theory and may also have implications for future research on interventions to improve intergroup relations.

## Abstract (web summary)

Here, the authors show that an integrative thinking process linked philosophically to wisdom may reduce group polarization. Specifically, wise reasoning improves intergroup attitudes and behavior even at time of heightened societal conflicts.

## Introduction

Society has entered a polarized decade, laden with and threatened by social division^1^. People are exposed daily with news about intergroup conflicts. Witnessing escalating intergroup bias and conflicts—and perhaps experiencing them first-hand—makes it difficult to hold positive and balanced intergroup attitudes^2–5^. Identifying psychological factors that can ameliorate intergroup bias amid heightened conflict has therefore become a critical research imperative^2^. Integrating rich philosophical traditions with recent empirical study on psychological wisdom^6–11^, we propose that the use of wisdom-related thinking processes (i.e., wise reasoning) relates to more outgroup positivity and less ingroup-vs.-outgroup attitude polarization in intergroup conflicts.

Philosophers have for millennia discussed wisdom as an important attribute that could unite people and help them to overcome bias and attain balance and cooperation^12–16^. Across different cultures and schools of thought, wisdom scholars sought to optimize the way people manage difficult social challenges (e.g., Aristotle’s phronesis or practical wisdom) and to understand how to live free from self-centered, extreme reactions in exchange for balance and charitability^9,10,17,18^. Wisdom has been characterized in related ways throughout the ages (e.g., Socrates’ recognition of one’s own ignorance;^19^ contemplation of change in the *I Ching*^12^). Drawing on these traditions from across different cultures, contemporary scholars generally agree that wisdom rests fundamentally on an integrative reasoning process that synthesizes (i) epistemic humility (recognizing and accommodating for uncertainty and the limits of one’s own knowledge), (ii) contextualism (looking at the bigger picture and change in situations), (iii) perspectivism (trying to understand situations from multiple angles), and (iv) dialecticism (e.g., integrating different interests)^9,15,20–24^.

Wise reasoning theoretically enables people to solve challenges pragmatically because it goes beyond self-centered cognitions, adding deeper reflection on complex circumstances and a bigger picture orientation to provide a better understanding of situations^25^. In so doing, it may temper biased responses to social challenges (e.g., stereotypes; gut reactions against outgroups) with less skewed and more nuanced information (e.g., individuating information; shared humanity between groups). As such, in intergroup contexts wise reasoning may afford more positivity (or less extreme negativity) toward outgroups and less attitude polarization. Indeed, recent studies have shown that wise reasoning has an adaptive influence on important outcomes such as prosocial behavior (e.g., cooperation in public goods games)^26^, cognitive biases (e.g., bias blindspot), and balanced attributions toward others in personal conflicts^20^. Further wise reasoning is strengthened via moral and interdependent motivations^27^.

Although explicit use of the term “wise reasoning” in reference to the integration of wisdom-based reasoning processes is relatively recent^28^, research has suggested its importance indirectly via reference to some of its individual dimensions. For instance, inducing people to recognize, reappraise, and challenge their own habitual thoughts and emotions, whether through lab experiments or through field experiences^22,29–33^, is a process of epistemic humility that can reduce intergroup biases. Similarly, the conflict management literature has speculated on the importance of “going to the balcony” (observer’s viewpoint^34^), a process of contextualism, and “stepping to the other side” (perspective taking^35^), a process of perspectivism, for resolving conflicts. However, the positive effects of these processes individually may not generalize broadly when taken apart from the integrated wise reasoning process. Perspective taking alone, for example, can vary in effectiveness depending on region or culture^36^ and it can improve dominant groups’ attitudes toward disadvantaged groups but not vice-versa^37^. The process itself can be egocentrically biased^38–40^ or used for intentionally selfish or malicious aims^35,41^. In contrast, because wise reasoning is an integrative, self-decentering process^25^, we speculated that it would show more consistency and broad generalizability, showing an inverse relationship with intergroup bias if and when engaged, despite differences in culture, group membership, or status.

Verifying these assumptions requires putting wise reasoning to test across different intergroup conflict situations involving different groups. Until recently, however, problems with measuring wisdom has made this difficult: Although observer-scored measures^42,43^ offer independent and criterion-based measurement, they discourage large-scale study because they are extremely resource- and labor-intensive to generate. Further, even though they may provide good assessments of behavior, observer reports can be limited in providing assessment of psychological processes^44^ and can be biased by the observer^45^. Conversely, traditional self-report measures afford large-scale study but they ignore context and invite biased responding^46,47^. However, recent developments in efficient situation-specific measurement^23,48–51^ provide methods for conducting valid, context-sensitive large-scale studies examining the philosophical claims about the role of wise reasoning in social challenges. The new hybrid methods assess people in the midst or in recall of specific states, querying them about their momentary reflections on the matter^20^. They are therefore ideal for situated measurement.

In this work, we establish a relationship between wise reasoning and intergroup bias across different polarized societal conflicts. We hypothesize that wise reasoning will show an inverse relationship with intergroup bias, as indicated by (i) more positivity and charitability toward outgroups, and (ii) less polarized (more balanced) attitudes between ingroup and outgroup. Studies 1–5 establish and replicate a relationship between wise reasoning and intergroup bias, adding ecological validity and generalizability by conducting the studies in different societal contexts with different intergroup dynamics, and by using different research designs (i.e., within-subjects; between-subjects). We conduct two meta-analytic tests estimating the true magnitude of the relationships between wise reasoning and outgroup positivity and attitude polarization. Although previous work has validated wise reasoning as unique from numerous other constructs (e.g., IQ, personality; motivations and values; thinking styles)^20,42,52,53^, Study 5 examines incremental and unique validity of wise reasoning for predicting intergroup bias. Finally, Studies 6 and 7 use experimental wise reasoning induction to (i) provide initial evidence of causality and pave way to examine training and education effectiveness in the future, (ii) examine practical downstream implications, (iii) eliminate a potential confound, and (iv) examine potential mediators of the effect of wise reasoning on decreased polarization.

## Results

### General study procedure

Samples and group categories involved depend on the respective conflict under consideration in each study. Generally, in intergroup research, ingroups are defined as groups of people who share similar social identity, socio-political stance, or values in relation to the conflict, whereas outgroups are defined as groups of people who do not share these attributes^54^. Thus, in these studies group membership was assigned via participants’ self-reported membership in or identification with a group. We chose to study these particular examples of intergroup conflicts because they were prominent in the news between 2014 and 2020 and covered diverse issues in varied regions. In each study, participants reflected on stimulus materials related to the respective self-relevant conflict (e.g., news clippings of the conflicts). They then indicated their attitudes to groups in the conflict as a measure of intergroup bias (warmth and trust ratings or feeling thermometer ratings). Ingroup attitudes were defined as the evaluation toward the group that a participant identified with in the conflict and outgroup attitudes were defined as evaluation toward the group that a participant did not identify with. Attitude polarization was defined as the difference between the two attitudes (either between-group ratings toward a single target group or within-group ratings toward two target groups). All participants responded to the wise reasoning measure (Situated Wise Reasoning Scale (SWIS)) assessing their reflections on the conflict. In Studies 6–7, we used a brief online wise reasoning reflection exercise, testing its causal impact on intergroup bias. Supplementary Table 1 presents sample characteristics of each study. Detailed procedure, analyses, and results (e.g., confidence intervals, data exclusion) are presented in full in the Supplemental Information. In the following, all statistical tests are two-tailed, unless otherwise specified.

### Initial tests

Study 1 (Hong Kong, in 2014) and Study 2 (USA, in 2015) establish the relation between wise reasoning and intergroup bias with a basic between-subjects design. They assessed intergroup bias by comparing positivity ratings toward a single target group, with ingroup and outgroup participants rating the same target on warmth and trust^55^ (*α*s ≥ 0.90). The studies were conducted in different contexts for generalizability. Study 1 compared attitudes toward the protesters of the Umbrella Movement Hong Kong, in 2014, across ingroup (protesters; *n* = 42) and outgroup (non-protesters; *n* = 33). Study 2 focused on attitudes toward police, as a function of participants’ self-reported identification with those who protested following the death of a Black man in police custody in Baltimore, USA, in 2015 (continuous variable; *n* = 337), with relatively strong and weak identification with the protestors serving as a proxy for ingroup and outgroup identity.

**Fig. 1:**
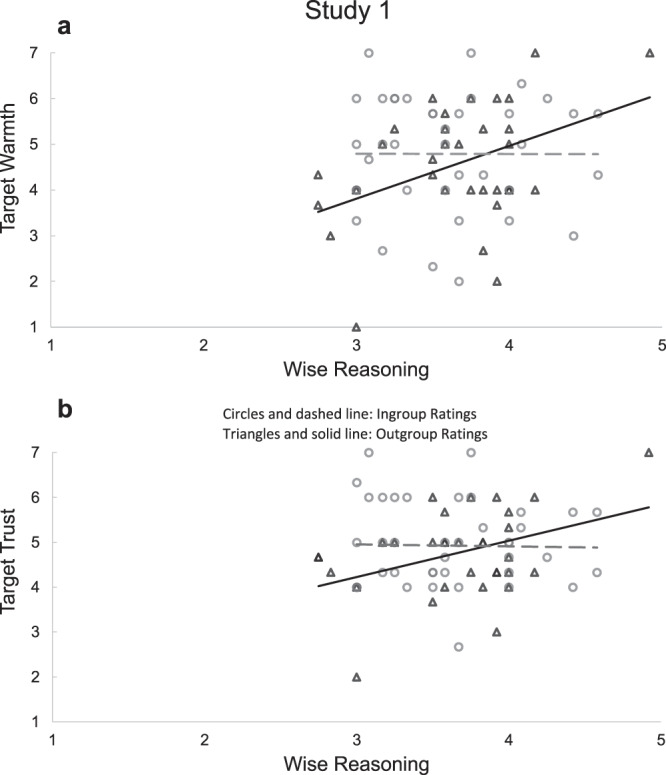
Results from Study 1: positivity toward target group as a function of participants’ self-reported ingroup or outgroup membership and their level of wise reasoning. **a** Warmth ratings. **b** Trust ratings. One outlier was removed for ease of presentation; see Fig. S9 for a Figure including the outlier. *M*_wise reasoning_ = 3.59, SD = 0.56.

**Fig. 2:**
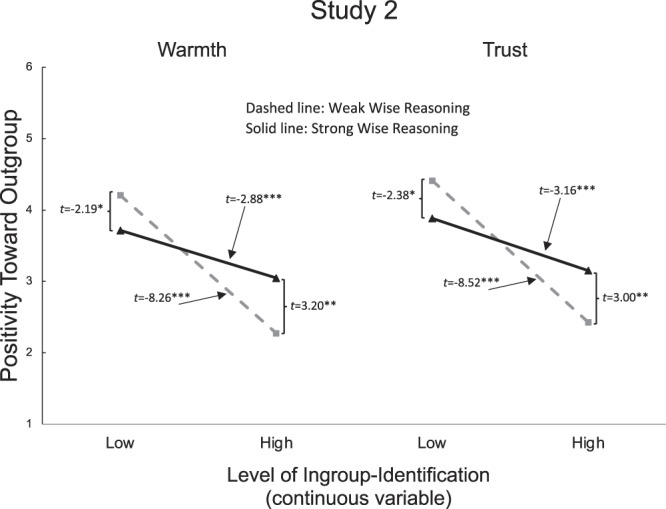
Results from Study 2: positivity toward target group as a function of the strength of participants’ self-reported ingroup-identification (a continuous variable) and their level of wise reasoning. Comparisons of strong and weak wise reasoning as well as high and low identification are presented at the level of ±1 standard deviation from the mean. *M*_wise reasoning_ = 3.38, SD = 0.79; *M*_identification_ = 3.45, SD = 1.98.

For Studies 1–2, we submitted warmth and trust ratings (toward protestors in Study 1 and toward police in Study 2) to separate multiple regression models with Group Membership, wise reasoning (measured with the SWIS scale), and a Group Membership × wise reasoning interaction term as predictors. Both studies showed significant interactions—Study 1: *B*_warmth_ = 1.25, SE = 0.53, *t*(70) = 2.36, *p* = 0.021, *η*^2^_*p*_ = 0.07, 95% CI [0.19, 2.31], *B*_trust_ = 1.16, SE = 0.40, *t*(70) = 2.88, *p* = 0.005, *η*^2^_*p*_ = 0.11, 95% CI [0.36, 1.96], and Study 2: *B*_warmth_ = 0.20, SE = 0.05, *t*(333) = 4.08, *p* < 0.001, *η*^2^_*p*_ = 0.05, 95% CI [0.10, 0.30], *B*_trust_ = 0.20, SE = 0.05, *t*(333) = 4.06, *p* < 0.001, *η*^2^_*p*_ = 0.05, 95% CI [0.10, 0.30]—indicating that wise reasoning moderated group effects on positivity (Figs. 1 and 2). In Study 1, non-protester participants’ wise reasoning related to more positivity toward the outgroup: *t*(31)_warmth_ = 3.83, *p* = 0.001, 95% CI [0.58, 1.92]; *t*(31)_trust_ = 4.52, *p* < 0.001, 95% CI [0.61, 1.62]. To examine between-group polarization, we compared group differences in ratings—between protester and non-protester participants—at weak (−1SD) and strong (+1SD) levels of wise reasoning. Among those with weaker wise reasoning (−1SD), the outgroup (vs. ingroup) participants showed less positivity toward protesters, *t*(70)_warmth_ = −2.43, *p* = 0.018, 95% CI [−1.83, −0.18]; *t*(70)_trust_ = −2.91, *p* = 0.005, 95% CI [−1.53, −0.29], but among those who had stronger wise reasoning (+1SD) there was no group difference (*p* = 0.231). Replicating these effects on a larger sample in Study 2, wise reasoning of participants who strongly identified with the protesters related to more positivity toward the outgroup, *t*(333)_warmth_ = 3.20, *p* = 0.002, 95% CI [0.19, 0.79]; *t*(333)_trust_ = 3.00, *p* = 0.003, 95% CI [0.16, 0.75]. Moreover, a group difference in positivity was found among those with weaker wise reasoning, *t*(333)_warmth_ = −8.26, *p* < 0.001, 95% CI [−0.60, −0.37]; *t*(333)_trust_ = −8.52, *p* < 0.001, 95% CI [−0.62, −0.39], yet the difference was smaller for those with stronger wise reasoning, *t*(333)_warmth_ = −2.88, *p* = 0.004, 95% CI [−0.28, −0.05]; *t*(333)_trust_ = −3.16, *p* = 0.002, 95% CI [−0.30, −0.07]. Studies 1-2 thus supported the hypotheses that wise reasoning relates to (i) outgroup positivity, and (ii) less extreme intergroup attitude polarization.

### Replication and extension

Study 3 (Hong Kong, 2015) and Study 4 (USA, 2015) situated the investigation in new conflict conditions and replicated the findings in several different ways: investigating intergroup bias within individuals (Study 3) and within individuals across groups (mixed design; Study 4). They also used a different measure of intergroup attitudes—feeling thermometer ratings (0 = extremely cold/unfavorable, to 100 = extremely warm/favorable)^56^, rating both outgroup and ingroup target groups. Further, Study 4 examined whether the effect of wise reasoning would be mirrored between two groups that were in a conflict. This is important because previously examined psychological variables have not shown consistent associations with intergroup bias among both majority and minority status groups in conflict.

Study 3 examined Hong Kong Chinese participants’ (*n* = 298) attitudes toward ingroup (Hong Kong Chinese individuals) and outgroup (Mainland Chinese individuals) in the context of demonstrations that began in Hong Kong in 2015 in response to the increasing numbers of Mainland Chinese visitors. Study 4 was conducted in the two days following the United States Supreme Court ruling in favor of same-sex marriage in 2015, which was associated with conflicts between Christian and Conservative groups and LGBTQ and Liberal groups across the USA. In this study, we examined attitudes toward “Christians” and “Gays”. The term “Gays” was used based on precedent from the literature at the time when the study was conducted^57^. We recruited participants who self-identified as Christian or Conservative (*n* = 144) and LGBTQ or Liberal (*n* = 99).

**Fig. 3:**
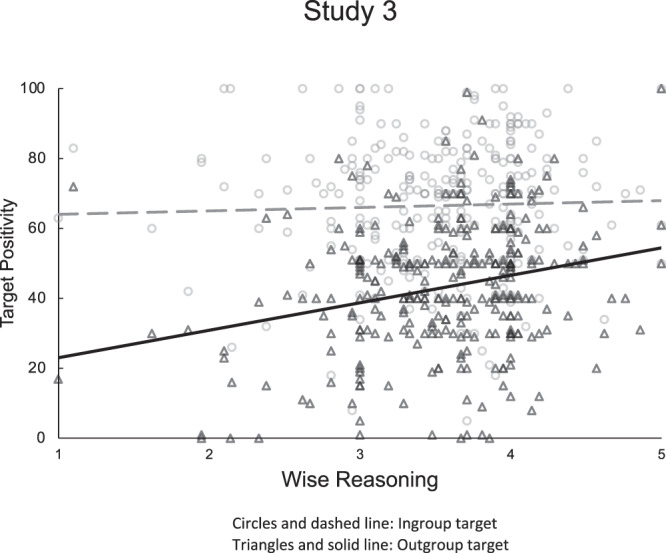
Results from Study 3: within-subject positivity toward ingroup and outgroup targets in feeling thermometer ratings as a function of group members’ level of wise reasoning. *M*_wise reasoning_ = 3.56, *SD* = 0.59.

We submitted ingroup and outgroup feeling thermometer ratings to mixed-model regressions with wise reasoning (measured with the SWIS scale) as the moderator. In Study 4, we also entered Group Membership (Christian/Conservative vs. LGBTQ/Liberal participants) as a predictor. Both studies showed significant interactions—Study 3: a 2-way Target Group × wise reasoning: *F*(1, 296) = 9.55, *p* = 0.002, *η*^2^_*p*_ = 0.03, and Study 4: a 3-way Group Membership × Target Group × wise reasoning: *F*(1, 238) = 5.49, *p* = 0.020, *η*^2^_*p*_ = 0.02, indicating that wise reasoning moderated group effects on positivity. In Study 3, wise reasoning predicted stronger positivity toward the outgroup, *B* = 7.85, SE = 1.77, *t*(296) = 4.44, *p* < 0.001, *η*^2^_*p*_ = 0.06, 95% CI [4.36, 11.33], but not the ingroup (*p* > 0.250; Fig. 3). Participants with weaker wise reasoning showed strong attitude polarization, *t*(296) = 14.75, *p* < 0.001, *η*^2^_*p*_ = 0.42; this effect was weaker in participants with stronger wise reasoning, *t*(296) = 10.38, *p* < 0.001, *η*^2^_*p*_ = 0.27. Study 4 replicated and extended these effects across both groups of participants. Among Christian/conservative participants, wise reasoning predicted more positivity toward the outgroup, *B* = 7.61, SE = 3.62, *t*(141) *=* 2.10, *p* = 0.037, *η*^2^_*p*_ = 0.03, 95% CI [0.45, 14.77], but not the ingroup (*p* > 0.250). Likewise, among LGBTQ/liberal participants, wise reasoning predicted more positivity toward the outgroup, *B* = 6.44, *SE* = 2.66, *t*(97) *=* 2.05, *p* = 0.009, *η*^2^_*p*_ = 0.07, 95% CI [1.64, 11.24], but not the ingroup (*p* > 0.250). Christian/conservative participants with weaker wise reasoning showed attitude polarization, *t*(142) = −2.72, *p* = 0.007, *η*^2^_*p*_ = 0.05, but those with stronger wise reasoning did not (*p* > 0.250). LGBTQ/liberal participants with weaker wise reasoning showed stronger attitude polarization, *t*(97) *=* *−*14.39, *p* < 0.001, *η*^2^_*p*_ = 0.68, but polarization was weaker among those with stronger wise reasoning, *t*(97) = −10.09, *p* < 0.001, *η*^2^_*p*_ = 0.51. These studies replicate and extend the first two studies, showing that wise reasoning relates to (i) outgroup positivity, and (ii) less extreme intergroup attitude polarization within individuals (Studies 3–4) for both majority and minority status groups in the conflict (Study 4).

### Replication on majority and minority groups in conflict

**Fig. 4:**
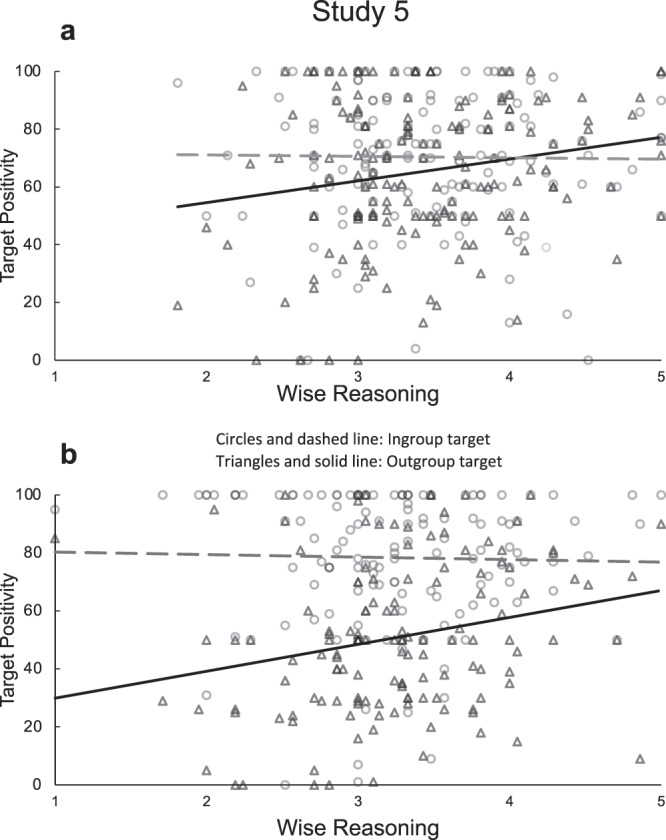
Results from Study 5: within-subject positivity toward ingroup and outgroup targets in feeling thermometer as a function of self-reported group membership and their level of wise reasoning. **a** Christian heterosexual participants. **b** Lesbian, gay, or bisexual participants. *M*_wise reasoning_ = 3.34, SD = 0.65.

Study 5 (Canada, 2015) aims to replicate the findings by comparing intergroup bias between individuals identifying as Christian heterosexuals (*n* = 166) or LGBTQ (*n* = 142; we received responses only from participants who self-identified as lesbian, gay, or bisexual) at a Canadian University. In this study, we examined attitudes toward target groups referred to in the survey as “Christians” and “Homosexuals”. The term “Homosexuals” instead of “Gays” was used in this study because past research suggested that attitudes might differ depending on the terms used to describe sexual minority groups^58,59^. Using different terms across studies allowed us to explore the generalizability of wise reasoning. We submitted ingroup and outgroup feeling thermometer ratings to a mixed-model regression with Group Membership as the predictor and wise reasoning (measured with the SWIS scale) as the moderator. We found a significant 3-way Group Membership × Target Group × wise reasoning interaction, *F*(1, 298) = 8.50, *t*(298) = 2.92, *p* = 0.004, *η*^2^_*p*_ = 0.03 (Fig. 4), indicating that wise reasoning moderated the effects of group membership on intergroup bias for both groups of participants. Among Christian heterosexual participants, wise reasoning predicted more positivity toward the outgroup, *B* = 7.54 SE = 3.03, *t*(161) *=* 2.49, *p* = 0.014, *η*^2^_*p*_ = 0.04, 95% CI [1.57, 13.52], but not the ingroup (*p* > 0.250). Likewise, among lesbian, gay, or bisexual participants, wise reasoning predicted more positivity toward the outgroup, *B* = 9.29, SE = 3.40, *t*(137) *=* 2.73, *p* = 0.007, *η*^2^_*p*_ = 0.05, 95% CI [2.56, 16.01], but not the ingroup (*p* > 0.250). Christian heterosexual participants with weaker wise reasoning showed attitude polarization, *t*(161) = 2.75, *p* = 0.007, *η*^2^_*p*_ = 0.05, but those with stronger wise reasoning did not (*p* > 0.250). Lesbian, gay, or bisexual participants with weaker wise reasoning showed attitude polarization, *t*(137) *=* 7.84, *p* < 0.001, *η*^2^_*p*_ = 0.31, but polarization was weaker among those with stronger wise reasoning, *t*(137) = 3.86, *p* < 0.001, *η*^2^_*p*_ = 0.10. These results replicate Study 4 in a similar conflict context in a different region. They show that wise reasoning moderates ingroup vs. outgroup target positivity for groups of different status in a conflict.

### Incremental and unique predictive validity

In Study 5 we also assessed participants on validated measures of perspective taking and empathic concern^60^, and need for cognitive closure^61^ (all showing high reliability, *α*s > = 0.80), psychological constructs common to intergroup bias research, to explore the validity of wise reasoning. Specifically, we examined (i) incremental validity—whether the association between wise reasoning and less intergroup bias for both groups in a conflict would hold when controlling for other comparison variables, and (ii) unique validity—how the relationship of wise reasoning to intergroup bias compared to that of the comparison variables.

For tests of incremental validity we re-ran the focal analysis, testing whether the observed 3-way Group Membership × Target Group × wise reasoning interaction would hold when controlling for comparison variables (see Supplementary Table 4). We entered each of perspective taking, empathic concern, or need for closure individually (Models 2–4), all together in the same model (Model 5), including each main effect and 3-way interactions (i.e., Group Membership × Target Group × Covariate) entered individually (Models 6–8), and altogether in the same model (Model 9). In each case, the relation between wise reasoning and intergroup bias remained significant.

For tests of unique predictive validity, we unpacked the focal interactions for the three control variables, exploring whether they would predict more positivity to outgroups and less attitude polarization for both groups in a conflict as wise reasoning does. In the three separate tests, identical to our focal wise reasoning test but substituting the comparison variables, we found no comparable 3-way interactions (*p*s > 0.250). Although perspective taking related to less intergroup bias in majority Christian heterosexual participants, specifically relating to positivity to the minority outgroup, *t*(161) = 2.01, *p* = 0.047, 95% CI [0.09, 12.23], but not the ingroup (*p* > 0.250), we did not observe the same effect in minority lesbian, gay, or bisexual participants. For lesbian, gay, or bisexual participants, perspective taking predicted more positivity only to the ingroup targets, *t*(137) = 3.47, *p* = 0.001, 95% CI [4.35, 15.92], suggesting that perspective taking can exacerbate attitude polarization for minority groups, consistent with prior findings^37^. Empathic concern related to positivity toward both ingroup, *t*(161) = 2.10, *p* = 0.038, 95% CI [0.34, 11.38], and outgroup targets, *t*(161) = 3.08, *p* = 0.002, 95% CI [3.37, 15.42], for Christian heterosexuals, meaning that it did not relate to less attitude polarization. On the other hand, for lesbian, gay, or bisexual participants, empathic concern only related to marginally more positivity toward the ingroup, *t*(137) = 1.94, *p* = 0.055, 95% CI [−0.10, 10.07]—this also is an important finding, affirming the arguments that empathy can be biased, being directed solely to ingroup others^62^, potentially exacerbating attitude polarization via increasing ingroup love^63^. Finally, we found no reliable relationships between need for cognitive closure and intergroup bias, aside from a marginal effect of positivity toward the ingroup targets for lesbian, gay, or bisexual participants, *t*(137) = −1.68, *p* = 0.094, 95% CI [−10.02, 0.80]. Altogether the tests replicated and extended the findings, while also showing that wise reasoning uniquely predicts more positivity and less attitude polarization for both minority and majority groups in a conflict, over and above other comparison psychological constructs.

### Meta-analytic interim summary

We conducted two meta-analytic tests estimating the true effect sizes of the naturalistic relations between wise reasoning (measured with the SWIS scale) and (i) positivity to outgroups, and ii) less intergroup attitude polarization. We have no file drawer studies on these relations^64^. For comparability, we used only the data from the studies examining nominal groups (i.e., Study 2 data was excluded from the analysis) (*N* = 1,218, *k* = 7). First, we found that wise reasoning had a small-to-medium size association with positivity toward outgroups, *r* = 0.21, *p* < 0.0001, 95% CI [0.15, 0.27]. A test of heterogeneity indicated that the size of association did not differ across studies that were conducted in different groups, or conflicts, *Q*(6) = 7.38, *p* = 0.287. Second, we found significant polarization across studies for those with weaker wise reasoning, *d* = −1.24, *p* = 0.001, 95% CI [−1.99, −0.49], but not for those with stronger wise reasoning, *d* = −0.67, *p* = 0.080, 95% CI [−1.42, 0.08]. A test of heterogeneity between the two levels of wise reasoning was significant, *Q*_*B*_ = 13.59, *p* = 0.001, suggesting that stronger wise reasoning is associated with more balanced intergroup attitudes.

### Experimental investigation

Study 6 (UK and USA, 2018) explores practical implications of wise reasoning. This experiment tested the utility of a brief online wise reasoning thought exercise that was designed to prompt wise reasoning, and also measured downstream behavioral outcomes related to attitudes toward immigrants in the UK and the USA. Participants were prescreened based on their national and ethnic identity. Given that studies have shown that non-Hispanic whites were less likely to self-identify as immigrants^65,66^, and that decades of research has examined white nationals’ attitude toward immigrants^67^, Study 6 focused on white citizens from the respective countries as participants. Study 6 was a between-subjects experiment. We randomly assigned 776 participants who self-identified as white UK or USA nationals to a wise reasoning thought exercise in the Experimental Condition (WRE; *n* = 363) vs. a Control Condition with no thought exercise (*n* = 413). Given the politicized nature of attitudes towards immigration we also controlled for political orientation. For behavioral outcomes, we included two behavioral intention measures (motivation for intergroup contact and endorsement of anti-immigration policies) and two behavioral measures (sign-up for volunteer opportunities and donation to charities that assist new immigrants).

**Fig. 5:**
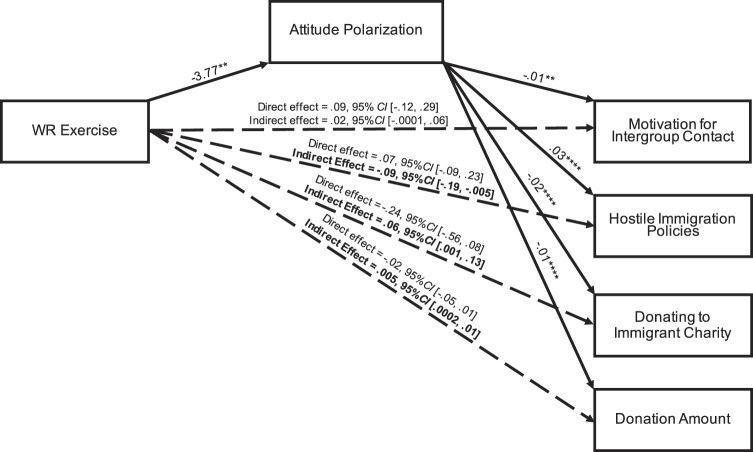
Results of Study 6: mediation models with wise reasoning exercise as the predictor, attitude polarization as the mediator, and outcome variables as the criterion variables. Unstandardized coefficients. Mediation tests were conducted separately—graphics are condensed for simplicity. Significant indirect effects are bolded.

The results generally supported our hypothesis. The pattern of results was stable whether or not we controlled for location (UK or USA) and political orientation. The manipulation check showed that participants in the wise reasoning exercise had stronger wise reasoning (measured with the SWIS scale) (WRE: *M* = 3.52, SD = 0.74; Control: *M* = 3.18, SD = 0.83), *t*(773) *=* 5.95, *p* < 0.001, *η*^2^_*p*_ = 0.04, 95% CI [0.23, 0.45]. Examining the hypotheses, we submitted feelings toward the ingroup and outgroup targets to a mixed-model regression with Condition as the moderator. As in the previous studies, we found a significant interaction, showing significantly less attitude polarization in the WRE Condition compared with the Control Condition, *F*(1, 754) = 4.75, *p* = 0.030, *η*^2^_*p*_ = 0.01. Analysis looking at the target groups individually revealed that reduced polarization resulted from a combination of trends toward i) more positivity toward the outgroup immigrants, *t* = 1.53, *p* = 0.126, *η*^2^_*p*_ = 0.003, 95% CI [−5.72, 0.71] and (ii) less positivity toward the ingroup home citizens, *t* = −1.27, *p* = 0.204, *η*^2^_*p*_ = 0.002, 95% CI [−0.83, 3.89]. The wise reasoning exercise had no robust main effects on intentional and behavioral outcomes (aside from a marginal negative effect on subscribing to volunteer; see Supplementary Table 3). However, attitude polarization was negatively related to willingness to meet outgroups, likelihood of opting to donate to charities that assist recent immigrants, and actual amount donated to the charities, and was positively related to support for anti-immigration policies (*p*s < 0.01); it was not significantly related to subscribing to volunteer opportunities (*p* = 0.063; see Supplemental Analyses). As such, we conducted four mediation tests to examine whether the WRE (vs. Control) had indirect effects on outcome variables through reduced attitude polarization using the PROCESS macro for SPSS (“Model 4” with 10,000 bootstrapped samples)^68^. We found significant indirect effects of the wise reasoning exercise via polarization on less support for hostile immigration policies, stronger propensity to donate, and greater amount donated to charities that assist new immigrants (Fig. 5). We did not find mediation for willingness to meet with outgroup members or subscribing to volunteer. We found mediation for all outcome variables when not controlling for UK vs. USA sample and political orientation. Altogether the results suggest that wise reasoning can be increased via brief online exercise, and that doing so is associated with change in attitude polarization and positive behaviors.

### Further experimental evidence during the COVID-19 pandemic

Beyond generalizing the findings from Study 6 into a different context, Study 7 (USA, 2020) aims to strengthen the study design and examine a potential confound. It is possible that any thinking (wise reasoning-related or otherwise) can reduce intergroup bias, and perhaps the wise reasoning thought exercise simply cued more thinking. Study 7 therefore explores the incremental validity of wise reasoning, over and above that of a general reflection process. It does so by introducing an Active Control (AC) Condition that cues non-wise reasoning related reflection and writing (e.g., What is your stance on this situation?) that involves deliberation processes orthogonal to wise reasoning. We compared the Wise Reasoning Exercise (WRE) against this AC Condition and a Pure Control (PC) Condition that is identical to the Control Condition in Study 6.

Study 7 was conducted in the context of the COVID-19 in the USA. Amid the debates and protests about lockdowns and the drastic change in lifestyle, some countries such as the USA showed a spike in negativity toward Chinese and Asian people in general^69^. In this context, Study 7 focused on white American citizens’ attitudes toward their ingroup (i.e., American citizens; whites in the USA) and the perceived outgroup (i.e., Chinese citizens; Asians in the USA).

This study was preregistered (https://osf.io/yzx7e). We hypothesized that participants in the WRE Condition (vs. Controls) would show strongest wise reasoning and the least attitude polarization. Based on pilot testing, we also expected that political orientation would predict intergroup attitude polarization, with increasing conservatism associated with stronger polarization. We hypothesized that the WRE would moderate the effect of conservatism on intergroup polarization such that the effect would be attenuated in the WRE Condition. We recruited 903 participants. Non-white, non-citizen or permanent residents, and those living outside of the USA were excluded from analysis, leaving 791 cases: WRE (*n* = 238); AC (*n* = 271); PC (*n* = 282). For the hypotheses that were preregistered as unidirectional (e.g., WRE Condition leads to stronger wise reasoning compared to Control Conditions), 1-tailed tests are appropriate^70,71^; thus, when applicable we present results with both 1-tailed and 2-tailed significance tests^72^.

Consistent with our prediction, the WRE Condition (*M* = 3.52, SD = 0.70) showed significantly stronger wise reasoning (measured with the SWIS scale) than both AC (*M* = 3.25, SD = 0.80; *t*(788) = 3.87, *p*_1- & 2-tailed_ < 0.001, *η*^2^_*p*_ = 0.02, 97.5% CI [0.11, 0.43]) and PC (*M* = 3.05, SD = 0.84; *t*(788) = 6.84, *p*_1- & 2-tailed_ < 0.001, *η*^2^_*p*_ = 0.06, 97.5% CI [0.32, 0.63]) Conditions. As such, for parsimony and maximal power of the analyses we present results combining the Control Conditions in the following as preregistered. More detailed analyses are presented in the Supplemental Analyses; varied forms of analyses yield similar conclusions (Supplementary Table 9).

Examining the effects of the conditions on intergroup bias, we conducted mixed-model regressions (Table 1). First, we found a non-significant Condition × Target Group interaction effect on intergroup attitudes in the expected direction, *F*(1, 789) = 1.932, _1-/2-tailed_ = 0.083/0.165, *η*^2^_*p*_ = 0.002. Looking at WRE’s effects on the target groups individually, we found that the weak interaction was explained by a combination of significantly stronger positivity toward the outgroup, *t* = 2.56, *p*_1-/2-tailed_ < 0.006/ = 0.011, *η*^2^_*p*_ = 0.01, 95% CI [−6.44, −0.85], coupled with a weaker trend of positivity toward the ingroup, *t* = 0.91, *p* = 0.362, *η*^2^_*p*_ = 0.001, 95% CI [−4.47, 1.64]. Importantly, participants in the Control Conditions showed strong ingroup-outgroup attitude polarization, but polarization was reduced for participants who completed the wise reasoning exercise. The findings showed partial support for the hypothesis that a wise reasoning exercise reduces attitude polarization by increasing positivity toward the outgroup.

**Table 1:** Study 7: attitude polarization as a function of Conditions and political orientation.

Study 7 (*n* = 791)	2-Way Condition × Target Group Interaction		
	*F*(1, 789) = 1.93, *p*_1-/2-tailed_ = 0.083/0.165, *η*^2^_p_ = 0.002		
	Mean positivity toward outgroup targets	Polarization	Mean positivity toward ingroup targets
WRE	66.548	*t* = 2.34, *p* = 0.020	69.540
Controls	62.906	*t* = 5.84, *p* < 0.001	68.121
AC	63.233	*t* = 4.18, *p* < 0.001	68.605
PC	62.592	*t* = 4.07, *p* < 0.001	67.656

	3-Way Political Orientation × Condition × Target Group Interaction		
WRE vs. controls (AC & PC)	*F*(1, 787) = 4.808, *p* = 0.029, *η*2_p_ = 0.006		
	Political orientation → outgroup positivity	Political orientation → ingroup positivity	Political orientation → attitude polarization
WRE	*t* = −0.41, *p* = 0.648	*t* = 5.78, *p* < 0.001	*t* = 6.02, *p* < 0.001
Controls	*t* = −3.95, *p* < 0.001	*t* = 9.38, *p* < 0.001	*t* = 13.90, *p* < 0.001

Upper partition: result of a mixed-model regression; both one-sided and two-sided tests are provided. Lower partition: result of a mixed-model regression; two-sided test is provided. WRE reduced the effect of political orientation on attitude polarization. Whereas political conservatism related to less positivity to outgroup targets in Control Conditions, whether taken combined or separately, there was no such relation in WRE. Further, whereas conservatism predicted strong positivity to ingroup targets in Control Conditions, this was generally weaker in WRE.

*WRE* Wise Reasoning Experimental Condition (*n* = 238), *Controls* Combination Of Pure Control Condition (*n* = 282) and Active Control Condition (*n* = 271), *AC* Active Control Condition, *PC* Pure Control Condition.

As expected, political conservativism was indeed a strong predictor of attitude polarization, *F*(1, 787) = 156.89, *p*_1- & 2-tailed_ < 0.001, *η*^2^_*p*_ = 0.17. Additionally, results of a mixed-model regression showed that the WRE moderated the relations between political orientation and positivity toward ingroup and outgroup, *F*(1, 787) = 4.808, *p* = 0.029, *η*^2^_*p*_ = 0.01. Unpacking the interaction effect by condition, political conservatism predicted less positivity toward the outgroup for participants in the Control Conditions, *t* = −3.95, *p* < 0.001, *η*^2^_*p*_ = 0.03, 95% CI [−2.68, −0.90], but it did not do so for participants in the WRE, *t* = −0.41, *p* = 0.648, *η*^2^_*p*_ = 0.001, 95% CI [−1.63, 1.07]. Further, whereas conservatism predicted much more positivity toward the ingroup for participants in the Control Conditions, *t* = 9.38, *p* < 0.001, *η*^2^_*p*_ = 0.14, 95% CI [3.45, 5.28], the relation was weaker for participants in the WRE, *t* = 5.78, *p* < 0.001, *η*^2^_*p*_ = 0.12, 95% CI [2.66, 5.42]. Taken together, participants in the WRE Condition showed significantly less intergroup attitude polarization, *t* = 6.02, *p* < 0.001, *η*^2^_*p*_ = 0.13, 95% CI [2.91, 5.73], than participants in the Control Conditions, *t* = 13.90 *p* < 0.001, *η*^2^_*p*_ = 0.26, 95% CI [5.29, 7.03]. These results supported our hypothesis that WRE reduced intergroup attitude polarization by attenuating the relations between political orientation and polarized attitudes in the context of the pandemic.

### Content analysis and mediation

We explored (i) potential linguistic differences in written responses between wise reasoning (WRE) and more general deliberative process (AC)^73^, and (ii) potential mediating mechanisms that could explain the WRE’s effect on attitude polarization. Initial analyses (Supplementary Table 10) showed several differences between the conditions, for example, participants in the WRE wrote more (i.e., higher word count), described more social processes, and used more tentative language (e.g., “maybe”). Among those significant differences between WRE and AC, four linguistic categories were also correlated with attitude polarization and wise reasoning (measured with the SWIS scale), including past focus (e.g., “walked”, “were”, “had”), tentative language (e.g., “perhaps”, “maybe”), use of larger words (i.e., >6 letters), and use of 3rd-person plurals (e.g., “they”, “they’d”) and were therefore tested as potential mediators of the relation between WRE and less attitude polarization.

Table 2 presents the indirect effects in the mediation models tested. First, we conducted a mediation test with all four categories simultaneously, controlling for word count, to examine which, if any, mediated the effect of WRE on attitude polarization using the PROCESS macro for SPSS (“Model 4” with 10,000 bootstrapped samples)^68^. Two of the categories emerged as mediators between WRE vs. AC and attitude polarization, namely, the use of larger words and decreased use of 3rd-person plural (*p*s < 0.01).

**Table 2:** Study 7: psycholinguistic variables as potential mediating mechanisms of the wise reasoning experimental (vs. active control) condition.

Model	Indirect effects		
	1. WRE (vs. AC) *→* mediator → attitude polarization	2. Political orientation × WRE (vs. AC) *→* mediator→ attitude polarization	
		WRE	AC
Mediators			
Past focus	*B* = 0.70, SE = 0.51, 95% CI [−1.74, 0.29]	*B* = 0.04, SE = 0.05, 95% CI [−0.06, 0.16]	*B* = 0.05, SE = 0.07, 95% CI [−0.07, 0.22]
Words > 6 letters	***B*** = **−1.29**, **SE** = **0.52, 95% CI [−2.36, −0.35]**	*B* = 0.12, *SE* = 0.10, 95% CI [−0.02, 0.37]	*B* = 0.18, SE = 0.12, 95% CI [−0.02, 0.45]
3rd-person plurals	***B*** = **−0.84**, **SE** = **0.39, 95% CI [−1.69, −0.19]**	***B*** = **0.15**, **SE** = **0.10, 95% CI [−0.01, 0.37]**	***B*** = **0.33**, **SE** = **0.16, 95% CI [0.05, 0.68]**
Tentative thinking	*B* = −0.33, SE = 0.25, 95% CI [−0.93, 0.06]	*B* = 0.02, SE = 0.07, 95% CI [−0.14, 0.17]	*B* = 0.09, SE = 0.07, 95% CI [−0.03, 0.25]

Significant effects in bold.

*WRE* Wise Reasoning Experimental Condition, *AC* Active Control Condition.

Next, we tested whether the same four linguistic categories would explain (i.e., mediate) the moderating effect of WRE on the relation between political conservatism and attitude polarization (PROCESS macro “Model 8”). One variable emerged to be a significant mediator of the WRE × Political Orientation interaction effect. Specifically, among those in the AC condition, political conservatism was related to more usage of 3rd-person plurals (e.g., they, them) in their writing (*B* = 0.34, SE = 0.08, *p* < 0.001), which in turn was associated with increased attitude polarization. In contrast, the relationship between political conservatism and the use of 3^rd^-person plurals was weaker in the WRE condition (*B* = 0.18, SE = 0.07, *p* = 0.015), and the indirect association between political conservatism and increased attitude polarization was no longer significant. However, the difference between the two indirect effects did not reach statistical significance, *B* = −0.18, SE = 0.14, 95% CI [−0.51, 0.03]. Together, the exploratory mediation analyses presented suggestive evidence that the wise reasoning exercise could be linked to reduced attitude polarization because it counters people’s thinking about the outgroup as “them”. This finding is aligned with intergroup research showing that “we-they” thinking—differentiating outgroup members as categorically different from the ingroup and as invariant to one another—to be a problematic root of intergroup bias^74,75^, and demonstrates a process by which wise reasoning reduces polarization.

## Discussion

Societal conflicts, bias, and polarization are rampant and represent increasingly important issues to understand. To this end, the current studies introduced the concept of wisdom, and wise reasoning more specifically, as a psychological factor that can promote balanced attitudes and charitability toward others during ongoing intergroup conflicts. Across five correlational studies, wise reasoning consistently related to positivity toward outgroups and less polarized, more balanced intergroup attitudes. The findings were observed in the context of different conflicts in different world regions, and on groups of differing power and status. The consistency of the effects was supported by two meta-analytic tests, suggesting that wise reasoning likely has implications for promoting intergroup relations and conflict reconciliation across different regions with people having different majority/minority group membership^2,37^. Wise reasoning also showed incremental and unique validity over and above a few other key variables in intergroup research (e.g., need for closure) and the generalized effects across group membership was unique to wise reasoning—none of the comparison variables related negatively to intergroup bias consistently across both minority and majority group in a conflict. Finally, experimental evidence provided initial support that an online wise reasoning exercise can reduce polarization in societal conflict.

This research contributes to multiple literatures related to social conflicts, such as psychology, political science, and behavioral economics. By charting the connections between the relatively independent studies of psychological wisdom and intergroup relations, these studies highlight how the millennia-old philosophical idea of wisdom^11^ is still relevant today for understanding and potentially managing contemporary and real-world human issues^2^. Further, the pattern of the associations between wise reasoning and weaker polarization adds both empirical support and theoretical depth to the understanding of “balance” or “moderation”^10^. Specifically, the results showing that stronger wise reasoning is related to less polarized responses to conflicts and target groups do not seem to imply weaker (in terms of attitude intensity) or more ambivalent or passive responses. Rather, they tend to be moderately positive and can produce actions that promote intergroup relations (e.g., donating to help minorities). These findings help clarify the nature of the impact of wise reasoning, that wise reasoning is associated with a more balanced—yet not indifferent—approach in response to social challenges.

Despite the above contributions, this research has limitations that raise questions for future research. Most notably, the wise reasoning measure used in the current research used self-report methodology. Although previous research validated the measure to be relatively free from biased responding and was validated against criterion-scored observer ratings^20^, more work is needed in developing and using performance-based measures^76^, combining multisource measures, and using field interventions to better understand the relation between wise reasoning and intergroup bias. Moreover, although the current research has identified a reliable relationship between wise reasoning and intergroup attitudes, there is still much to explore in terms of the situational specificity and generalizability of the relationship. For instance, are there situations where engaging in “wise reasoning” is unwise? One possible situation is when one is in immediate physical danger. Dire situations as such may not be common, but they unfortunately do happen. The possibility of wise reasoning backfiring raises the important question about situational contingencies of the approach. It will be a fruitful direction for future research to examine whether and when wise reasoning might backfire. A similar question regarding situational specificity and generalizability is whether or when it is wise to feel negativity toward an outgroup, or if there are situations in which polarization is beneficial. It is possible that polarized attitudes bear utility in some situations. For instance, polarization was argued to be a driver of more diverse opinions in heterogeneous groups^77^. Addressing these nuances in future research could bring important theoretical and practical implications to light. Finally, although our research attempted to include a wide range of study samples and contexts from different regions, the samples are limited to English-speaking populations that are relatively wealthy and industrialized. Future research should continue to investigate the generalizability of our findings to even more diverse populations, and to study potential cultural-specific phenomena that would advance the current theory further.

## Methods

### Recruitment and ethics

We recruited participants who self-identified as members of a given group to participate in our studies. Specifically, Study 1 recruited Chinese undergraduate students self-identified as protestors or non-protestors in Hong Kong. Study 2 recruited US residents who self-reported their identification with those who protested in Baltimore, USA. Study 3 recruited participants who self-identified as Hong Kong Chinese in Hong Kong. Study 4 recruited US residents who self-identified as Christian or conservative, or LGBTQ or liberal; these groups were recruited to represent divergent opinions on same-sex marriage rights. Study 5 recruited Canadian undergraduate students who self-identified as Christian and heterosexual or who self-identified as members of the LGBTQ community; these groups were recruited because information about participants’ political orientation was not available for prescreening. Study 6 recruited citizens in the USA and UK who self-identified as white. Study 7 recruited US citizens or permanent residents living in the US who self-identified as white. Additional key demographics of all samples are summarized in Supplementary Table. All participants provided informed consent before participation and received participation credit (Study 1, 3, and 5) or monetary compensation (Study 2, 4, 6, and 7) for taking part in the study. In each study, participants voluntarily signed up to participate in the study. In all studies, the researchers were not blind to the hypotheses, however, the studies were conducted online and not in the presence of the researchers. All studies were conducted online via Qualtrics survey software. The studies have received institutional research approvals. Specifically, Studies 1–3 were approved by the Human Participants Research Panel of the Hong Kong University of Science and Technology (Project title: “Attitudes toward social issues and conflicts”). Study 4 and 6 were approved by the Human Participants Research Panel of the Hong Kong University of Science and Technology (Project title: “A wise reasoning perspective to intergroup relations”; Grant #16601817). Study 5 was approved by the Human Research Ethics Committee at the University of Waterloo (ORE#20104). Study 7 was approved by the Office for Human Research Protections at Purdue University (IRB-2020-671; exempt category 3), and the University of Queensland and the National Statement on Ethical Conduct in Human Research of Australia (Approval#202001354). The interim meta-analyses was conducted using the aggregate-level information (i.e., correlations, means, and SDs) from Studies 1, 3, 4, and 5 noted above. No new studies or data were involved in the meta-analyses. Full details of study e., power and supplementary analyses) are reported as online Supplementary Information.

### Measuring wise reasoning

Wise reasoning was measured with the SWIS^20^, which builds on advances in event-reconstruction techniques^48,49^. Instead of asking participants to estimate their personality characteristics or general tendency to reason wisely regardless of ecology or context, the SWIS asks participants to recall concrete reasoning acts during or about an event. In the current case, after reflecting on the intergroup conflict materials, participants reported the extent of their engaging in concrete wise reasoning strategies during their reflections on the event (e.g., “Thought about the things both parties might have in common”; “Thought the situation could unfold in many different ways”; from 1 = not at all to 5 = very much). This situated approach was shown to be superior to other self-report wisdom measures in its ability to minimize self-report bias (e.g., self-presentation, memory bias) for a reliable estimate of wise reasoning that was validated against observer-rated wisdom scores specifically regarding intergroup conflict^20^, making this measure ideal for the current purpose. In the current studies, the scale demonstrated excellent reliability (12-item scale in Study 1: *α* = 0.91; full 21-item scale in Studies 2–6: *α*s > = 0.89).

### Study 6 materials

In the Wise Reasoning thought exercise we prepared participants before reading a news article by telling them that “some people report understanding societal issues (e.g., conflicts and debates) better by taking an outside perspective.” We then provided some instructions for how to take an outside perspective using question prompts that they could consider while reading the following article:

(i) “How might [*your name*]’s perspective on this situation be different from other people’s perspectives?”
(ii) “How does [*your name*] think the situation might change through time?”
(iii) “What are the uncertainties for [*your name*] surrounding this situation?”
(iv) “How does [*your name*] think that people could work together toward arrangements that make all parties happy?”

After reading the article, we asked participants to provide open-text responses to these same four questions. They then completed the rest of the survey.

Political orientation was measured with three items assessing political views on foreign policy, economic, and social policy issues, which we combined due to their high intercorrelation (*r*s > 0.68; *α* > 0.90). To probe the impact of the wise reasoning exercise through reduced attitude polarization, we used two additional self-report behavioral intention measures (i and ii) and two behavioral measures (iii and iv):

(i) *Motivation for intergroup contact*: assessed with a single item, “If you had the opportunity, to what extent would you be willing to meet with people who hold opinions very different from yours about immigration and hear their point of view?” (1 = *not at all* to 6 = *very much*).
(ii) *Endorsement of hostile immigration policies*: assessed with 11 items, e.g., “Immigrants should not share our facilities (e.g., schools; hospitals)” (1 = *strongly opposed* to 7 = *strongly in favor*). Factor analysis revealed a single-factor solution, so we combined the items (*α* = 0.96)
(iii) *Volunteering opportunities*: we presented participants with an open response box in which they could enter their email address to receive volunteer opportunities with charities that assist immigrants. We told them that they could enter an anonymous address to protect their privacy if they wished (this served to inhibit socially desirable responding). Those who provided an email were coded 1, and the rest were coded 0.
(iv) *Donating to help immigrants*: we gave participants a $.50 [£.50] bonus upon ostensibly completing the survey. Later, we told them that they could use their bonus to anonymously donate to a charity that assists new immigrants if they so wished. Participants chose one of the following two responses, which were randomized in presentation order: “Yes, I’m interested in donating” or “No, thanks.” For those who chose to donate, an open response box popped up in which they could enter any amount between $0 to $.50 [£0 to £.50]. We coded donation amount as zero for participants who chose to not donate.

### Study 7 materials

Study 7 used a similar protocol as Study 6, with the addition of an Active Control condition. First, before reading the news article, we told all participants that they would read a randomly selected news clipping and that we would ask them questions about the article later. After the news article, participants were told that, to facilitate comprehension of the article *take the perspective of the different parties involved* and *adopt a bird’s eye view* to consider the *bigger picture* (Wise Reasoning Experiment; WRE), or focus on your *instantaneous reaction* and consider your *immediate feelings* about the story you read *here and now* (Active Control; AC), or given no instructions (Pure Control; PC). Then participants in the WRE were asked to provide open-text responses to the following questions:

- Which parties are involved or affected?
- What are the different layers and perspectives of the situation?
- How might the situation change with time?
- What might be the uncertainties surrounding this situation?
- If there are ways that can help different parties work together -- toward arrangements that protect and benefit all people—what will they be?

Participants in the AC were asked to provide open-text responses to the following questions:

- What is your stance on this situation?
- What is the first reason you have for your stance?
- What is the second reason?
- What is the third reason?

Participants in the PC were not provided with questions, nor asked to provide open-text responses. All participants then proceeded to complete the rest of the survey. Political orientation was measured with three items which were combined as in Study 6 (*r*s > 0.78; *α* > 0.90).

### Reporting summary

Further information on research design is available in the Nature Research Reporting Summary linked to this article.

## Supplementary information

Supplementary Information

Reporting Summary

## Data Availability

Source data are provided with this paper. The data that support the findings of this study are available in the Open Science Framework repository with the identifier https://osf.io/r247w/. A reporting summary for this Article is available as a Supplementary Information file.

## Source data

Source Data
