# Supplementary material for: Wise reasoning, intergroup positivity, and attitude polarization across contexts
Source: Nat Commun. 2021 Jun 3;12:3313. doi: 10.1038/s41467-021-23432-1 (PMC8175723; doi:10.1038/s41467-021-23432-1)
Supplement: Supplementary file 1 — Supplementary Information [file 41467_2021_23432_MOESM1_ESM.docx]

**Supplementary Information for**

**Wise reasoning, intergroup positivity, and attitude polarization across contexts**

Contents

[Study Materials 3](#_Toc67138909)

[Situated Wise Reasoning Scale 3](#_Toc67138910)

[Wise Reasoning Exercise (Study 6) 4](#_Toc67138911)

[Hostile Policies Toward Immigrants Scale (Study 6) 7](#_Toc67138912)

[Wise Reasoning Exercise and Active Control Materials (Study 7) 8](#_Toc67138913)

[Intergroup Conflict Reflection Materials 10](#_Toc67138914)

[Donation (Study 6) 11](#_Toc67138915)

[Email Subscription (Study 6) 13](#_Toc67138916)

[Additional Demographic Information 14](#_Toc67138917)

[Supplementary Table 1. Summary of Sample Characteristics (Studies 1 to 6). 14](#_Toc67138918)

[Descriptives and Intercorrelations of Study Variables 15](#_Toc67138919)

[Supplementary Table 2. Descriptives and Intercorrelations of Variables in the Correlational Studies (Studies 1 to 5). 15](#_Toc67138920)

[Supplementary Table 3. Means, Standard Deviations, Intercorrelations of Variables in the Experimental Studies. Study 6 presented with the sub-samples both combined and separated. 17](#_Toc67138921)

[Additional Details of Study Method and Analyses 20](#_Toc67138922)

[Study 1 20](#_Toc67138923)

[Study 2 22](#_Toc67138924)

[Study 3 25](#_Toc67138925)

[Study 4 26](#_Toc67138926)

[Study 5 28](#_Toc67138927)

[Supplementary Table 4. Study 5 Results of Mixed-level 3-way Interactions. 33](#_Toc67138928)

[Supplementary Table 5A. Factor Analysis of Wise Reasoning and Perspective Taking. 35](#_Toc67138929)

[Supplementary Table 5B. Factor Analysis of Wise Reasoning and Empathic Concern 36](#_Toc67138930)

[Supplementary Table 5C. Factor Analysis of Wise Reasoning and Need for Closure 37](#_Toc67138931)

[Full details of internal meta-analysis (Studies 1-5) 38](#_Toc67138932)

[Supplementary Table 6. Means Differences in Intergroup bias in Studies 1 to 5. 39](#_Toc67138933)

[Supplementary Table 7. Studies 3 to 5 Descriptives and Intercorrelations of Wise Reasoning Dimensions and Intergroup Bias. 40](#_Toc67138934)

[Study 6 41](#_Toc67138935)

[Supplementary Table 8. Indirect and direct effects of a brief online wise-reasoning exercise on intergroup intentions and behaviors. 44](#_Toc67138936)

[Study 7 45](#_Toc67138937)

[Supplementary Table 9. Results of Study 7: Tests of attitude polarization as a function of Conditions and political orientation. 49](#_Toc67138938)

[Supplementary Table 10. Correlations between the LIWC results and key study variables. 51](#_Toc67138939)

[Supplementary Table 11. Psycholinguistic variables as potential mediating mechanisms of the wise reasoning experimental (vs. active control) condition. 53](#_Toc67138940)

[Supplementary References 54](#_Toc67138941)

# **Study Materials**

# Situated Wise Reasoning Scale ^1^

Instructions

Thank you for your thoughts in the previous section.

As you reflected on [the conflict], to what extent did you engage in the following thoughts and behaviors? Note that none of the statements listed below are supposed to be "good" or "bad". We are simply interested in how people approach difficult situations. Please select the extent to which you engaged in the following thoughts and behaviors:

"While I was contemplating and writing about the previous scenario, I did the following..."

(from 1 – *not at all*, to 5 – *very much*)

1. Put myself in both parties’ shoes
2. Thought about the things both parties might have in common^*^
3. Made an effort to take both parties’ perspective
4. Took time to consider both parties’ opinions on the matter before coming to a conclusion^*^
5. Looked for different solutions to the evolving conflict
6. Considered alternative solutions as I learned about the conflict^*^
7. Believed the situation could lead to a number of different outcomes
8. Thought the situation could unfold in many different ways^*^
9. Double-checked whether my opinion on the situation might be incorrect
10. Considered whether the other party’s opinions might be correct^*^
11. Looked for any extraordinary circumstances before forming my opinion^*^
12. Behaved as if there may be some information to which I do not have access
13. Tried my best to find a way to accommodate both parties’ perspectives^*^
14. Though it may not have been possible, I searched for solutions that could result in both parties being satisfied^*^
15. Considered first whether a compromise was possible in resolving the situation
16. Viewed it as very important that the parties resolve the situation^*^
17. Tried to anticipate how the conflict might be resolved^*^
18. Wondered what I would think if I were somebody else considering the situation
19. Tried to see the conflict from the point of view of an uninvolved person^*^
20. Asked myself what other people might think or feel if they were considering the conflict
21. Thought about whether an outside person might have a different opinion from mine about the situation^*^

*Note*. ^*^ 12 items included in the short version of the scale (Study 1).

## Wise Reasoning Exercise (Study 6)

Instructions *Before* News Article:

Social Issue News Article

In the following, you will be presented with a news article on a recent and ongoing social issue. Please spend some time reading the information. You will then respond to some questions regarding the issue.

Some people report understanding societal issues (e.g., conflicts and debates) better by taking an outside perspective. Please take yourself outside of the situation, and ask “How would different people or groups think and feel in this situation?”

To help you to **take an outside perspective**, use third-person pronouns (e.g., he/she/your name) as much as possible as you contemplate the issue. For example, as you read the following article, ask yourself, as an outsider looking in:

- “How might **[*your name*]**’s perspective on this situation be different from other people’s perspectives?”
- “How does **[*your name*]** think the situation might change through time?”
- “What are the uncertainties for **[*your name*]** surrounding this situation?”
- “How does **[*your name*]** think that people could work together toward arrangements that make all parties happy?”

This page is timed at 35 seconds, to ensure enough time to consider these instructions.

Instructions *After* News Article:

Social Issue News Article

Please continue to think about the situation presented in the article, from an outside perspective. Type out your thoughts as you are thinking. Use the pronouns he/she/your name as you type. For example, “[your name] thinks that …” or “His/her feelings about this matter are …”

Use these questions to guide your responses:

1. How might [your name]'s perspective on this situation be different from other people's perspectives?


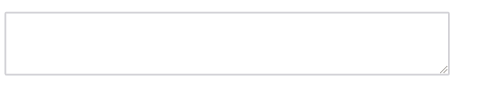


1. How does [your name] think the situation might change through time?


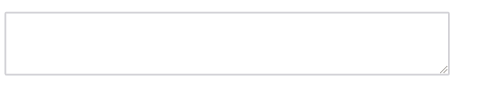


(Continued next page)

Social Issue News Article

Please continue to think about the situation presented in the article, from an outside perspective. Type out your thoughts as you are thinking. Use the pronouns he/she/your name as you type. For example, “[your name] thinks that …” or “His/her feelings about this matter are …”

Use these questions to guide your responses:

1. What are the uncertainties for [your name] surrounding this situation?
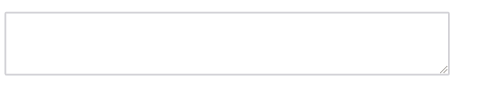

2. How does [your name] think that people could work together toward arrangements that make all parties happy?


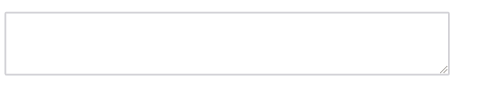


## Hostile Policies Toward Immigrants Scale (Study 6)

1 = *Strongly oppose* to 7 = *Strongly in favor*

1. Americans’/ Brits’ taxes should not be used to help immigrants.
2. Immigrants should be excluded from social welfare.
3. We should scale down immigrant admissions.
4. Immigrants should have limited rights.
5. We should only accept immigrants who speak our language.
6. Only immigrants who identify with our culture and values should be allowed in the country.
7. Immigrants should not share our facilities (e.g., schools; hospitals).
8. Immigrants should not be allowed to own land.
9. Immigrants' job applications should be given lower priority than Americans’/ Brits’^c^.
10. We should not provide immigrants with special assistance (e.g., money, computer, phone).
11. Immigrants should not be allowed to criticize our country in any way.

*Note*. All items were reversed coded.

## Wise Reasoning Exercise and Active Control Materials (Study 7)

Instructions *Before* News Article for both conditions:

News Article

In the following, you will read a randomly selected news clip. Later, we will ask you questions about the article. Please read the article carefully.

Instructions *After* News Article (Wise Reasoning Exercise):

**Thoughts on the News Article**

To facilitate comprehension of the article, **take the perspective of the different parties involved** and **adopt a bird's eye view** to consider the **bigger picture**. This involves asking the questions, "How do the different parties think about the situation?", and "How do the different parties feel about the situation?"

Use these questions to guide your written response below:

- Which parties are involved or affected?
- What are the different layers and perspectives of the situation?
- How might the situation change with time?
- What might be the uncertainties surrounding this situation?
- If there are ways that can help different parties work together -- toward arrangements that protect and benefit all people -- what will they be?

**Please write at least 2 paragraphs and devote at least 3 minutes to the task.**


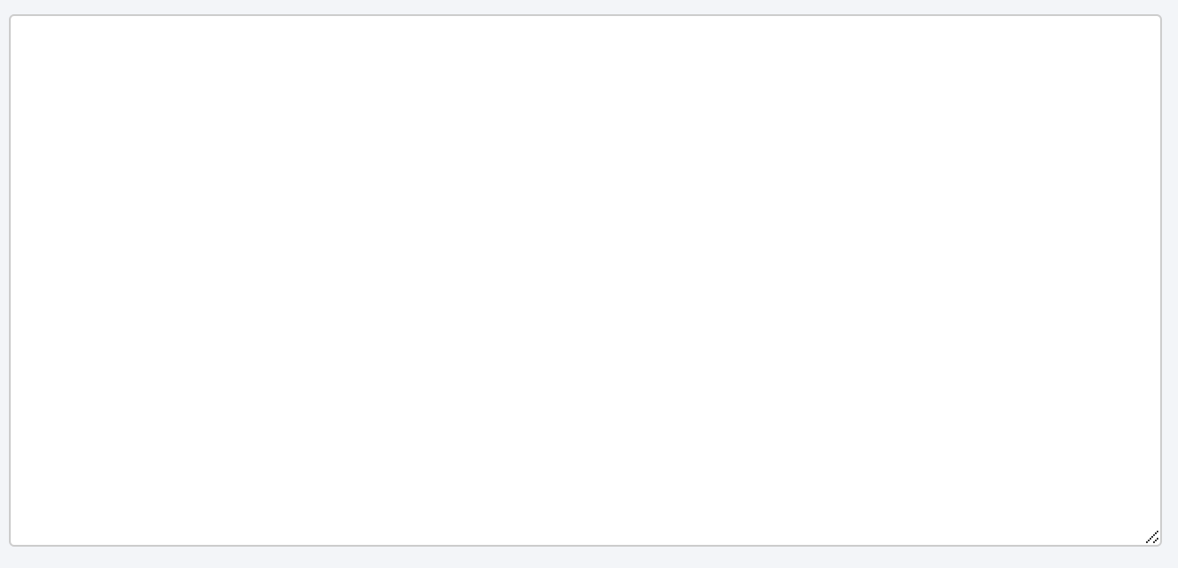


Instructions *After* News Article (Active Control):

**Thoughts on the News Article**

To facilitate comprehension of the article, focus on your **instantaneous reaction** and consider your **immediate feelings** about the story you read **here and now**. This involves asking the questions, "How do I think about this situation?", and "How do I feel about the situation?"

Use these questions to guide your written response below:

- What is your stance on this situation?
- What is the first reason you have for your stance?
- What is the second reason?
- What is the third reason?

**Please write at least 2 paragraphs and devote at least 3 minutes to the task.**


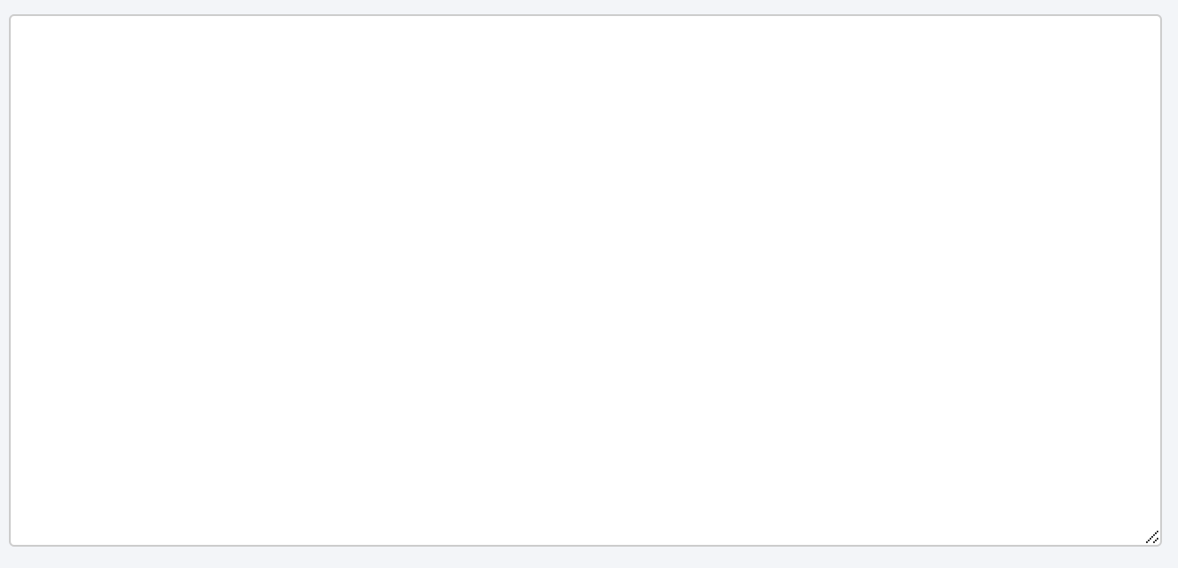


## Intergroup Conflict Reflection Materials

All materials can be found at <https://osf.io/r247w/>

## Donation (Study 6)

#### Instructions for the American Sample

Bonus Payment

This is the last part of the survey. As an appreciation, we are providing a **$.50 bonus** to participants who complete this survey. If you like, this bonus may be used to donate to [International Rescue Committee](https://www.rescue.org/article/how-help-refugees-united-states-12-ways-stand-welcome), a US-based global charity since 1933, offering support and accommodation to vulnerable immigrants and refugees in the United States and enabling them to become successful members of the communities.

Of course, you may keep your bonus and you are under no obligation to donate - your decision will be kept anonymous, as all information you provide in this survey.

[IRC Logo was displayed here in the actual survey]

Would you be interested in making a donation to International Rescue Committee?

- No, thanks.
- Yes, I’m interested in donating.

[if Yes was selected, the below question appeared immediately]

How much would you like to donate ($0 to $.50)?

(Enter the numerical amount here; we will deduct it from the bonus in your payment)

#### Instructions for the UK Sample

Bonus Payment

The is the last part of the survey. As an appreciation, we are providing a **£.50 bonus** to participants who complete this survey. If you like, this bonus may be used to donate to [**Migrant Help**](https://www.migranthelpuk.org/our-vision)**,**a national charity in the UK since 1963, offering support and accommodation to vulnerable migrants and refugees and enabling them to become successful members of the communities.

Of course, you may keep your bonus and you are under no obligation to donate - your decision will be kept anonymous, as all information you provide in this survey.

​ [MH Logo was displayed here in the actual survey]

Would you be interested in making a donation to Migrant Help?

- Yes, I’m interested in donating.
- No, thanks.

[if Yes was selected, the below question appeared immediately]

How much would you like to donate (£0 to £.50)?

(Enter the numerical amount here; we will deduct it from the bonus in your payment)

## Email Subscription (Study 6)

#### Instructions for the American Sample

**To receive volunteer opportunities**with charities like International Rescue Committee that assist immigrants and refugees in the US, **subscribe** by entering your email address below:

(you may choose to enter an anonymous email address to protect your privacy)


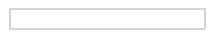


#### Instructions for the UK Sample

**To receive volunteer opportunities**with national charities like Migrant Help that assist immigrants and refugees in the UK, **subscribe** by entering your email address below:

(you may enter the anonymous email address provided by Prolific to protect your privacy)


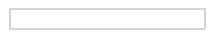


# Additional Demographic Information

### Supplementary Table 1. Summary of Sample Characteristics (Studies 1 to 6).

|  | **Study 1**  **Hong Kong** | **Study 2**  **USA** | **Study 3**  **Hong Kong** | **Study 4**  **USA** | **Study 5**  **Canada** | **Study 6**  **USA** | **Study 6**  **UK** | **Study 7**  **USA** |
| --- | --- | --- | --- | --- | --- | --- | --- | --- |
| Sample *n* | 74 | 337 | 298 | 243 | 308 | 221 | 555 | 791 |
| Age_mean_ (*SD*) | 19.1 (.80) | 36.9 (12.9) | 19.8 (1.0) | 35.37 (12.46) | 20.7 (5.0) | 36.03 (10.57) | 39.42 (15.31) | 34.43 (12.47) |
| Gender (f/m/other) | 39/35 | 194/136/4 | 164/134 | 145/98/0 | 231/77 | 119/100/2 | 361/190/4 | 406/379/6 |
| Race (%)^a^ |  |  |  |  |  |  |  |  |
| Asian | 100 | 3.3 | 100 | 5.8 | 33.1 | - | - | - |
| African/Black | - | 24.9 |  | 5.3 | 7.5 | - | - | - |
| White | - | 66.7 |  | 81.5 | 55.8 | 100 | 100 | 100 |
| Hispanic | - | 2.1 |  | 4.1 | 1.3 | - | - | - |
| “Other” | - | 3.0 |  | 3.2 | 5.2 | - | - | - |
| Education (%) |  |  |  |  |  |  |  |  |
| High school or less | - | 11.9 |  | 11.1 | - | 5.0 | 35.2 | 9.0 |
| Some college | 100 | 29.5 | 100 | 29.2 | 100 | 14.0 | 15.1 | 23.9 |
| College | - | 44.4 |  | 40.3 | - | 28.1 | 36.6 | 46.9 |
| Post-grad | - | 14.3 |  | 19.3 | - | 53.0 | 13.1 | 20.3 |

*Note*. In Study 2, 3 participants did not report gender, 4 did not report race, and 8 did not report education. In Studies 4, 5 and 6, some participants identified with multiple racial groups. In Study 6 (UK sample), 3 participants did not report gender and 2 participants did not report race.

# Descriptives and Intercorrelations of Study Variables

### Supplementary Table 2. Descriptives and Intercorrelations of Variables in the Correlational Studies (Studies 1 to 5).

|  |  |  | Correlations | | | | | | |  | |  | |  | |
| --- | --- | --- | --- | --- | --- | --- | --- | --- | --- | --- | --- | --- | --- | --- | --- |
| **Variables** | Mean | SD | 1 | 2 | 3 | 4 | 5 |  |  | |  | |  | |  |
| ***Study 1*** |  |  |  |  |  |  |  |  |  | |  | |  | |  |
| 1. Age | 19.16 | 2.49 |  |  |  |  |  |  |  | |  | |  | |  |
| 2. Female (vs. male) | 1.49 | .53 | .14 |  |  |  |  |  |  | |  | |  | |  |
| 3. Non-protester (vs. protester) | .49 | .50 | .25^*^ | -.01 |  |  |  |  |  | |  | |  | |  |
| 4. Warmth | 4.56 | 1.33 | -.02 | -.02 | -.23^**^ |  |  |  |  | |  | |  | |  |
| 5. Trust | 4.69 | 1.08 | -.06 | -.06 | -.20 | .80 |  |  |  | |  | |  | |  |
| 6. Wise reasoning | 3.57 | .51 | -.06 | -.06 | .02 | -.29^**^ | .28^**^ |  |  | |  | |  | |  |
|  | Mean | SD | 1 | 2 | 3 | 4 | 5 | 6 | 7 | | 8 | | 9 | | 10 |
| ***Study 2*** |  |  |  |  |  |  |  |  |  | |  | |  | |  |
| 1. Age | 36.85 | 12.86 |  |  |  |  |  |  |  | |  | |  | |  |
| 2. Female (vs. male) | 1.60 | .51 | .05 |  |  |  |  |  |  | |  | |  | |  |
| 3. Income | 41,000-50,000 (median) |  | .06 | .02 |  |  |  |  |  | |  | |  | |  |
| 4. Education | College degree (median) |  | .07 | .03 | .25^***^ |  |  |  |  | |  | |  | |  |
| 5. Race (Black/Other) | .73 | .45 | .18^**^ | -.03 | .06 | -.03 |  |  |  | |  | |  | |  |
| 6. Identification w/ protesters | 3.45 | 1.87 | -.18^***^ | -.00 | -.13^*^ | -.43^***^ | .12^*^ |  |  | |  | |  | |  |
| 7. Warmth | 3.37 | 1.72 | .10 | -.04 | .14^*^ | .27^***^ | < .01 | -.37^***^ |  | |  | |  | |  |
| 8. Trust | 3.52 | 1.73 | .16^**^ | -.03 | .14^*^ | .25^***^ | .01 | -.39^***^ | .89^***^ | |  | |  | |  |
| 9. Positive emotion | 3.11 | 1.75 | .08 | -.10 | .14^*^ | .18^**^ | -.03 | -.29^***^ | .73^***^ | | .73^***^ | |  | |  |
| 10. Negative emotion | 3.60 | 1.80 | -.19^***^ | < .01 | -.12^*^ | -.30^***^ | .03 | .55^***^ | -.55^***^ | | -.54^***^ | | -.42^***^ | |  |
| 11. Wise reasoning | 3.38 | .79 | -.05 | -.06 | -.06 | -.07 | -.03 | .17^**^ | .17^**^ | | -.05 | | .06 | | .10 |
|  | Mean | SD | 1 | 2 | 3 | 4 |  |  |  | |  | |  | |  |
| ***Study 3*** |  |  |  |  |  |  |  |  |  | |  | |  | |  |
| 1. Age | 19.75 | .96 |  |  |  |  |  |  |  | |  | |  | |  |
| 2. Female (vs. male) | 1.55 | .50 | -.10 |  |  |  |  |  |  | |  | |  | |  |
| 3. Feeling to HK Chinese | 66.51 | 18.39 | -.11 | -.02 |  |  |  |  |  | |  | |  | |  |
| 4. Feeling to ML Chinese | 43.08 | 18.68 | -.03 | -.02 | .23^***^ |  |  |  |  | |  | |  | |  |
| 5. Wise reasoning | 3.56 | .59 | -.07 | .02 | .03 | .25^***^ |  |  |  | |  | |  | |  |
|  | Mean | SD | 1 | 2 | 3 | 4 | 5 | 6 | 7 | | 8 | | 9 | |  |
| ***Study 4*** |  |  |  |  |  |  |  |  |  | |  | |  | |  |
| 1. Age | 35.37 | 12.46 |  |  |  |  |  |  |  | |  | |  | |  |
| 2. Female (vs. male) | 1.60 | .49 | .01 |  |  |  |  |  |  | |  | |  | |  |
| 3. Education | College degree (median) |  | .03 | -.02 |  |  |  |  |  | |  | |  | |  |
| 4. Political conservatism | 3.05 | 1.92 | .17^**^ | -.14^*^ | -.09 |  |  |  |  | |  | |  | |  |
| 5. Conservatives (vs. Liberals) | .59 | .49 | .15^*^ | -.14^*^ | .02 | .63^***^ |  |  |  | |  | |  | |  |
| 6. Feeling to Christians | 50.32 | 30.20 | .17^**^ | .06 | .02 | .51^***^ | .64^***^ |  |  | |  | |  | |  |
| 7. Feeling to Gays | 69.92 | 29.53 | -.18^***^ | .25^***^ | .09 | -.58^***^ | -.41^***^ | -.44^***^ |  | |  | |  | |  |
| 8. Wise reasoning | 2.73 | 0.80 | -.06 | -.07 | .10 | .03 | .08 | .03 | .11 | |  | |  | |  |
|  | Mean | SD | 1 | 2 | 3 | 4 | 5 | 6 | 7 | | 8 | | 9 | |  |
| ***Study 5*** |  |  |  |  |  |  |  |  |  | |  | |  | |  |
| 1. Age | 20.73 | 4.96 |  |  |  |  |  |  |  | |  | |  | |  |
| 2. Female (vs. male) | 1.75 | .43 | -.02 |  |  |  |  |  |  | |  | |  | |  |
| 3. Christian (vs. LGBTQ) | .46 | .50 | -.10 | .08 |  |  |  |  |  | |  | |  | |  |
| 4. Perspective taking | 3.66 | .64 | .12^*^ | .11 | -.11 |  |  |  |  | |  | |  | |  |
| 5. Empathic concern | 3.90 | .70 | .09 | .30^***^ | -.13^*^ | .43^***^ |  |  |  | |  | |  | |  |
| 6. Need for closure | 3.93 | .70 | -.09 | .17^**^ | -.03 | -.10 | .05 |  |  | |  | |  | |  |
| 7. Feeling to Christians | 61.36 | 26.23 | .06 | -.01 | -.38^***^ | .02 | .15^**^ | -.01 |  | |  | |  | |  |
| 8. Feeling to homosexuals | 71.43 | 24.97 | .09 | .18^***^ | .26^***^ | .18^**^ | .15^**^ | -.05 | -.22^***^ | |  | |  | |  |
| 9. Same-sex marriage | 3.81 | .98 | .09 | .16^**^ | .25^***^ | .24^***^ | .13^*^ | -.05 | .71^***^ | | -.35^***^ | |  | |  |
| 10. Wise reasoning | 3.34 | .65 | -.02 | -.02 | -.15^**^ | .32^***^ | .24^***^ | .03 | .05 | | .16^**^ | | .07 | |  |

*Note*. Pearson’s correlations; all tests two-sided. Due to administrative error, Study 5 income data was not collected in the control condition.

^***^ *p* < .001, ^**^ *p* < .01, and ^*^ *p* < .05.

### Supplementary Table 3. Means, Standard Deviations, Intercorrelations of Variables in the Experimental Studies. Study 6 presented with the sub-samples both combined and separated.

|  | Mean | SD | 1 | 2 | 3 | 4 | 5 | 6 | 7 | 8 | 9 | 10 | 11 | 12 | 13 |
| --- | --- | --- | --- | --- | --- | --- | --- | --- | --- | --- | --- | --- | --- | --- | --- |
| ***Study 6*** |  |  |  |  |  |  |  |  |  |  |  |  |  |  |  |
| 1. Age | 38.46 | 14.20 |  |  |  |  |  |  |  |  |  |  |  |  |  |
| 2. Female (vs. male) | 1.63 | .49 | .03 |  |  |  |  |  |  |  |  |  |  |  |  |
| 3. Income | See below |  | -.04 | -.04 |  |  |  |  |  |  |  |  |  |  |  |
| 4. Education | College degree  (median) |  | -.10^**^ | -.12^***^ | .35^***^ |  |  |  |  |  |  |  |  |  |  |
| 5. Political Orientation | 3.96 | 1.32 | .17^***^ | -.07^*^ | -.01 | -.16^***^ |  |  |  |  |  |  |  |  |  |
| 6. Condition (WRE vs. Control) | .47 | .50 | -.04 | .02 | .01 | .01 | -.01 |  |  |  |  |  |  |  |  |
| 7. Feeling to U.S.citizens | 79.22 | 16.79 | .09^*^ | .03 | .07 | -.06 | .08^*^ | -.04 |  |  |  |  |  |  |  |
| 8. Feeling to immigrants | 58.27 | 25.64 | -.06 | -.01 | .12^***^ | .23^***^ | -.42^***^ | .05 | .11^**^ |  |  |  |  |  |  |
| 9. Intergroup contact motivation | 4.20 | 1.46 | -.02 | -.10^**^ | .00 | .07 | -.06 | .04 | .12^**^ | .20^***^ |  |  |  |  |  |
| 10. Hostile Policies | 3.55 | 1.58 | .11^**^ | .01^*^ | -.13^***^ | -.25^***^ | .51^***^ | -.01 | .03 | -.68^***^ | -.15^***^ |  |  |  |  |
| 11. Subscription to volunteer | .16 | .37 | .00 | .05 | -.05^*^ | .08^*^ | -.14^***^ | -.07 | .06 | .17^***^ | .18^***^ | -.19^***^ |  |  |  |
| 12. Donation N/Y | .29 | .45 | .04 | .06 | .01^*^ | .09^*^ | -.17^***^ | -.04 | .01 | .25^***^ | .10^**^ | -.27^***^ | .24^***^ |  |  |
| 13. Donation amount | .13 | .21 | .06 | .08^*^ | .04 | .08^*^ | -.17^***^ | -.04 | .04 | .25^***^ | .10^**^ | -.26^***^ | .24^***^ | .95^***^ |  |
| 14. Wise reasoning | 3.34 | .81 | -.11^**^ | -.05 | .07^*^ | .06 | -.10 | .21^***^ | .14^***^ | .29^***^ | .40^***^ | -.22^***^ | .12^**^ | .07^*^ | .06 |
|  | Mean | SD | 1 | 2 | 3 | 4 | 5 | 6 | 7 | 8 | 9 | 10 | 11 | 12 | 13 |
| ***USA subset*** |  |  |  |  |  |  |  |  |  |  |  |  |  |  |  |
| 1. Age | 36.03 | 10.58 |  |  |  |  |  |  |  |  |  |  |  |  |  |
| 2. Female (vs. male) | 1.55 | .51 | -.01 |  |  |  |  |  |  |  |  |  |  |  |  |
| 3. Income | 50,000-59,000  (median) |  | .03 | -.02 |  |  |  |  |  |  |  |  |  |  |  |
| 4. Education | College degree  (median) |  | -.04 | -.11 | .32^***^ |  |  |  |  |  |  |  |  |  |  |
| 5. Political Orientation | 3.47 | 1.73 | .18^*^ | -.16^*^ | .06 | .01 |  |  |  |  |  |  |  |  |  |
| 6. Condition (WRE vs. Control) | .48 | .50 | -.04 | .09 | -.01 | .05 | -.05 |  |  |  |  |  |  |  |  |
| 7. Feeling to U.S.citizens | 80.14 | 17.88 | .14^*^ | .03 | .11 | -.13 | .11 | .05 |  |  |  |  |  |  |  |
| 8. Feeling to immigrants | 70.66 | 23.71 | .02 | .11 | -.04 | .03 | -.50^***^ | .11 | .28^***^ |  |  |  |  |  |  |
| 9. Intergroup contact motivation | 4.24 | 1.48 | .00 | -.14^*^ | .05 | .11 | .06 | .09 | .10 | .26^***^ |  |  |  |  |  |
| 10. Hostile Policies | 2.76 | 1.60 | .04 | -.16^*^ | -.01 | -.09 | .66^***^ | -.07 | -.02 | .56^***^ | -.17* |  |  |  |  |
| 11. Subscription to volunteer | .16 | .37 | .03 | .08 | -.15^*^ | .06 | -.08 | -.01 | .02 | .19^**^ | .15* | -.13^*^ |  |  |  |
| 12. Donation N/Y | .31 | .46 | .02 | .08 | -.14^*^ | .08 | -.19^**^ | .09 | -.01 | .22^**^ | .13 | -.24^***^ | .34^***^ |  |  |
| 13. Donation amount | .13 | .21 | .06 | .09 | -.09 | .08 | -.21^**^ | .07 | .03 | .20^**^ | .11 | -.24^***^ | .29^***^ | .94^***^ |  |
| 14. Wise reasoning | 3.56 | .84 | -.07 | -.01 | .02 | -.01 | .08 | .18^**^ | .24^***^ | .21^***^ | .45^***^ | -.12 | .13^*^ | .06 | .03 |
|  | Mean | SD | 1 | 2 | 3 | 4 | 5 | 6 | 7 | 8 | 9 | 10 | 11 | 12 | 13 |
| ***UK subset*** |  |  |  |  |  |  |  |  |  |  |  |  |  |  |  |
| 1. Age | 38.73 | 15.05 |  |  |  |  |  |  |  |  |  |  |  |  |  |
| 2. Female (vs. male) | 1.64 | .48 | .03 |  |  |  |  |  |  |  |  |  |  |  |  |
| 3. Income | 30,000-39,000  (median) |  | -.02 | -.01 |  |  |  |  |  |  |  |  |  |  |  |
| 4. Education | College degree  (median) |  | -.06 | -.07 | .23^***^ |  |  |  |  |  |  |  |  |  |  |
| 5. Political Orientation | 4.15 | 1.06 | .16 | -.06 | .06 | -.10^*^ |  |  |  |  |  |  |  |  |  |
| 6. Condition (WRE vs. Control) | .46 | .50 | -.04 | -.01 | .01 | -.02 | .03 |  |  |  |  |  |  |  |  |
| 7. Feeling to UK citizens | 78.85 | 16.34 | .08^*^ | .04 | .03 | -.07 | .09^*^ | -.08 |  |  |  |  |  |  |  |
| 8. Feeling to immigrants | 53.32 | 24.72 | -.04 | -.01 | .08 | .13^**^ | -.31^***^ | .02 | .04 |  |  |  |  |  |  |
| 9. Intergroup contact motivation | 4.19 | 1.45 | -.02 | -.09^*^ | -.03 | .05 | -.13^**^ | .02 | .13^**^ | .19^***^ |  |  |  |  |  |
| 10. Hostile policies | 3.87 | 1.47 | -.09^*^ | .04 | -.07 | .13^**^ | .37^***^ | .02 | .07 | .68^***^ | -.14^***^ |  |  |  |  |
| 11. Subscription to volunteer | .16 | .37 | -.01 | .04 | .00 | .09^*^ | -.18^***^ | -.10^*^ | .08 | .17^***^ | .20^***^ | -.23^***^ |  |  |  |
| 12. Donation N/Y | .28 | .45 | .05 | .06 | .08* | .09^*^ | -.16^***^ | -.09^*^ | .27^***^ | .27^***^ | .09^*^ | -.29^***^ | .20^***^ |  |  |
| 13. Donation amount | .12 | .21 | .07 | .08 | .11* | .08 | -.15^***^ | -.08 | .04 | .27^***^ | .10^*^ | -.28^***^ | .22^***^ | .95^***^ |  |
| 14. Wise reasoning | 3.25 | .78 | -.10^*^ | -.04 | .03 | -.03 | -.15^***^ | .22^***^ | .09^*^ | .27^***^ | .38^***^ | .20^***^ | .11^**^ | .08^**^ | .08 |
|  | Mean | SD | 1 | 2 | 3 | 4 | 5 | 6 | 7 | 8 | 9 | 10 | 11 | 12 | 13 |
| **Study 7** |  |  |  |  |  |  |  |  |  |  |  |  |  |  |  |
| 1. Age | 34.43 | 12.47 |  |  |  |  |  |  |  |  |  |  |  |  |  |
| 2. Female (vs. male) | 1.49 | .52 | -.04 |  |  |  |  |  |  |  |  |  |  |  |  |
| 4. Education | College degree  (median) |  | .18^***^ | -.05 |  |  |  |  |  |  |  |  |  |  |  |
| 5. Political Orientation | 3.41 | 1.72 | .19^***^ | .07 | -.02 |  |  |  |  |  |  |  |  |  |  |
| 6. Condition  (WRE vs. Controls) | .30 | .46 | -.03 | .02 | .12^**^ | -.05 |  |  |  |  |  |  |  |  |  |
| 7. Feeling to Ingroup | 68.55 | 20.07 | .20^***^ | .02 | .06 | .36^***^ | .03 |  |  |  |  |  |  |  |  |
| 8. Feeling to Outgroup | 64.00 | 18.44 | .09^**^ | -.04 | .05 | -.13^***^ | .09^*^ | .43^***^ |  |  |  |  |  |  |  |
| 9. Feeling to U.S.Gov | 40.46 | 26.87 | .15^***^ | .04 | .09^*^ | .58^***^ | .01 | .56^***^ | .04 |  |  |  |  |  |  |
| 10. Feeling to PRC Gov | 20.13 | 20.96 | -.01 | -.01 | .11^**^ | .01 | .08^*^ | .07 | .27^***^ | .22^***^ |  |  |  |  |  |

*Note*. Pearson’s correlations; all tests two-sided.

^***^ *p* < .001, ^**^ *p* < .01, and ^*^ *p* < .05.

# Additional Details of Study Method and Analyses

For transparency, we have reported rules for determining sample size and any data exclusions below. All studies were advertised to participants as a survey concerning their opinions about social issues in general to minimize self-selection bias. Less than 2.6% of participants dropped out in Studies 1 to 5. Study 6 (which used a writing task) yielded a higher attrition rate of 14% (USA) and 10% (UK) respectively. When possible, we conducted attrition analyses (using univariate tests of variance) which indicated no significant differences between the participants who completed the survey and participants who dropped out (*p*’s > .095), with two exceptions which are noted in Study 5. To facilitate scientific replication, the sensitivity power analysis of each study is reported below. It indicates the minimum effect size the study affords the focal analysis (with the given sample size, with 80% power, and at the conventional significance level of .05; Faul, Erdfelder, Buchner, & Lang, 2009). In all studies, continuous predictor variables were mean-centered.

## Study 1

The Hong Kong Umbrella Movement started in September 2014. The protesters occupied major financial districts and streets in the region, drawing international attention ^3^. During this conflict, we examined if wise reasoning was relevant to people’s intergroup perceptions, examining views of both protesters and non-protesters toward protesters as a target group that was highlighted in the news at the time. Intergroup bias in this context was operationalized as more polarized attitudes toward protesters, between protester versus non-protester respondents.

**Participants, Procedure, and Power.** We recruited undergraduate students through a Hong Kong University research participant pool in late November 2014. All respondents are fluent in English as English is the medium of instruction. We did not know how long the conflict would persist; thus, we aimed to recruit as many participants as possible before the end of the term. This resulted in a sample of 75 undergraduates. Among them, 42 identified as protesters, and 33 identified as non-protesters. One participant had missing data on key measures and was excluded from analyses, resulting in a final sample size of 74. This sample size gives us 80% power to detect a medium-to-large minimum effect size of an interaction between wise reasoning and group membership (i.e., η^2^_p_ = .10). Our subsequent studies examine the hypothesis using larger samples. In this study, participants were asked to form an impression of the Umbrella Movement protesters. They then reported their attitudes toward the protesters. Then we assessed participants’ wise reasoning while thinking about the intergroup conflict and reporting their perceptions toward the protesters. No personally identifying information was collected.

**Measures.**

***Intergroup reflection.*** Participants formed impressions through visual stimulus ^4^; specifically they viewed a slideshow of 20 masked headshots of Umbrella Movement protesters. Among the headshots, the number of male and female protesters was equal. The presentation order was randomized to minimize any gender or order effects.

***Intergroup bias.*** Participants reported their perceptions toward the protesters in two measures. They reported the extent to which they perceived the protesters as warm (i.e., “warm”, “friendly”, “good-natured”; Cronbach’s *α* = .96) and as trustworthy (i.e., “trustworthy”, “honest”, and “sincere”; *α* = .90) on a scale from 1 = *Not at all* to 7 = *Extremely*. We measured perceptions of warmth and trustworthiness because they are basic perceptions about groups and critical drivers of intergroup behaviors ^5^. Higher ratings of warmth and trustworthiness indicated stronger positivity toward protesters.

***Wise reasoning.*** A short 12-item version of the Situated Wise Reasoning Scale was used. Participants responded to the items on a scale from 1 = *Not at all* to 5 = *Very much* (see both the brief and full wise reasoning measures on p.3 in SI). Cronbach’s alpha was high, *α* = .91, suggesting that the items converge into one coherent construct, as in past research.^[[1]](#footnote-1)^

**Detailed Analysis.** Intergroup bias in warmth and trust perceptions were analyzed with multiple regressions, using group membership, wise reasoning, and their interaction as the predictors in the model. We hypothesized that wise reasoning would be associated with weaker intergroup bias. Specifically, we expected that wise reasoning would relate to stronger positivity toward the protesters among non-protesters, and that there would be less attitude polarization between protester and non-protester participants’ positivity among those with strong (vs. weak) wise reasoning.

As predicted, there were significant Group × Wise Reasoning interactions on perceptions of warmth and trust, *B*_warmth_ = 1.25, *SE* = .53, *t*(70) = 2.36, *p* = .021, η^2^*_p_* = .07, 95% CI[.19, 2.31], *B*_trust_ = 1.16, *SE* = .40, *t*(70) = 2.88, *p* = .005, η^2^*_p_* = .11, 95% CI[.36, 1.96]. This indicated that positivity toward protesters depended on not only group membership but also wise reasoning (see Figure 1). Among non-protester participants, wise reasoning was related to stronger positivity toward the outgroup (protesters), *B*_warmth_ = 1.25, *SE* = .33, *t*(31) = 3.83, *p* = .001, η^2^*_p_* = .32, 95% CI[.58, 1.92], *B*_trust_ = 1.12, *SE* = .25, *t*(31) = 4.52, *p* < .001, η^2^*_p_* = .40, 95% CI[.61, 1.62]. Wise reasoning did not affect protester participants’ positivity toward their ingroup (protesters), *p’*s > .250.

Next, we examined whether wise reasoning related to less between-group polarization. Specifically, we looked at group differences (between protester and non-protester participants) in positivity toward protesters at the levels of weak (-1 SD) and strong (+1 SD) wise reasoning. Among those who had weaker wise reasoning (-1SD), there was a significant group difference: non-protester participants (vs. protester participants) showed less positivity toward protesters, *B*_warmth_ = -1.00, *SE* = .41, *t*(70) = -2.43, *p* =.018, η^2^*_p_* = .08, 95% CI[-1.83, -.18], *B*_trust_ = -.91, *SE* = .31, *t*(70) = -2.91, *p* = .005, η^2^*_p_* = .11, 95% CI[-1.53, -.29]. In contrast, among those who had stronger wise reasoning (+1SD), there was no significant group difference in positivity toward protesters, *B*_warmth_ = .39, *SE* = .41, *t*(70) < 1, *p* > .250, η^2^*_p_* = .01, 95% CI[-.43, 1.21], *B*_trust_ = .38, *SE* = .31, *t*(70) = 1.21, *p* = .231, η^2^*_p_* = .02, 95% CI[-.25, 1.00]. Additional analyses showed that these results were not moderated by participant age, *p*’s > .061, or gender, *p*’s > .418.


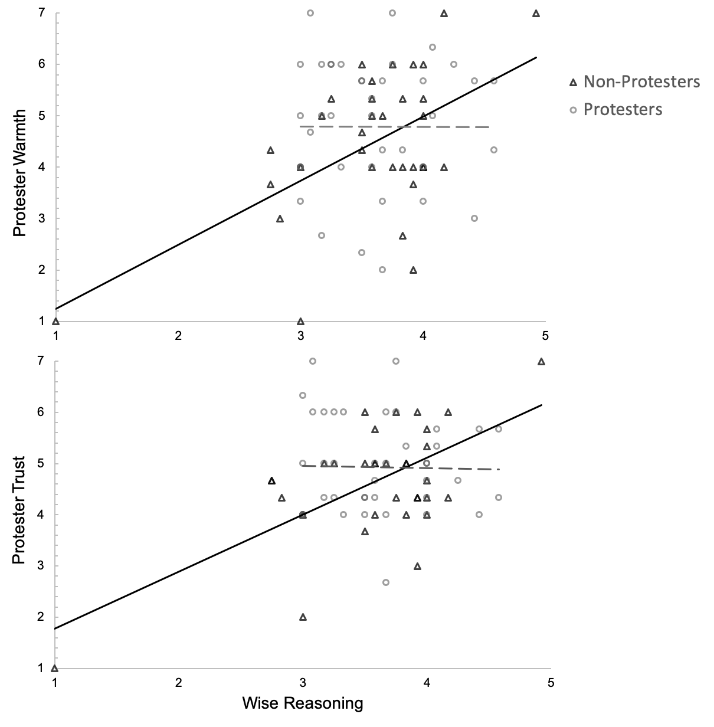

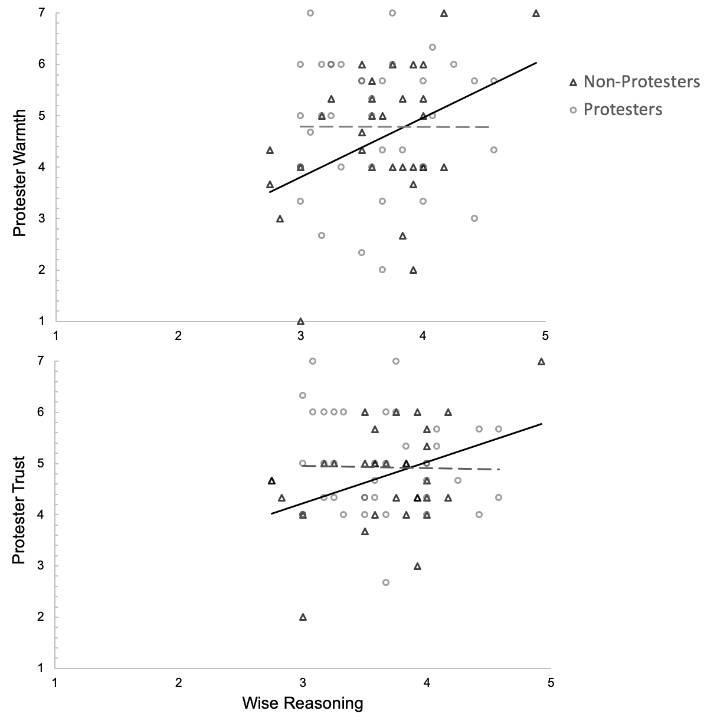


Panel A (N = 74) Panel B (N = 73)

*Supplementary* *Figure 1*. Results from Study 1: A panel of scatterplots of the attitudes toward target group as a function of participants’ ingroup versus outgroup membership and wise reasoning. Left panel illustrates the results of the full sample; Right panel illustrates the slopes when an outlier is excluded.

## Study 2

Study 2 aimed to replicate the effects of wise reasoning on intergroup bias found in Study 1 in a different intergroup context and with a larger sample (for a more powered test of the hypotheses). The study was situated in the context of the Baltimore, USA, protests following the death of a black man in police custody in 2015. Study 2 focused on attitudes toward police in a survey of Americans who identified with the protesters to varying degrees. Relatively few intergroup studies have examined attitudes toward majority and official status groups; it is an empirical question whether patterns observed between wise reasoning and bias would extend to attitudes toward an organized group such as police. Thus, this study was conducted to examine whether wise reasoning was associated with less attitude polarization, and to demonstrate the generalizability of wise reasoning effects in a less traditional type of intergroup context.

**Participants, Procedure, and Power.** With a target sample size of at least 300 participants in this study, we recruited 346 English-speaking U.S. residents via Mechanical Turk (MTurk). Nine participants had missing data on key measures and were not included in the analysis, resulting in a final sample of 337 participants. This larger sample size (compared to Study 1) gives us higher sensitivity: 80% power to detect a small minimum effect size of an interaction between wise reasoning and group membership (i.e., η^2^_p_ = .02; Faul et al., 2009). Participants first reported the extent to which they identified with the protesters. Then, they read a news clipping summarizing the Baltimore Protests and reported their positivity toward police as the dependent measure. Finally, we assessed their wise reasoning while reflecting on and answering questions about the conflict.

**Measures.**

***Identification with protesters.*** Participants reported the extent to which they identified with the Baltimore protesters, on a 7-point scale from 1 = *Very weakly identified* to 7 = *Very strongly identified*. There was broad variation in the identification of participants (*M* = 3.25, *SD* = 2.09). This continuous variable served as the indicator of participants’ group membership and was used as a predictor variable in the analysis.

***Intergroup reflection.*** We used an excerpt of an Associated Press news clipping that depicted events about the Baltimore protests. Participants were asked to reflect on their thoughts and feelings toward the event and the groups involved (as presented in SI under Intergroup Conflict Reflection Materials).

***Intergroup bias.*** Consistent with Study 1, participants reported their perceptions of warmth (*α* = .97) and trust toward police (*α* = .96) on a seven-point scale, from 1 = *Not at all* to 7 = *Extremely*. In addition, they also rated the extent to which they felt positive emotions (i.e. admiration, compassion, pride; *α* = .90) and negative emotions (i.e. disgust, contempt, anger; *α* = .88) toward the police on a seven-point scale, from 1 = *Not at all* to 7 = *Extremely*.

***Wise reasoning.*** We used the same wise reasoning measure as Study 1, except in this study participants completed the full 21-item version of the measure. Cronbach’s alpha was high, *α* = .94.

**Detailed Analysis**. Intergroup bias in warmth, trust, and positive and negative emotions were analyzed with multiple regressions, using group identification, wise reasoning, and their interaction as the predictors in the model. We hypothesized that wise reasoning would be associated with weaker intergroup bias. Specifically, we expected that among those who strongly identified with the Baltimore protesters, wise reasoning would be associated with relatively stronger positivity (and less negativity) toward police, and that there would be less polarization between strong and weak protest identifiers among those who had strong (vs. weak) wise reasoning.

***Warmth and trust perceptions.*** There were significant Group Identification × Wise Reasoning interactions on perceptions of warmth and trust, *B*_warmth_ *=* .20, *SE* = .05, *t*(333) = 4.08, *p <* .001, η^2^*_p_* = .05, 95% CI[.10, .30], *B*_trust_ = .20, *SE* = .05, *t*(333) = 4.06, *p <* .001, η^2^*_p_* = .05, 95% CI[.10, .30]. These interactions indicated that participants’ positivity toward police depended on not only their identification with protesters but also their wise reasoning (see Figure 2). Among strong protester-identifiers (+1SD), wise reasoning was associated with more positive perceptions toward police, *B*_warmth_ = .49, *SE* = .15, *t*(333) = 3.20, *p =* .002, η^2^*_p_* = .03, 95% CI[.19, .79], *B*_trust_ = .46, *SE* = .15, *t*(333) = 3.00, *p =* .003, η^2^*_p_* = .03, 95% CI[.16, .75]. These patterns were reversed for participants who identified weakly (-1SD) with the protesters, *B*_warmth_ = -.31, *SE* = .14, *t*(333) = -2.19, *p =* .029, η^2^*_p_* = .01, 95% CI[-.59, -.03], *B*_trust_ = -.33, *SE* = .14, *t*(333) = -2.38, *p =* .018, η^2^*_p_* = .02, 95% CI[-.61, -.06].

As in Study 1, we then examined whether wise reasoning related to less polarization. Among those who had weaker wise reasoning (-1SD), identification with protesters predicted strong polarization, *B*_warmth_ = -.49, *SE* = .06, *t*(333) = -8.26, *p* < .001, η^2^*_p_* = .17, 95% CI[-.60, -.37], *B*_trust_ = -.50, *SE* = .06, *t*(333) = -8.52, *p* < .001, η^2^*_p_* = .18, 95% CI[-.62, -.39]. However, among those who had stronger wise reasoning (+1SD), the influence of identification with protesters on positivity toward police was much attenuated, *B*_warmth_ = -.17, *SE* = .06, *t*(333) = -2.88, *p* = .004, η^2^*_p_* = .02, 95% CI[-.28, -.05], *B*_trust_ = -.18, *SE* = .06, *t*(333) = -3.16, *p =* .002, η^2^*_p_* = .03, 95% CI[-.30, -.07]. That is, intergroup perceptions between strong and weak protester-identifiers were less polarized among those who had stronger wise reasoning.

***Emotional reactions*.** Using the same analytic strategies as the above, we observed parallel effects of wise reasoning on participants’ emotions toward police. As predicted, there were significant Group Identification × Wise Reasoning interactions on positive and negative emotions toward police, *B*_positive emotions_ *=* .12, *SE* = .05, *t*(333) = 2.37, *p =* .019, η^2^*_p_* = .02, 95% CI[.02, .23], *B*_negative emotions_ *=* -.13, *SE* = .05, *t*(333) = -2.67, *p =* .008, η^2^*_p_* = .02, 95% CI[-.22, -.03]. Specifically, among strong protester-identifiers (+1SD), wise reasoning was associated with stronger positive emotions toward police, *B*_positive emotions_ = .51, *SE* = .16, *t*(333) *=* 3.15, *p* = .002, η^2^*_p_* = .03, 95% CI[.19, .83], and a marginal trend of less negative emotions toward police, *B*_negative emotions_ = -.25, *SE* = .15, *t*(333) = -1.72, *p* = .087, η^2^*_p_* = .01, 95% CI[-.54, .04] (see Figure 2). Among weak protester-identifiers (-1SD), wise reasoning was not associated with positive emotions, *p* > .250, and marginally associated with negative emotions toward the police, *B*_negative emotions_ = .25, *SE* = .13, *t*(333) = 1.84, *p* = .067, η^2^*_p_* = .01, 95% CI[-.02, .51]. Overall, the results supported the hypothesis that wise reasoning was related to stronger positivity toward the outgroup.

Wise reasoning was also associated with less polarization in emotional reactions. Among those who had weaker wise reasoning (-1SD), identification with protesters predicted more polarized emotional reactions, both positive and negative, toward the police, *B*_positive emotions_ = -.37, *SE* = .06, *t*(333) *=* -5.91, *p* < .001, η^2^*_p_* = .10, 95% CI[-.49, -.25], *B*_negative emotions_ = .60, *SE* = .06, *t*(333) = 10.58, *p* < .001, η^2^*_p_* = .25, 95% CI[.49, .71]. But among people with stronger wise reasoning (+1SD), polarization in emotional reactions was attenuated, *B*_positive emotions_ = -.17, *SE* = .06, *t*(333) *=* -2.80, *p* = .005, η^2^*_p_* = .02, 95% CI[-.30, -.05], *B*_negative emotions_ = .40, *SE* = .06, *t*(333) = 7.11, *p* < .001, η^2^*_p_* = .13, 95% CI[.29, .51]. Additional analyses showed that these results were not moderated by key participant demographic variables: age (*p*’s > .100), gender (*p*’s > .151), income (*p*’s > .209), education (*p*’s > .407), and race (white/Black; *p*’s > .213).

Noting the context of the Baltimore protests, the issue of race was an important consideration Study 2. Although in this study race was correlated with protester identification (Black participants were more likely than Non-Black participants to identify with protesters, *r* = .43), a number of Black participants (43.3%) did not identify strongly with protesters (i.e., at or below the midpoint of the scale, from 1 = *Very weakly identified* to 7 = *Very strongly identified*), and a number of white participants (21.6%) identified strongly with the protesters. Thus, respondents’ self-identification with protesters was deemed a good proxy for psychological group membership. Nonetheless, we also conducted analyses controlling for participant race, finding that it did not alter the pattern of effects significantly (*B*_warmth_ *=* .20, *SE* = .05, *t*(300) = 4.01, *p <* .001, η^2^*_p_* = .05, 95% CI[.10, .30], *B*_trust_ = .21, *SE* = .05, *t*(300) = 4.12, *p <* .001, η^2^*_p_* = .05, 95% CI[.11, .31], *B*_positive emotions_ *=* .13, *SE* = .05, *t*(300) = 2.35, *p =* .019, η^2^*_p_* = .02, 95% CI[.02, .23], *B*_negative emotions_ *=* -.15, *SE* = .05, *t*(300) = -3.33, *p =* .001, η^2^*_p_* = .04, 95% CI[-.24, -.06]). In addition, we did not find interactions between participant race (Black/white) and wise reasoning, *p*’s > .213, on the dependent variables, suggesting that the effect of wise reasoning on intergroup bias generalized across Black and white participants. Nonetheless, future research should investigate in more depth the mechanisms determining the relationships between group membership, race, and group identification in race-related conflicts, and the role that wise reasoning may play in these complex contexts.

Taken altogether, Study 2 replicated Study 1 results with a larger sample, showing further evidence that wise reasoning is associated with less intergroup bias.

## Study 3

Study 3 extended the previous findings in two major directions. First, it tested whether the effect of wise reasoning generalizes to a resource conflict between groups. Second, whereas Studies 1 and 2 examined the role of wise reasoning in reducing bias against a single focal group that was seen as either ingroup or outgroup, Study 3 used a within-subjects design and tested whether wise reasoning predicted people’s intergroup attitudes toward both ingroup and outgroup targets. In the spring of 2015, there were demonstrations in Hong Kong in response to the increasing number of Mainland Chinese visitors^6^. It was in this context that Study 3 examined whether and how wise reasoning would relate to intergroup attitudes among Hong Kong Chinese. We hypothesized that among Hong Kong Chinese, wise reasoning would be associated with stronger positivity toward the perceived outgroup targets (i.e., Mainland Chinese) and less polarized attitudes between the outgroup and ingroup targets (i.e., Hong Kong Chinese).

**Participants, Procedure, and Power**. We recruited Hong Kong Chinese undergraduates through a subject pool to participate in a survey for course credits. We aimed to recruit as many participants as possible during the controversy over the course of one academic term. In total, 298 students participated. This sample size gives us 80% power to detect a small minimum effect size of a mixed-level interaction between wise reasoning and target group (i.e., η^2^_p_ = .01; Faul et al., 2009). Participants first read a news clipping about some Hong Kong Chinese protesting against Mainland Chinese tourists. They then reported their attitudes toward Mainland Chinese and Hong Kong Chinese. Finally, we assessed their wise reasoning while reflecting on and answering questions about the conflict.

**Measures.**

***Intergroup reflection.*** We used a news clipping from CNN detailing the protests and conflicts between Hong Kong Chinese and Mainland Chinese tourists. Participants were asked to reflect on their thoughts and feelings toward the event and the groups involved.

***Intergroup Bias.*** Participants rated their feelings toward 1) Hong Kong Chinese and 2) Mainland Chinese, among other filler target groups, using feeling thermometers ^7^, from 0 = *Extremely Cold/Unfavorable* to 100 = *Extremely Warm/Favorable*. The presentation order of the target groups was randomized to prevent any potential order effect. A rating of 50 on this scale implies neutral feelings; ratings further to either pole on the feeling thermometer imply stronger positive or negative feelings. This feeling thermometer measure provides a holistic measure of participants’ general feelings toward the target groups. All participants were Hong Kong Chinese. Intergroup bias would manifest in less positive ratings toward the outgroup (Mainland Chinese) than toward the ingroup (Hong Kong Chinese) on the feeling thermometer.

***Wise reasoning.*** Consistent with Study 2, we used the full 21-item measure of wise reasoning (*α* = .95).

**Detailed Analysis.** Intergroup bias was analyzed using a mixed-model regression, entering feelings toward the ingroup (Hong Kong Chinese) and outgroup (Mainland Chinese) as within-subject outcomes and wise reasoning as the between-subject predictor. We hypothesized that wise reasoning would be associated with weaker intergroup bias. Specifically, we expected that wise reasoning would be associated with stronger positivity toward Mainland Chinese. Further, we expected that Hong Kong Chinese who had strong (vs. weak) wise reasoning would also show less polarization in positivity toward Mainland Chinese versus Hong Kong Chinese.

A significant interaction emerged between wise reasoning and positivity towards ingroup versus outgroup, *F*(1, 296) = 9.55, *t*(296) = 3.09, *p* = .002, η^2^*_p_* = .03. This indicated that participants’ feelings toward the ingroup and outgroup depended on their wise reasoning. Wise reasoning among Hong Kong Chinese participants significantly predicted stronger positivity toward the outgroup (Mainland Chinese), *B* = 7.85, *SE* = 1.77, *t*(296) = 4.44, *p* < .001, η^2^*_p_* = .06, 95% CI[4.36, 11.33], but did not influence their feelings toward ingroup (Hong Kong Chinese), *t*(296) < 1, *p* > .250 (Figure 3). Next, we examined whether wise reasoning was associated with less within-subject polarization. Among Hong Kong Chinese participants who had weaker wise reasoning (-1SD), feelings toward ingroup vs. outgroup targets differed substantially, *t*(296) = 14.75, *p* < .001, η^2^*_p_* = .42. However, among participants who had stronger wise reasoning (+1SD), intergroup polarization in positivity was attenuated, *t*(296) = 10.38, *p* < .001, η^2^*_p_* = .27. Participant age (*p* = .809) and gender (*p* = .057) did not moderate the above interaction effect. The potential gender effect was driven by a stronger relation between wise reasoning and feelings toward the outgroup (Mainland Chinese) among males, *t*(132) = 3.82, *p* < .001, η^2^_p_ = .10, than females, *t*(162) = 2.26, *p* = .025, η^2^_p_ = .03. Study 3 replicated and extended the findings that wise reasoning relates to weaker intergroup bias. Moreover, these effects hold for within-subject attitudes and generalize to a resource conflict between groups.

## Study 4

Studies 1 to 3 focused on the effects of wise reasoning on individuals’ attitudes toward a specific focal group in acute social conflicts. Given a relative paucity of research examining *both* groups, standing on what are presumably different sides of a conflict, Study 4 examined the effects of wise reasoning on intergroup bias in two focal groups within an ideological conflict. In early 2015, the Supreme Court of the United States (SCOTUS) considered legalizing same-sex marriage. Tensions arose quickly between parties supporting (e.g., LGBTQ communities, liberals) and opposing (e.g., Christians, conservatives) the legislation, resulting in petitions, protests, and occasions of intergroup hostility ^8,9^. On June 24, SCOTUS held a 5-4 decision requiring all states to grant same-sex marriage rights. It was in this context that we conducted Study 4 to examine whether wise reasoning was associated with less intergroup bias. We recruited adult Americans two days after the ruling for an online survey and measured their intergroup attitudes, religious and political affiliations, and wise reasoning. We hypothesized that wise reasoning would be associated with less intergroup bias among two groups of participants that historically hold apparently conflicting ideologies of marriage: Christians or conservative participants compared to LGBTQ or liberal participants ^10^. As in the studies above, we expected that wise reasoning would be associated with more outgroup positivity and less intergroup polarization.

**Participants, Procedure, and Power.** We recruited 306 English-speaking U.S. residents via MTurk.^^[[2]](#footnote-2)^^ Within this sample, we selected participants that could represent either side of the conflict on same-sex marriage in USA (as a function of religiosity and political conservatism)^10^. Participants who self-identified as Christian or Conservative (*n* = 144) and those who self-identified as LGBTQ or Liberal (*n* = 99) ^^[[3]](#footnote-3)^^. For parsimony in reporting, we will refer to the prior group as “Christian/Conservative” participants, and the latter group as “LGBTQ/Liberal” participants. This resulted in a final sample of 243 in the analysis. This sample size gives us 80% power to detect a small-to-medium minimum effect size of a mixed-level interaction between wise reasoning and target group membership (i.e., η^2^_p_ = .03)^2^. Participants first read a news clipping about the same-sex marriage ruling. Then, they reported their feelings to their ingroup and outgroup on a feeling thermometer. Next, participants reported their support for same-sex marriage and their motivation to meet and interact with outgroup members. Finally, we assessed participants’ wise reasoning while reflecting on and answering questions about the conflict.

**Measures.**

***Intergroup reflection.*** We used a news clipping from the New York Times reporting the SCOTUS same-sex marriage equality ruling. Participants were asked to reflect on their thoughts and feelings toward the event and the groups involved.

***Intergroup bias.*** We assessed attitudes toward “Christians” and “Gays” among other filler group targets using feeling thermometers (Gervais et al., 2011), from 0 = *Extremely Cold/Unfavorable* to 100 = *Extremely Warm/Favorable*. The target term “Gays” was selected when this study was conducted, in mid-2015, based on precedent from the literature at the time.^11,12^ The order of the target groups was randomized to prevent any potential order effect.

***Wise reasoning.*** Participants completed the standard 21-item measure of wise reasoning (*α* = .89).

**Detailed analysis.** Intergroup bias in feeling thermometer ratings was analyzed using a mixed-model regression: feelings toward “Christians” and “Gays” were entered as the within-subject outcomes, and group membership and wise reasoning were entered as between-subject predictors. We hypothesized that wise reasoning would be associated with weaker intergroup bias. Specifically, we predicted that wise reasoning in Christian/Conservative participants would be associated with stronger positivity toward gays, whereas wise reasoning in LGBTQ/Liberal participants would be associated with stronger positivity toward Christians. Further, we expected that participants’ within-subject intergroup positivity toward both target groups would be less polarized among those who had strong (vs. weak) wise reasoning.

There was a significant mixed-level 3-way interaction, *F*(1, 238)= 5.49, *p =* .020, η^2^*_p_* = .02, suggesting that positivity toward the target groups depended on both participants’ group membership and their wise reasoning. First, we tested if wise reasoning was associated with stronger outgroup positivity. Among the Christian/Conservative participants, wise reasoning predicted stronger positivity toward Gays, *B* = 7.61, *SE* = 3.62, *t*(141) *=* 2.10, *p =* .037, η^2^*_p_* = .03, 95% CI[.45, 14.77], but did not significantly predict positivity toward Christians (*p* > .250). Among the LGBTQ/Liberal participants, wise reasoning predicted stronger positivity toward Christians, *B* = 6.44, *SE* = 2.42, *t*(97) *=* 2.66, *p =* .009, η^2^*_p_* = .07, 95% CI[1.64, 11.24], but did not significantly predict positivity toward Gays (*p* > .250).

Second, we examined the effect of wise reasoning on within-subject intergroup polarization (i.e., the difference between positivity towards Christians and Gays). Among the Christian/Conservative participants with weaker wise reasoning (-1 SD), there was a significant difference in positivity toward in- vs. outgroup, *t*(142) = -2.72, *p* = .007, η^2^*_p_* = .05. However, among the Christian/Conservative participants who had stronger wise reasoning (+1 SD), there was no intergroup polarization in positivity (*p* > .250). The same analyses were conducted among the LGBTQ/Liberal participants: among those who had weaker wise reasoning (-1 SD), there was extreme difference in positivity toward in- vs. outgroup, *t*(97) *= -*14.39, *p* < .001, η^2^*_p_* = .68; but among those who had stronger wise reasoning (+1 SD), intergroup polarization in positivity was attenuated, *t*(97) = -10.09, *p* < .001, η^2^*_p_* = .51. Participants’ age (*p* = .931), gender (*p* = .129), education (*p* = .170), and race (white/Others; *p* = .259), did not moderate the above interaction effect.

In sum, Study 4 revealed additional support for the hypothesis that wise reasoning is associated with reduced intergroup bias during a historical intergroup context where the same-sex marriage issue was a focal point of contention in a nation. The results showed that Christian/Conservative participants’ and LGBTQ/Liberal participants’ wise reasoning predicted stronger positivity toward the outgroup, but it did not predict positivity toward their ingroup. Their wise reasoning was also related to less intergroup attitude polarization.

## Study 5

Study 5 had two major objectives. First, it aimed to replicate the effects of wise reasoning on intergroup bias in two focal groups within a chronic ideological conflict (as in Study 4). Second, it explored whether wise reasoning has incremental validity above and beyond other psychological factors that can contribute to intergroup bias. The study measured common predictors of intergroup bias found in the literature in addition to wise reasoning. These predictors included trait perspective taking, empathy, and need for cognitive closure ^13–15^. As robustness analyses, we first tested the effects of wise reasoning controlling for these factors both individually and all together. We also contrasted their independent effects on bias with those of wise reasoning (e.g., similarly weaker bias for both the majority and minority group members). This test is critical as it directly addresses whether wise reasoning adds unique and novel contribution to the literature on intergroup bias reduction. We discuss our rationale for comparing the effects of wise reasoning with these variables below.

Previous research suggests that although such factors as perspective taking, empathy, and need for cognitive closure can influence intergroup attitudes, their effects are not always consistent across different conflict situations ^16,17^. For example, research suggests that perspective taking can backfire in intergroup settings. Specifically, perspective taking is often built off egocentric bias ^18^ and can boost egocentric concerns ^19^. Thus, it may increase intergroup bias for certain people and groups, such as those with lower status and lower self-esteem; it may also increase bias in situations where the self or self-threat is salient, such as highly competitive or aggressive situations ^20,21^. Similarly, research suggests that empathy can also backfire, leading to intergroup bias by amplifying ingroup positivity alone, thus exacerbating intergroup attitude polarization ^22^. Wise reasoning has its foundation in ego-decentering (vs. egocentrism)^23^ and has been shown to sustain cooperative attitudes and behavior, even in a self-threatening situation ^23^. Thus, we compared the effects of wise reasoning on balancing intergroup attitudes (stronger positivity toward outgroups, for both majority and minority group members) with those of perspective taking and empathy in the current study.

Finally, we also compare wise reasoning and need for cognitive closure (i.e., desire for a firm answer and aversion to ambiguity; ^24^. High need for closure motivates people to settle on predictable, easily accessible, and firm knowledge about the world ^25^, and has been linked to stronger intergroup bias ^25–27^. Although low need for closure and wise reasoning share some commonality in terms of whether people accept uncertainty and change, we argue that wise reasoning involves more integrative thinking processes that go beyond a low need to attain certainty. Theoretically, unlike low need for closure, wise reasoning constitutes an integrative process that does not only accommodate uncertainty and change, but it also considers the big picture in order to integrate different viewpoints, goals, and needs to resolve a situation ^28^. Empirically, however, the degree to which the two constructs have a similar or differentiated effect remains an open question. Thus, we also tested whether wise reasoning had unique predictive validity above and beyond need for closure.

We conducted the study in the context of the same-sex marriage debate in Canada. Although same-sex marriage has been legal in Canada since 2005, tensions were again heightened between the LGBTQ and Christian communities because of the Supreme Court ruling in the neighboring U.S., in addition to several ongoing lawsuits concerning refusal of services to same-sex couples by Christian merchants ^29–31^. We hypothesized that wise reasoning would be associated with less intergroup bias for both Christian heterosexual participants and LGBTQ participants. Consistent with the previous studies, the effect would be manifested in stronger positivity toward the outgroup and less polarization in participants’ feelings toward in- vs. outgroup.

**Participants, Procedure, and Power.** To avoid self-selection bias, we planned to preselect LGBTQ participants and Christian heterosexual participants from a psychology participant pool at a large Canadian University. Only responses from participants who self-identified as lesbian, gay, or bisexual were available for selection. Participants completed an online survey in return for course credit. We aimed to recruit as many participants as possible over the course of two semesters, and 308 participated in total (166 Christian heterosexual participants; 142 lesbian, gay, or bisexual participants). Among the lesbian, gay, or bisexual participants, 35% self-identified with a religion (25% Christian, 3.5% Muslim, 3.5% Hindu, 2.1% Buddhist, 1.4% Jewish). This sample size yields 80% power to detect a small minimum effect size of a mixed-level interaction between wise reasoning and target group membership (i.e., η^2^_p_ = .01; Faul et al., 2009). Some lesbian, gay, or bisexual participants identified with a religion. Excluding these participants or not did not alter the pattern or significance of the results. To maximize the sample size, they were retained in the analyses.^^[[4]](#footnote-4)^^ Attrition analyses (univariate tests of variance) indicated that more lesbian, gay, or bisexual participants did not complete all study measures (*p* = .002) and that those who did not complete all study measures were lower in one of the control variables (need for closure; *p* = .035). Participants first read a news clipping summarizing the same-sex marriage debate. Afterward, participants reported their feelings toward the two groups and their support for same-sex marriage. Finally, we assessed their wise reasoning while reflecting on and answering questions about the conflict.

**Measures.**

***Intergroup reflection.*** We compiled a summary news clipping about the same-sex marriage debate from a major Canadian news outlet (The Globe and Mail) with information about same-sex marriage legislation and religious freedom. The news clipping presented arguments for both sides of the debate. Participants were asked to reflect on their thoughts and feelings toward the event and the groups involved.

***Intergroup bias.*** Participants reported their feelings toward “Homosexuals” and “Christians” among other filler group targets using feeling thermometers. Unlike Study 4, we used the term “Homosexuals” instead of “Gays” in this study because past research indicated that people’s attitudes might differ depending on the terms used to describe sexual minority groups ^32,33^. Using different terms across studies allow us to explore generalizability of wise reasoning’s correlations across term usage. The presentation order of the target groups was randomized to prevent any potential order effect.

***Wise reasoning.*** Participants completed the full 21-item measure of wise reasoning (*α* = .94).

***Control variables.*** To contrast and test the unique predictive power of wise reasoning, we assessed three common predictors of intergroup bias as control variables. We used two subscales from Davis’ Interpersonal Reactivity Index ^34^. We included the seven items assessing *perspective taking*, the tendency to adopt other people’s point of view (e.g., “I sometimes try to understand my friends better by imaging how things look from their perspective.”; *α* = .80) and the seven items assessing *empathic concern*, the tendency to experience feelings to other people undergoing negative experiences (e.g., “When I see someone being taken advantage of, I feel kind of protective towards them.”; *α* = .81). Participants responded to items on a 5-point scale, from 1 = *Does not describe me well*, to 5 = *Describes me very well*. Participants also responded to items from Roets and Van Hiel's abbreviated version of the *need for closure* scale, measuring the tendency to seize and hold on to any answer to avoid uncertainty.^14^ Participants responded to 15 items on a 6-point scale, from 1 = *Strongly disagree*, to 6 = *Strongly agree* (e.g., “I do not usually consult many different opinions before forming my own view.”; *α* = .81).

**Detailed Analysis.** Intergroup bias in feeling thermometer ratings was analyzed using a mixed-model regression, entering feelings toward “Christians” and “homosexuals” as the within-subject outcomes, and group membership and wise reasoning as between-subject predictors. We hypothesized that wise reasoning would be associated with less intergroup bias for both Christian heterosexual participants and lesbian, gay, or bisexual participants. Specifically, we predicted that wise reasoning in Christian heterosexual participants would be associated with stronger positivity toward homosexuals, whereas wise reasoning in lesbian, gay, or bisexual participants would be associated with stronger positivity toward Christians. Furthermore, we expected that participants’ within-subject intergroup positivity toward ingroup and outgroup would be less polarized among those who had strong (vs. weak) wise reasoning.

A significant mixed-level 3-way interaction emerged, *F*(1, 298)= 8.50, *t*(298) = 2.92, *p =* .004, η^2^*_p_* = .03, suggesting that feelings toward the target groups depended on both participants’ group membership and wise reasoning. First, we tested if wise reasoning was associated with stronger positivity toward the outgroups. Among Christian heterosexual participants, wise reasoning predicted stronger positivity toward the outgroup (homosexuals), *B* = 7.54 *SE* = 3.03, *t*(161) *=* 2.49, *p =* .014, η^2^*_p_* = .04, 95% CI[1.57, 13.52], but did not significantly predict feelings toward the ingroup (Christians), *t*(161) < 1, *p* > .250. Among lesbian, gay, or bisexual participants, wise reasoning predicted stronger positivity toward the outgroup, *B* = 9.29, *SE* = 3.40, *t*(137) *=* 2.73, *p =* .007, η^2^*_p_* = .05, 95% CI[2.56, 16.01], but did not significantly predict feelings toward the ingroup, *t*(137) *<* 1, *p* > .250.

Next, we examined the effect of wise reasoning on within-subject intergroup polarization of feelings. Among Christian heterosexual participants who had weaker wise reasoning (-1 SD), there was a significant difference in positivity toward in- vs. outgroup, *t*(161) = 2.75, *p* = .007, η^2^*_p_* = .05. In contrast, among Christian heterosexual participants who had stronger wise reasoning (+1 SD), there was no intergroup polarization in positivity, *t*(161) *<* 1, *p* > .250. The same analyses were conducted among lesbian, gay, or bisexual participants: among those who had weaker wise reasoning (-1 SD), there was a significant difference in positivity toward in- vs. outgroup, *t*(137) *=* 7.84, *p* < .001, η^2^*_p_* = .31; but among those who had stronger wise reasoning (+1 SD), attitude polarization was much attenuated, *t*(137) = 3.86, *p* < .001, η^2^*_p_* = .10. Participant age (*p* = .644) and gender (*p* = .147) did not moderate the above interaction effect.

**Robustness Analyses.** We conducted contrast and robustness analyses to test whether wise reasoning has unique predictive validity over some common individual differences that have previously been found to associate with intergroup bias, namely, perspective taking, empathic concern, and need for closure. All statistics are summarized in Table S4 below.

As initial simple tests of incremental predictive validity, we re-examined the focal mixed-level 3-way interaction (as in the above section) by also entering the main effects of the covariates as control variables in the model, first individually and then all three simultaneously. Controlling for the main effects of perspective taking (Model 2), empathic concern (Model 3), and need for closure (Model 4) separately in the model did not influence the wise reasoning effect: the interaction effect between wise reasoning, group membership and target group, remained significant. Likewise, controlling for all three covariates simultaneously (Model 5) did not affect the pattern of the main result. Further, we then moved on to re-examine the mixed-level 3-way interaction with a more complex model. In additional to the original model with the wise reasoning interaction terms, we entered the parallel 3-way interaction term (i.e., target group × group membership × covariate) and its corresponding lower-order interactions and main effects into the model. Controlling for the interaction effects of perspective taking (Model 6), empathic concern (Model 7), and need for closure (Model 8) separately in the model did not influence the significance of the wise reasoning interaction effect. Similarly, controlling for all three interaction effects of the covariates also did not alter the wise reasoning interaction effect (Model 9).

Next, we conducted a more in-depth exploration: on the notion that wise reasoning is uniquely and more reliably related to weaker intergroup bias, we examined the moderating effects of perspective taking, empathic concern, and need for closure (independently) on intergroup attitudes, for both Christian heterosexual and lesbian, gay, or bisexual participants. We conducted mixed-model regressions for each of the three covariates, testing for three-way interactions that would indicate moderation of the effect of group membership and target group on intergroup attitudes, as with our focal tests of wise reasoning (e.g., Perspective taking × Group Membership × Target Group).

First we looked at perspective taking, finding no three-way interaction, *F*(1, 298) < .01, *p >* .250. Examining the effect of perspective taking for Christian heterosexual and lesbian, gay, or bisexual participants separately, we found that perspective taking was not related to balanced attitudes across groups. Specifically, in Christian heterosexual participants, perspective taking predicted stronger positivity toward the outgroup, *t*(161) = 2.01, *p* = .047, and not the ingroup, *t*(161) < 1, *p* > .250. However, consistent with recent speculation ^18^, in lesbian, gay, or bisexual participants, perspective taking did not predict stronger positivity toward the outgroup, *t*(137) < 1, *p* > .250; rather, for lesbian, gay, or bisexual participants, perspective taking predicted stronger positivity only to the ingroup, *t*(137) = 3.47, *p* = .001. In short, perspective taking seems to be more effective in promoting positivity toward the ingroup and not the outgroup for minority groups, consistent with prior research ^17^. Examining empathic concern, again we found no three-way interaction, *F*(1, 298) = .01, *p >* .250. As with perspective taking, for Christian heterosexual participants, empathic concern predicted stronger positivity toward both the ingroup, *t*(161) = 2.10, *p* = .038, and the outgroup, *t*(161) = 3.08, *p* = .002. Yet, again for minority lesbian, gay, or bisexual participants, empathic concern predicted stronger positivity only toward the ingroup, *t*(137) = 2.01, *p* = .047, and not the outgroup, *t*(137) < 1, *p* > .250. Finally, looking at need for closure, there was no three-way interaction, *F*(1, 298) = .33, *p >* .250. In this case, need for closure did not predict positivity to either target group for Christian heterosexual participants, *ts*(161) < 1, *p’s* > .250. For lesbian, gay, or bisexual participants, need for closure was unrelated to positivity toward the outgroup, *t*(137) < 1, *p* > .250. There was an non-significant trend predicting less positivity toward the ingroup, *t*(137) = -1.68, *p* = .094.

**Supplementary Factor Analysis.** Given the conceptual similarity between wise reasoning and additional variables assessed in Study 4 – perspective taking, empathic concern, and need for closure – we also tested their psychometric overlap with wise reasoning with three separate factor analyses. All analyses used Principal Axis Factoring and Direct Oblimin Rotation. The first analysis included all wise reasoning items and the seven perspective taking items from Davis’ (1983) scale. The second analysis included all wise reasoning items and the seven empathic concern items from Davis’ (1983) scale. The third analysis included all wise reasoning items and the 15 need for closure items from Roets and Van Hiel's (2011) scale.

As seen in Table S5, in each analysis, items from the additional scales did not cluster with wise reasoning items, and wise reasoning items clustered strongly and reliably with their theorized dimensions. One of the assumptions of Principal Axis Factoring is that there are underlying psychological or causal structures influencing similarities in responses to different factors of items ^35^. Thus, these findings provide some evidence that despite some conceptual similarity between wise reasoning and these additional variables, wise reasoning indeed represents a unique psychological variable. However, the same caveat we raise concerning our control tests in Study 5 also pertains to these analyses: The unique clustering of items in these factor analyses may represent differences in situational versus global psychological processing or assessment, rather than differences in constructs *per se*. Thus, we remain cautious in our interpretation of the current factor analytic results.

Together, results from the robustness analyses suggested that wise reasoning has incremental predictive validity over perspective taking, empathy, and need for closure. Moreover, it demonstrated uniquely consistent validity for predicting weaker intergroup bias, whereas the other psychological variables showed differential validity—not consistently relating to stronger positivity toward outgroups for both majority and minority groups in a conflict, and not therefore relating to more balanced, less polarized intergroup feelings for both groups.

### Supplementary Table 4. Study 5 Results of Mixed-level 3-way Interactions.

*Group Membership (Christian heterosexual participants / lesbian, gay, or bisexual participants; between-subjects) × Feelings toward Target Groups (“Christians” / “Homosexuals”; within-subjects) × Wise Reasoning, with and without Covariates.*

|  | **Model 1** | | | **Model 2** | | | **Model 3** | | | **Model 4** | | | **Model 5** | | | **Model 6** | | | **Model 7** | | | **Model 8** | | | **Model 9** | | |
| --- | --- | --- | --- | --- | --- | --- | --- | --- | --- | --- | --- | --- | --- | --- | --- | --- | --- | --- | --- | --- | --- | --- | --- | --- | --- | --- | --- |
| **Independent Variables** | *F_(p-_*_value_*_)_* | η^2^*_p_* | *F_(p-_*_value_*_)_* | | η^2^*_p_* | *F_(p-_*_value_*_)_* | | η^2^*_p_* | *F_(p-_*_value_*_)_* | | η^2^*_p_* | *F_(p-_*_value_*_)_* | | η^2^*_p_* | *F_(p-_*_value_*_)_* | | η^2^*_p_* | *F_(p-_*_value_*_)_* | | η^2^*_p_* | *F_(p-_*_value_*_)_* | | η^2^*_p_* | *F_(p-_*_value_*_)_* | | η^2^*_p_* |  |
| Between-Subject Effects |  |  |  | |  |  | |  |  | |  |  | |  |  | |  |  | |  |  | |  |  | |  |  |
| Intercept | 5137.63_(<.001)_ | .95 | 5142.70_(<.001)_ | | .26 | 5317.95_(<.001)_ | | .21 | 5099.79_(<.001)_ | | .38 | 5316.44_(<.001)_ | | .11 | 4821.56_(<.001)_ | | .95 | 5133.93_(<.001)_ | | .95 | 5120.44_(<.001)_ | | .95 | 5334.54_(<.001)_ | | .95 |  |
| Group Membership | 1.85_(.175)_ | .01 | 1.62_(.204)_ | | .01 | 1.08_(.300)_ | | .00 | 1.98_(.160)_ | | .01 | 1.15_(.285)_ | | < .01 | 1.54_(.216)_ | | .01 | .59_(.444)_ | | <.01 | 1.98_(.161)_ | | .01 | 1.06_(.303)_ | | < .01 |  |
| Wise Reasoning | 6.59_(.011)_ | .02 | 3.59_(.059)_ | | .01 | 2.92_(.088)_ | | .01 | 6.70_(.010)_ | | .02 | 2.53_(.113)_ | | .01 | 5.39_(.021)_ | | .01 | 3.34_(.069)_ | | .01 | 7.31_(.007)_ | | .02 | 2.89_(.901)_ | | .01 |  |
| *Perspective Taking* |  |  | 3.16_(.077)_ | | .01 |  | |  |  | |  | .13_(.722)_ | | < .01 | 2.59_(.109)_ | | .01 |  | |  |  | |  | .07_(.796)_ | | < .01 |  |
| *Empathy* |  |  |  | |  | 13.02_(<.001)_ | | .04 |  | |  | 10.08_(.002)_ | | .03 |  | |  | 11.80_(.001)_ | | .05 |  | |  | 12.05_(.001)_ | | .04 |  |
| *Need for Closure* |  |  |  | |  |  | |  | .84_(.360)_ | | < .01 | 1.14_(.287)_ | | < .01 |  | |  |  | |  | .70_(.403)_ | | <.01 | 1.37_(.243)_ | | .01 |  |
| *Perspective Taking*× Group Membership |  |  |  | |  |  | |  |  | |  |  | |  | 2.27_(.133)_ | | .01 |  | |  |  | |  | 4.54_(.034)_ | | .02 |  |
| *Empathy*× Group Membership |  |  |  | |  |  | |  |  | |  |  | |  |  | |  | 3.54_(.061)_ | | .01 |  | |  | 4.85_(.029)_ | | .02 |  |
| *Need for Closure* × Group Membership |  |  |  | |  |  | |  |  | |  |  | |  |  | |  |  | |  | 2.19_(.140)_ | | .01 | 1.51_(.220)_ | | .01 |  |
| Wise Reasoning × Group Membership | < .01_(.974)_ | < .01 | .07_(.787)_ | | < .01 | .05_(.487)_ | | < .01 | .01_(.944)_ | | < .01 | .08_(.776)_ | | < .01 | .05_(.817)_ | | <.01 | .39_(.533)_ | | <.01 | .06_(.0808)_ | | <. 01 | .01_(.929)_ | | < .01 |  |
|  |  |  |  | |  |  | |  |  | |  |  | |  |  | |  |  | |  |  | |  |  | |  |  |
| Within-Subject Effects |  |  |  | |  |  | |  |  | |  |  | |  |  | |  |  | |  |  | |  |  | |  |  |
| Target Group | 25.00_(<.001)_ | .08 | 26.069_(<.001)_ | | < .01 | 24.90_(<.001)_ | | < .01 | 24.61_(<.001)_ | | < .01 | 25.64_(<.001)_ | | .01 | 23.95_(<.001)_ | | .08 | 24.12_(<.001)_ | | .08 | 24.31_(<.001)_ | | .08 | 26.03_(<.001)_ | | .08 |  |
| Target Group × Group Membership | 60.31_(<.001)_ | .17 | 63.33_(<.001)_ | | .18 | 60.68_(<.001)_ | | .17 | 59.36_(<.001)_ | | .17 | 62.31_(<.001)_ | | .18 | 56.00_(<.001)_ | | .18 | 54.72_(<.001)_ | | .17 | 58.86_(<.001)_ | | .17 | 62.26_(<.001)_ | | .18 |  |
| Wise Reasoning × Target Group | .23_(.634)_ | < .01 | 1.69_(.194)_ | | .01 | .48_(.487)_ | | < .01 | .22_(.641)_ | | < .01 | 1.67_(.197)_ | | .01 | 1.50_(.222)_ | | .01 | .20_(.658)_ | | <.01 | .16_(.0690)_ | | < .01 | 1.44_(.231)_ | | .01 |  |
| *Perspective Taking* × Target Group |  |  |  | |  |  | |  |  | |  |  | |  | 7.43_(.007)_ | | .02 |  | |  |  | |  | 6.26_(.013)_ | | .02 |  |
| *Empathy* × Target Group |  |  |  | |  |  | |  |  | |  |  | |  |  | |  | 1.13_(.290)_ | | <.01 |  | |  | .01_(.932)_ | | < .01 |  |
| *Need for Closure* × Target Group |  |  |  | |  |  | |  |  | |  |  | |  |  | |  |  | |  | .00_(.998)_ | | < .01 | .04_(.846)_ | | < .01 |  |
| *Perspective Taking* × Target Group × Group Membership |  |  |  | |  |  | |  |  | |  |  | |  | .60_(.441)_ | | < .01 |  | |  |  | |  | .20_(.658)_ | | < .01 |  |
| *Empathy* × Target Group × Group Membership |  |  |  | |  |  | |  |  | |  |  | |  |  | |  | .32_(.574)_ | | <.01 |  | |  | .19_(.665)_ | | < .01 |  |
| *Need for Closure* × Target Group × Group Membership |  |  |  | |  |  | |  |  | |  |  | |  |  | |  |  | |  | .29_(.589)_ | | < .01 | .20_(.657)_ | | < .01 |  |
| Wise Reasoning × Target Group × Group Membership | 8.50_(.004)_ | .03 | 6.86_(.009)_ | | .02 | 8.18_(.005)_ | | .03 | 8.41_(.004)_ | | .03 | 6.74_(.010)_ | | .02 | 7.63_(.006)_ | | .02 | 8.94_(.003)_ | | .03 | 7.99_(.005)_ | | .03 | 7.28_(.007)_ | | .02 |  |

*Note*. Mixed-model regressions were conducted in each test; all tests two-sided. Control variables are italicized.

### Supplementary Table 5A. Factor Analysis of Wise Reasoning and Perspective Taking.

| **Wise reasoning and Perspective Taking Items** | | | | | | |
| --- | --- | --- | --- | --- | --- | --- |
| Item# | Factor | | | | | |
|  | 1 | 2 | 3 | 4 | 5 | 6 |
| WR3 | **.859** |  | .427 | .442 | .411 | -.333 |
| WR4 | **.851** |  | .396 | .351 | .434 | -.428 |
| WR1 | **.840** |  | .439 | .381 | .344 | -.345 |
| WR6 | **.772** |  | .322 | .509 | .476 | -.416 |
| WR5 | **.744** | .307 | .349 | .495 | .478 | -.430 |
| WR2 | **.714** |  | .469 | .379 | .427 |  |
| WR13 | **.685** |  | .351 | .410 | .386 | **-.604** |
| PT2 |  | **.780** |  |  |  |  |
| PT6 |  | **.673** |  |  |  |  |
| PT3 |  | **.633** |  |  |  |  |
| PT7 |  | **.626** |  |  |  |  |
| PT5 |  | **.622** |  |  |  |  |
| PT1 |  | .486 |  |  |  |  |
| PT4 |  | .353 |  |  |  |  |
| WR21 | .389 |  | **.875** |  | .464 |  |
| WR20 | .402 |  | **.842** |  | .460 |  |
| WR18 | .380 |  | **.769** |  | .483 | -.383 |
| WR19 | .338 |  | **.600** |  | .399 |  |
| WR7 | .478 |  | .301 | **.968** | .358 |  |
| WR8 | .413 |  |  | **.813** | .302 | -.326 |
| WR10 | .420 |  | .467 |  | **.770** | -.346 |
| WR11 | .371 |  | .456 |  | **.725** |  |
| WR9 | .356 |  | .406 | .317 | **.692** |  |
| WR12 | .414 |  | .428 |  | **.626** |  |
| WR17 | .488 |  | .464 | .352 | .364 | **-.782** |
| WR14 | **.612** |  | .345 | .414 | .455 | **-.677** |
| WR15 | **.619** |  | .391 | .482 | .346 | **-.638** |
| WR16 | .426 |  | .306 | .393 | .309 | **-.619** |

*Note*. Wise reasoning items are denoted with “WR” and correspond to item numbers on page 3 of this document. Perspective taking items are denoted with “PT”. For ease of parsing, only coefficients ≥ .6 are bolded.

### Supplementary Table 5B. Factor Analysis of Wise Reasoning and Empathic Concern

|  | | | | | | |
| --- | --- | --- | --- | --- | --- | --- |
| **Wise Reasoning and Empathic Concern Items** | | | | | | |
| Item# | Factor | | | | | |
|  | 1 | 2 | 3 | 4 | 5 | 6 |
| WR4 | **.861** |  |  | .335 | .443 | .589 |
| WR3 | **.854** |  |  | .403 | .454 | .537 |
| WR1 | **.842** |  | .314 | .358 | .367 | .535 |
| WR6 | **.775** |  |  | .481 | .500 | .586 |
| WR2 | **.744** |  | .362 | .384 | .426 | .437 |
| WR5 | **.742** |  |  | .477 | .501 | .596 |
| EC1 |  | **.744** |  |  |  |  |
| EC4 |  | **.731** |  |  |  |  |
| EC5 |  | **.719** |  |  |  |  |
| EC7 |  | **.689** |  |  |  |  |
| EC6 |  | **.660** |  |  |  |  |
| EC3 |  | **.630** |  |  |  |  |
| EC2 |  | .475 |  |  |  |  |
| WR21 | .423 |  | **.816** |  | .469 | .332 |
| WR20 | .427 |  | **.801** |  | .456 | .386 |
| WR18 | .412 |  | **.717** |  | .463 | .420 |
| WR19 | .354 |  | .573 | .303 | .387 |  |
| WR7 | .475 |  |  | **.919** | .356 | .443 |
| WR8 | .399 |  |  | **.855** |  | .444 |
| WR10 | .447 |  | .347 | .303 | **.756** | .409 |
| WR11 | .398 |  | .356 |  | **.724** |  |
| WR9 | .383 |  | .305 | .330 | **.666** | .342 |
| WR12 | .430 |  | .321 |  | **.658** | .317 |
| WR14 | .592 |  |  | .400 | .453 | **.789** |
| WR17 | .487 |  | .360 | .372 | .339 | **.776** |
| WR15 | .597 |  |  | .467 | .353 | **.766** |
| WR13 | **.661** |  |  | .377 | .407 | **.732** |
| WR16 | .428 |  |  | .397 |  | **.642** |

*Note*: Wise reasoning items are denoted with “WR” and correspond to item numbers on page 3 of this document. Empathic concern items are denoted with “EC”. For ease of parsing, only coefficients ≥ .6 are bolded.

### Supplementary Table 5C. Factor Analysis of Wise Reasoning and Need for Closure

|  | | | | | | | | |
| --- | --- | --- | --- | --- | --- | --- | --- | --- |
| **Wise reasoning and Need for Closure Items** | | | | | | | | |
| Item# | Factor | | | | | | | |
|  | 1 | 2 | 3 | 4 | 5 | 6 | 7 | 8 |
| WR3 | **.828** |  | .364 |  | -.427 |  | .588 | .359 |
| WR1 | **.827** |  | .390 |  | -.379 |  | .574 |  |
| WR4 | **.816** |  | .350 |  | -.355 |  | **.636** | .363 |
| WR6 | **.718** |  |  |  | -.499 |  | **.633** | .419 |
| WR2 | **.713** |  | .438 |  | -.411 |  | .484 | .332 |
| WR5 | **.679** |  | .301 |  | -.489 |  | **.640** | .423 |
| NC15 |  | **.761** |  |  |  | -.534 |  |  |
| NC6 |  | **.710** |  | .354 |  | -.453 |  |  |
| NC1 |  | **.627** |  |  |  | -.329 |  |  |
| NC4 |  | .586 |  |  |  | -.402 |  |  |
| NC11 |  | .536 |  | .427 |  |  |  |  |
| NC2 |  | .497 |  |  |  |  |  |  |
| NC10 |  | .483 |  | .334 |  |  |  |  |
| NC5 |  | .382 |  |  |  |  |  |  |
| WR20 | .320 |  | **.851** |  |  |  | .427 | .366 |
| WR21 | .304 |  | **.847** |  |  |  | .390 | .415 |
| WR18 |  |  | **.769** |  |  |  | .468 | .421 |
| WR19 |  |  | .578 |  |  |  | .336 | .355 |
| NC8 |  | .332 |  | **.739** |  |  |  |  |
| NC9 |  | .469 |  | **.730** |  |  |  |  |
| NC7 |  |  |  | .360 |  |  |  |  |
| NC14 |  |  |  | .301 |  |  |  |  |
| WR8 | .326 |  |  |  | **-.918** |  | .464 |  |
| WR7 | .380 |  |  |  | **-.852** |  | .498 |  |
| NC12 |  | .457 |  | .313 |  | **-.920** |  |  |
| NC13 |  | .489 |  |  |  | **-.874** |  |  |
| NC3 |  | .559 |  |  |  | **-.695** |  |  |
| WR14 | .474 |  | .310 |  | -.408 |  | **.813** | .400 |
| WR15 | .498 |  | .345 |  | -.469 |  | **.776** |  |
| WR17 | .377 |  | .478 |  | -.403 |  | **.772** |  |
| WR13 | .575 |  | .306 |  | -.389 |  | **.761** | .334 |
| WR16 | .346 |  | .332 |  | -.438 |  | **.640** |  |
| WR11 |  |  | .437 |  |  |  | .347 | **.705** |
| WR10 | .355 |  | .461 |  | -.330 |  | .447 | **.668** |
| WR9 |  |  | .397 |  | -.340 |  | .389 | **.628** |
| WR12 | .355 |  | .401 |  |  |  | .359 | **.616** |

*Note.* Wise reasoning items are denoted with “WR” and correspond to item numbers. Need for closure items are denoted with “NC”. For ease of parsing, only coefficients ≥ .6 are bolded.

## Full details of internal meta-analysis (Studies 1-5)

Consistent across Studies 1 to 5, wise reasoning predicted i) stronger outgroup positivity, and ii) less intergroup attitude polarization (see Table S6 for a summary of all mean differences). To estimate the true effect sizes of these results, we conducted two separate meta-analyses ^36^. In both analyses, we assumed heterogeneity across our diverse samples (i.e. random/mixed effects model). To avoid redundant sampling, we averaged the estimates of multiple dependent measures (e.g., warmth and trust) within each sample. Because there were no outgroup ratings (i.e. non-protester targets) for protester participants in Study 1, protester participants’ responses were not included in the meta-analysis. With the goal to summarize as many data points as possible, between-subjects effects (Studies 1 and 2) and within-subject effects (Studies 3 to 5) were aggregated. Studies 4 and 5 had two independent samples—Christian/Conservative participants and LGBTQ/Liberal participants (Study 4) and Christian heterosexual participants and LGB participants (Study 5), resulting in a total of 7 samples (i.e., *k* = 7; *N* = 1218) in the meta-analysis. In addition, we reverse-coded the coefficients of negative emotions (in Study 2) so that all coefficients represented results in the same (i.e., positive) direction.

The first meta-analysis calculated the weighted mean correlation (in Pearson’s *r*) between wise reasoning and outgroup positivity. Results showed that wise reasoning was significantly associated with stronger outgroup positivity, with a small-to-moderate average effect size, *r* = .21, *p* < .0001, 95% CI [.15, .27]. The percentage of total variability due to heterogeneity, *I*^2^ is 4.90%. Test of heterogeneity was not significant, *Q*(6) = 7.38, *p* = .287, suggesting that the relations between wise reasoning and outgroup positivity were reliable across the intergroup contexts studied. The second meta-analysis tested the moderating effect of wise reasoning on intergroup attitude polarization (i.e., positivity toward outgroup versus ingroup). It calculated the average of the weighted standard mean differences (Cohen’s d) of ratings between groups at the levels of +/-1SD wise reasoning. At the level of weak wise reasoning (-1SD), positivity toward the outgroup was significantly weaker than positivity toward the ingroup, *d* -1.24, *p* = .001, 95% CI [-1.99, -.49]. In contrast, at the level of strong wise reasoning (+1SD), intergroup positivity did not significantly differ, *d* = -.67, *p* = .080, 95% CI [-1.42, .08]. Test of heterogeneity between the two levels of wise reasoning was significant, *Q*_B_ = 13.59, *p* = .001, suggesting that stronger wise reasoning (+1SD) predicted less intergroup attitude polarization across the intergroup contexts studied.

### Supplementary Table 6. Means Differences in Intergroup bias in Studies 1 to 5.

| **Studies** |  | **Group Means** | | | |
| --- | --- | --- | --- | --- | --- |
| **Study 1** (*n*=74) | | Non-Protester  Participants | | Protester  Participants | |
|  | Positivity toward Protesters | Warmth | | | |
|  | Strong WR | **4.175^a^** | | 3.788^a^ | |
|  | Weak WR | **2.787^b^** | | 3.790^a^ | |
|  |  | Trust | | | |
|  | Strong WR | **5.279^a^** | | 4.903^a^ | |
|  | Weak WR | **4.041^b^** | | 4.951^a^ | |
| **Study 2** (*n*=337) | | Participants with Strong Protester Identification  (+1SD) | | Participants with Weak Protester Identification  (-1SD) | |
|  | Positivity toward Police | Warmth | | | |
|  | Strong WR | **3.050^c^** | | 3.716^b^ | |
|  | Weak WR | **2.276^d^** | | 4.206^a^ | |
|  |  |  | |  | |
|  |  | Trust | | | |
|  | Strong WR | **3.152^c^** | | 3.884^b^ | |
|  | Weak WR | **2.430^d^** | | 4.413^a^ | |
|  |  |  | |  | |
|  |  | Positive Emotions | | | |
|  | Strong WR | **2.946^b^** | | 3.632^a^ | |
|  | Weak WR | **2.140^c^** | | 3.599^a^ | |
|  |  |  | |  | |
|  |  | Negative Emotions | | | |
|  | Strong WR | **4.426^b^** | | 2.855^a^ | |
|  | Weak WR | **4.823^c^** | | 2.460^a^ | |
| **Study 3** (*n*=298) | | HK Chinese Participants | | | |
|  | Positivity toward: | Target Group: “Mainland Chinese” | | Target Group: “Hong Kong Chinese” | |
|  | Strong WR | **47.745^b^** | | 67.097^a^ | |
|  | Weak WR | **38.417^c^** | | 65.929^a^ | |
| **Study 4** (*n=*243) | | Christian or Conservative Participants | | | |
|  | Positivity toward: | Target Group: “Gays” | | Target Group: “Christians” | |
|  | Strong WR | **64.386^a^** | | 66.525^a^ | |
|  | Weak WR | **53.067^b^** | | 66.176^a^ | |
|  |  |  | |  | |
|  |  | Non-Religious Liberal or LGBTQ Participants | | | |
|  | Positivity toward: | Target Group: “Gays” | | Target Group: “Christians” | |
|  | Strong WR | 81.549^a^ | | **30.919^b^** | |
|  | Weak WR | 84.842^a^ | | **23.985^c^** | |
| **Study 5** (*n=*308) | | Christian Participants | | | |
|  | Positivity toward: | Target Group: “Homosexuals” | Target Group: “Christians | |  |
|  | Strong WR | **69.652^a^** | 70.352^a^ | |  |
|  | Weak WR | **59.800^b^** | 70.852^a^ | |  |
|  |  |  |  | |  |
|  |  | LGB Participants | | | |
|  | Positivity toward: | Target Group: “Homosexuals” | | Target Group: “Christians | |
|  | Strong WR | 77.472^a^ | | **57.733^b^** | |
|  | Weak WR | 79.748^a^ | | **45.605^c^** | |

*Note*. Linear regressions were used in Studies 1 and 2. Mixed-model regressions were used in Studies 3-5. All tests two-sided. WR=Wise reasoning. Ratings of outgroups are presented in bold. Comparisons of strong and weak wise reasoning are presented at the level of +/- 1 standard deviation respectively. For each measure, means that do not share the same superscript are significantly different at the level of *p* < .050.

As in previous studies ^1,37^, the current research focused on the overall effect of wise reasoning. We did not hypothesize differential effects of the various wise reasoning dimensions on intergroup bias. Nonetheless, we conducted exploratory analyses to examine whether certain dimension(s) of wise reasoning would exhibit particularly strong associations with intergroup bias. To maximize the reliability of estimates, we combined as much data as possible. In particular, we combined data from Studies 3, 4, and 5 as they all included a categorical group membership variable, the full wise reasoning scale, and feeling thermometer ratings of both ingroup and outgroup (*N* = 849; see Table S7). Results suggested that wise reasoning dimensions varied in their associations with positivity toward the ingroup and outgroup. All dimensions were related to more positivity toward the outgroup (*r*s: .09 to .22), with consideration of compromise and conflict resolution having the strongest positive association. Interestingly, although the dimensions of recognition of the limits of one’s knowledge and taking an outsider’s vantage point had the weakest associations with positivity toward the outgroup (*r*_limit_ = .09, *r*_outsider_ = .10), they were also associated with more moderate positivity toward the ingroup (*r*_limit_ = -.10, *r*_outsider_ = -.10). Taken together, wise reasoning as a whole is reliably associated with weaker intergroup bias and less polarization. The relationships between specific wise reasoning dimensions and aspects of intergroup bias are dynamic. They are consistent with past theories that wise reasoning is an integrative process that promotes a more balanced perspective in the face of challenges (e.g., Baltes & Staudinger, 2000; Brienza et al., 2017; Staudinger & Glück, 2011; Sternberg, 1998).

### Supplementary Table 7. Studies 3 to 5 Descriptives and Intercorrelations of Wise Reasoning Dimensions and Intergroup Bias.

|  |  |  | Correlations | | | | | |
| --- | --- | --- | --- | --- | --- | --- | --- | --- |
| **Variables** | Mean | SD | 1 | 2 | 3 | 4 | 5 | 6 |
| 1. Perspective Taking | 3.32 | .89 |  |  |  |  |  |  |
| 2. Change | 3.35 | .91 | .68^***^ |  |  |  |  |  |
| 3. Limits | 3.05 | .95 | .58^***^ | .58^***^ |  |  |  |  |
| 4. Compromise | 3.32 | .88 | .68^***^ | .69^***^ | .60^***^ |  |  |  |
| 5. Outsider viewpoint | 3.16 | .93 | .59^***^ | .57^***^ | .63^***^ | .60^***^ |  |  |
| 6. Outgroup Positivity | 49.49 | 26.57 | .21^***^ | .19^***^ | .09^*^ | .22^***^ | .10^**^ |  |
| 7. Ingroup Positivity | 71.16 | 22.30 | -.04 | -.04 | -.10^**^ | -.02 | -.10^**^ | -.13^***^ |

*Note*. Pearson’s correlations; two-sided tests. Wise reasoning dimensions: Perspectives - recognition and integration of others’ perspectives; Change - recognition of uncertainty and change; Limits - recognition of the limits of one’s own knowledge; Compromise - consideration of/search for compromise and conflict resolution; and Outsider viewpoint - application of an outsider’s vantage point. Ingroup/Outgroup positivity are feeling thermometer scores.

^***^ *p* < .001, ^**^ *p* < .01, and ^*^ *p* < .05.

## Study 6

Study 6 tested the utility of a brief online wise reasoning exercise for prompting wise reasoning experimentally with three objectives: i) to provide initial evidence of causality, ii) to explore the possibility of developing training and education on wise reasoning in the future, and iii) to examine the mechanism–potential downstream effects of wise reasoning via its effect on attitude polarization. We measured all participants’ feelings toward immigrants, their behavioral intentions (e.g., endorsement of hostile policies), and actual behaviors (e.g., donations to a charity that serves immigrants, subscribing to volunteer opportunities to help immigrants). These new behavioral outcomes measured helping actions that contribute to collaborative intergroup relations. We tested whether the experimental condition of wise reasoning, as compared to a control condition, would increase wise reasoning, and whether it would influence intentions and actions to help immigrants directly, or indirectly through reduced attitude polarization. Results from the behavioral outcomes extend our understanding of wise reasoning and reveal whether manipulating wise reasoning can lead to outcomes that go beyond perceptions, feelings and attitudes.

Since 2016, immigration issues were discussed in North America and Europe accentuating the intergroup boundary between nationals and (residing and prospective) immigrants ^40–45^. In summer 2018, we conducted Study 6 to examine intergroup bias in the context of the USA and UK immigration disputes. Study 6 recruited home-country nationals in the USA and the UK to complete an online survey. We randomly assigned participants to either the experimental or the control condition. In the Wise Reasoning Experimental (WRE) Condition, participants responded to reflection questions to prompt wise reasoning. Participants in the Control Condition did not receive any reflection questions.

**Participants, Procedure, and Power.** This experiment used a between-subjects design (Condition: Experimental vs. Control). We invited USA nationals to participate through TurkPrime and UK nationals to participate through Prolific Academic, a UK-based crowdsourcing platform. In total, 932 were recruited to participate in the survey. For the purpose of the experiment, we focus only on white participants’ intergroup attitudes toward fellow citizen and immigrant target groups, resulting in 793 cases. The participants were randomly assigned to either the experimental or control condition. The experimental condition consisted of a wise reasoning reflection exercise that present participants with questions designed to prompt wise reasoning in reflection about conflicts. The control condition did not include the wise reasoning exercise. All participants read a news article about immigration issues. Participants in the Experimental Condition provided open-ended responses to the wise reasoning questions, whereas participants in the control conditions simply moved on to the next part of the study: All participants reported their positivity toward immigrants and home-country citizens, their attitudes and behavioral intentions, and we assessed their wise reasoning. Participants then completed a demographics questionnaire. Given the politicized nature of the conflicts about immigration, we also controlled for political orientation. Ostensibly upon completion of the survey, were given a bonus of $.50 for completing the survey. We then introduced them to a charity organization devoted to helping immigrants in their country. They were told that they were free to use their bonus to donate to the charity, if they wished; they could also provide their email address, if they wished to subscribe to volunteer opportunities to help immigrants. Out of the 793 participants, 776 completed the survey and passed attention check questions (WRE: *n* = 363; Control: *n* = 413; <3% attrition). This sample size gives us 80% power to detect a small-to-medium minimum effect size of the manipulation effect on wise reasoning (i.e., η^2^ = .03 or d = .33; Faul et al., 2009).

**Measures.** Verbatim study materials are presented in the Study Materials section in SI. The differences between the study materials for the USA sample and the UK sample are highlighted below.

***Wise reasoning exercise.*** Participants were told that they would be presented with a news article. In the WRE, participants were told to contemplate the issues in the article with four reflection questions from an observer perspective (i.e., using third-person pronouns: he/she/the participant’s own name). After reading the article, they responded via open-text to four wise reasoning reflection questions. See Study Materials in this document for full manipulation. Participants in the Control Condition did not receive any reflection questions and simply read the news article.

***Intergroup reflection.*** For the American sample, we used a news clipping from the CNN website reporting on the protests that were happening at the time on Capitol Hill in response to the expiring Deferred Action for Childhood Arrivals program that protects the second-generation children of illegal immigrants. For the UK sample, we used a news clipping from the Daily Mail website reporting on immigration in the UK.

***Intergroup bias.*** We measured positivity toward “Immigrants” and “U.S. Citizens”/ “U.K. Citizens” among other filler group targets using a feeling thermometer. The presentation order of the target groups was randomized to prevent any potential order effect.

***Motivation for intergroup contact.*** We asked participants, “If you had the opportunity, to what extent would you be willing to meet with people who hold opinions very different from yours about immigration and hear their point of view? Participants responded on a 6-point scale, from 1 = *Not at all* to 6 = *Very Much*.

***Endorsement of anti-immigration policies.*** We created 11 items that measured endorsement of policies that would create a hostile environment for immigrants (the opposite of conciliatory policies). For instance, “Immigrants should not share our facilities (e.g., schools; hospitals)” and “Immigrants should not be allowed to own land.”. Participants responded on a 7-point scale, from 1 = *Strongly oppose* to 7 = *Strongly in favor*. Factor analysis of the items revealed a single-factor solution (Eigenvalue = 7.72, 70% of variance; Principal axis factoring with direct oblimin rotation). We used the average score as the overall index of hostile policy endorsement (*α* = .96).

***Wise reasoning.*** Participants completed the 21-item measure of wise reasoning (*α* = .96).

***Political orientation.*** Political orientation was measured with three items assessing political views on foreign policy, economic, and social policy issues, which we combined due to their high intercorrelation (*r*s>.68). Reliability of this measure was high (*α*>.90).

**Behavioral outcomes.** We included two measures of concrete actions that participants could take to help immigrants: Voluntary donation and email subscription.

***Donation to help immigrants.*** Ostensibly upon completing the survey, participants were told that they would receive a $.50/£.50 bonus for their participation. They could donate their bonus to the *International Rescue Committee* (https://www.rescue.org) to help immigrants in the USA or to *Migrant Help* (<https://www.migranthelpuk.org/>) to help immigrants in the UK Specifically, they were asked, “Would you be interested in making a donation to International Rescue Committee/Migrant Help?” We assured participants that they could keep all of their bonus if they wished, that they were under no obligation to donate, and that their decision would be kept anonymous (see verbatim instructions of Donation in SI). The donation they made was a real donation that impacted their total payment from study participation. We used participants’ donation amount as a measure of helping behavior.

***Email subscription to volunteer.*** We measured whether participants would subscribe to volunteer to help immigrants. After the donation decision, we presented participants with an open response box in which they could enter their email address to receive volunteer opportunities with *International Rescue Committee*/ *Migrant Help*. Participants could enter an anonymous email to protect their privacy. Those who provided an email were coded as 1, and the rest were coded as 0.

**Detailed Analysis.** The following include tests with (subscript A) and without (subscript B) control variables. We first tested whether the wise reasoning exercise increased wise reasoning. Results of an independent *t-*test revealed that participants in the WRE (*M*_A_ = 3.52, *SD* = .74, *M*_B_ = 3.52, *SD* = .74) indeed reported stronger wise reasoning than those in the Control Condition (*M*_A_ = 3.18, *SD* = .83, *M*_B_ = 3.18, *SD* = .83), *t*_A_ (773) = 5.97, *p* < .001, η^2^_p_ = .04, 95% CI [.23, .45], *t*_B_ (775) = 5.95, *p* < .001, η^2^_p_ = .04, 95% CI [.23, .45]. To test the main hypotheses, we submitted feelings toward the ingroup and outgroup targets to a mixed-model regression, entering Condition as the moderating factor. We found a significant interaction, *F*_A_ (1, 770)= 4.75, *p =* .030, η^2^*_p_* = .01, *F*_B_ (1, 772)= 3.97, *p =* .047, η^2^*_p_* = .01, suggesting that the wise reasoning exercise reduced intergroup bias. Here, we observed less attitude polarization in the WRE. Looking at the target groups individually we found that reduced polarization was a result of a combined i) trend of stronger positivity toward the outgroup immigrants, *t*_A_ = 1.53, *p* = .126, η^2^*_p_* = .003, 95% CI [-.83, 3.89], *t*_B_ = 1.47, *p* = .142, η^2^*_p_* = .003, 95% CI [-.91, 6.34] and ii) slightly less positivity toward the ingroup home citizens, *t*_A_ = -1.27, *p* = .204, η^2^*_p_* = .002, 95% CI [-5.72, .71], *t*_B_ = -1.20, *p* = .232, η^2^*_p_* = .002, 95% CI [-3.82, .93]. In turn, less attitude polarization related significantly to willingness to meet outgroups, *F*_A_ (1, 770)= 7.712, *p =* .006, η^2^*_p_* = .01, *F*_B_ (1, 772)= 9.86, *p =* .002, η^2^*_p_* = .01, endorsement of hostile immigration policies, *F*_A_ (1, 770)= 246.53, *p* < .001, η^2^*_p_* = .24, *F*_B_ (1, 772)= 465.93, *p<=* .001, η^2^*_p_* = .38, marginally to subscribing to volunteer opportunities, *F*_A_ (1, 770)= 3.466, *p =* .063,^[[5]](#footnote-5)^ η^2^*_p_* = .004, but significantly when not controlling for USA/UK and political orientation, *F*_B_ (1, 772)= 9.94, *p =* .002, η^2^*_p_* = .01, opting to donate to charities that assist recent immigrants, *F*_A_ (1, 770)= 24.775, *p* < .001, η^2^*_p_* = .03, *F*_B_ (1, 772)= 37.60, *p <* .001, η^2^*_p_* = .05, and actual amount donated to the charities, *F*_A_ (1, 769)= 17.666, *p* < .001, η^2^*_p_* = .04, *F*_B_ (1, 771)= 31.14, *p <* .001, η^2^*_p_* = .04.

***Mediation models.*** Finally, we tested whether reduced attitude polarization would mediate the effect of the wise reasoning exercise on downstream outcomes. We present the results of these tests with two caveats that we did not find total effects of the exercise on downstream outcomes except for a borderline negative effect on subscribing to volunteer (see Table S3), and that mediation models do not infer causality in this case because these particular variables were measured at the same timepoint as attitude polarization and were not manipulated. Nonetheless, for illustrative purposes we tested the path: Condition 🡪 Attitude Polarization 🡪 Downstream Outcomes, using the PROCESS macro for SPSS (“Model 4” with 10,000 bootstrapped sample ^46^) with a separate analysis for each of the outcome variables, entering Condition as the independent variable, polarization as the mediator, and i) behavioral intentions and ii) actual behaviors as dependent variables. In these tests, the WRE decreased polarization, *B*_A_ = -3.77, *SE =* 1.90, *t*(770) = -2.08, *p* = .044, 95% CI[-0.22, -7.68], *B*_B_ = -4.16, *SE =* 2.09, *t*(772) = -1.99, *p* = .047, 95% CI[-8.26, -.06]. As seen in Table S8, in mediation tests with control variables the wise reasoning exercise had significant indirect effects on endorsing hostile immigration policies, opting to donate, and actual amount donated to charities that assist new immigrants; in mediation tests without control variables, all indirect effects of the intervention were significant. Interestingly, once entered into the mediation model, a negative effect of the intervention on subscribing to volunteer opportunities became significant, *B*_A_ = -.41, *SE =* .20, *z*(770) = -2.04, *p* = .041, 95% CI[-.82, -.02], *B*_B_ = -.43, *SE =* .20, *z*(772) = -2.11, *p* = .035, 95% CI[-.82, -.03], albeit in the presence of a positive indirect effect.

### Supplementary Table 8. Indirect and direct effects of a brief online wise-reasoning exercise on intergroup intentions and behaviors.

| **Outcome Variable** | **Indirect Effect** | **Direct Effect** |
| --- | --- | --- |
| ***With Control Variables*** |  |  |
| Meet outgroup | *B*=.02, *SE=*.01, 95% CI[-.0004, 0.04] | *B*=.09, *SE=*.11, 95% CI[-.12, .29] |
| Hostile immigrant policies | ***B*=.09, *SE=*.05, 95% CI[-.19, -.004]** | *B*=.07, *SE=*.08, 95% CI[-.09, .23] |
| Subscribe to volunteer | *B*=.03, *SE=*.02, 95% CI[-.003, .09] | ***B*=-.41, *SE=*.020, 95% CI[-.81, -.02]** |
| Opt to donate | ***B*=.06, *SE=*.03, 95% CI[.001, .13]** | *B*=-.24, *SE=*.16, 95% CI[-.56, .08] |
| Donation amount | ***B*=.005, *SE=*.002, 95% CI[.0002, .01]** | *B*=-.02, *SE=*.01, 95% CI[-.05, .01] |
| ***Without Control Variables*** |  |  |
| Meet outgroup | ***B*=.02, *SE=*.02, 95% CI[.0000, .06]** | *B*=.08, *SE=*.11, 95% CI[-.12, .29] |
| Hostile immigrant policies | ***B*=-.14, *SE=*.07, 95% CI[-.28, -.003]** | *B*=.09, *SE=*.09, 95% CI[-.09, .27] |
| Subscribe to volunteer | ***B*=.05, *SE=*.03, 95% CI[.0002, .12]** | ***B*=-.43, *SE=*.20, 95% CI[-.82, -.03]** |
| Opt to donate | ***B*=.08, *SE=*.04, 95% CI[.002, 0.17]** | *B*=-.25, *SE=*.16, 95% CI[-.57, .07] |
| Donation amount | ***B*=.01, *SE=*.003, 95% CI[.0001, .01]** | *B*=-.001, *SE=*.01, 95% CI[-.05, .01] |

*Note*. Significant effects in bold.

## Study 7

Study 7 was a pre-registered extension of Study 6 with the objective to address whether wise reasoning manipulation specifically increases wise reasoning and reduces attitude polarization beyond that of any general deliberation process—does wise reasoning minimize intergroup bias beyond the mere act of reflecting on a situation? We tested the utility of a Wise Reasoning Experimental (WRE) Condition for i) prompting wise reasoning and ii) reducing attitude polarization, compared to *both* a Pure Control (PC; as in Study 6) Condition and an additional Active Control (AC) Condition. This study was conducted in the context of intergroup bias during COVID-19 pandemic in 2020.

At the end of 2019, there was an outbreak of COVID-19, with mass infections recorded in Wuhan, China, initially. Amid debates and protests about the economic tradeoff of lockdowns and the drastic change in the way of life, some countries, such as the USA, showed a spike in negativity toward Chinese and Asian people in general ^47–49^. To better understand the phenomena, we conducted pilot studies to examine white American citizens’ attitudes toward different social targets. We observed attitude polarization toward their ingroup (i.e., “U.S. citizens” and “whites in the U.S.”) and their perceived outgroup (i.e., “Chinese citizens” and “Asians in the U.S”). Furthermore, attitude polarization was stronger among those who are more (vs. less) politically conservative. In June 2020, we pre-registered and conducted Study 7 against the backdrop of COVID-19 to examine intergroup bias and attitude polarization in the USA (see full pre-registration at <https://osf.io/yzx7e>). We recruited white American citizens to complete an online survey. Participants were randomly assigned to one of three conditions: WRE, AC, or PC. Based on the pilot data, we pre-registered four main hypotheses:

Hypothesis 1: Self-report of wise reasoning is higher in WRE than the Control Conditions (AC and PC)

Hypothesis 2: Target Group Membership interacts with the Conditions, such that those in WRE (vs. AC and PC) showed the least polarized intergroup attitude.

Hypothesis 3: Political conservatism is positively associated with polarization in intergroup attitude.

Hypothesis 4: Conditions moderate the relationship between political orientation and intergroup polarization, such that the relationship is weaker in WRE (vs. AC and PC).

**Participants, Procedure, and Power.** This experiment used a between-subjects design (Condition: WRE vs. AC vs. PC). In a pilot study conducted among U.S. citizens (*n* = 114; WRE= 30; AC = 34; CC = 50), the effect size (f) of the interaction between conditions and political orientation on the intergroup polarization score was (.05; or η^2^*_p_* = .016). A power analysis (G*power 3.1.7) based on standard alpha (.05) assuming non-sphericity correction as 1 suggested that to achieve statistical power of .90 would require a total sample of 273. However, considering i) the volatility of the COVID-19 situation, ii) the dynamic of USA-China relations, iii) the possibility that the true effect size is smaller than found in the pilot study, and iv) the possibility that robot responses and respondents who failed attention check would be filtered from analyses, we aimed to recruit a larger sample of 900 participants (300 per condition), who identified as white American citizens through Prolific Academic. In total, 903 participants responded to the survey.

All participants read a news article about COVID-19 and were randomly assigned to one of the three conditions. Participants in WRE and AC provided open-ended responses, whereas participants in PC did not provide open-ended responses. All participants reported their positivity toward ingroup and outgroup targets (see below), and their wise reasoning. Participants then completed a demographics questionnaire. We focus only on white American citizens living in the USA. The following responses were excluded from analyses: eleven participants who were non-white, six participants who were non-citizen or permanent resident, and sixteen participants who were not living in the USA were excluded from analyses. One participant had missing data on the outcome variable and was excluded. Eighty participants who missed attention check item and four responses that were identified as incomprehensible or robot responses via analysis of written responses were also excluded. Our final sample consisted of 791 cases. (WRE: *n* = 238; AC = 271; PC: *n* = 282; .097% attrition). Based on sensitivity power analysis (G*power 3.1.7; Faul et al., 2009), this sample size would give us 80% power to detect the experimental effect on wise reasoning as small as *d* = .20/.22 (WRE vs. control/WRE vs. AC vs. PC), and an interaction effect between Conditions and political orientation as small as η^2^ = .003/.004 (or d = .12; WRE vs. control/WRE vs. AC vs. PC).

**Measures.** Verbatim study materials are presented in the Study Materials section in SI.

***Experimental conditions.*** We amalgamated the news article from two different news outlets ([Vox](https://www.vox.com/2020/4/28/21234598/coronavirus-china-xi-jinping-foreign-policy) and [New York Times](https://www.nytimes.com/2020/06/07/world/asia/china-coronavirus.html)), using the text and images that i) tap into the ongoing COVID-19 controversy related to intergroup bias, and ii) would appear to come from an anonymous news media source so as to not bias via news outlet preferences. All participants were told that they would be presented with a randomly sampled news article. After reading the article, they were randomly assigned to one of three conditions: WRE, AC, and PC. In the WRE Condition, the participants responded via open-text to four wise reasoning-related reflection questions (e.g., How might the situation change in time? What might be the uncertainties surrounding this situation?) in a large essay box. In the AC Condition, the participant responded via open-text to four *non*-wise reasoning-related reflection questions (e.g., What is your stance on this situation? What is your first [second; third] reason you have for your stance?) in a large essay box. In PC, the participants simply read the article and did not receive any reflection questions.

***Political orientation.*** Participants responded to the prompt, “Please let us know your stance on political issues:” on from 1 (*Very* *Liberal, 4* = *Middle of the road/Neutral*, 7 = *Very Conservative*). They responded to three sliders labelled as “Social Policies”, “Economic Policies”, and “Foreign Policies”). Responses were strongly intercorrelated (.77 < *r*s < .82), so we calculated a mean score as indicator of political orientation (*α* = .92).

***Intergroup bias.*** We measured positivity toward “Chinese Citizens,” “Asians in the U.S.” using feeling thermometers. Principal components analysis on these items indicated a single component explaining 73.96% of the variance, so we combined them as a proxy of the outgroup target. We also measured positivity toward “American Citizens” and “whites in the U.S.” using feeling thermometers. Principal components analysis on these items indicated a single component explaining 83.22% of the variance, so we combined them as a proxy of the ingroup target. Given that participants might conflate their feelings toward ingroup and outgroup people with the respective governments (e.g., the news article reported the attribution of blame to the governments), we also measured feeling thermometer ratings toward “Chinese government” and “U.S. government” for ancillary exploration. The target groups were interspersed with other filler groups.

***Wise reasoning.*** Participants completed the 21-item measure of wise reasoning (*α* = .95).

**Detailed Analysis.** Because our pre-registered hypotheses were unidirectional—specifically that polarization would be weaker in the WRE Condition, as compared to AC and PC Conditions—we present both the results of 1-tailed and 2-tailed significance tests when applicable. Presented here are results focusing on attitudes toward human target groups (i.e., Chinese/Asian people, American/white people); we present exploratory tests including attitudes toward Chinese and U.S. Government targets in Table S9.

We first tested whether the wise reasoning exercise increased wise reasoning. Results of an independent *t-*test revealed that participants in WRE indeed reported stronger wise reasoning (*M* = 3.52, *SD* = .70) than those in the Control Conditions (*M*_AC_ = 3.25, *SD* = .80, *M*_PC_ = 3.05, *SD* = .84): *t*_WRE vs. AC_ (788) = 3.87, *p_1- & 2-tailed_* < .001, η^2^_p_ = .02, 95% CI [.13, .47]; *t*_WRE vs. PC_ (788) = 6.84, *p_1- & 2-tailed_* < .001, η^2^_p_ = .06, 95% CI [.34, .61]. The findings indicated support for Hypothesis 1. While the means are closer between AC and PC, their difference was significant, *t* (788) = 3.03, *p* =.003, η^2^_p_ = .01. As such, in the following we will present analyses with the two control (AC & PC) conditions *combined* for parsimony and increased power of the analyses, and with the control conditions *separate* for transparency of findings. The results of these latter tests are presented in brackets or otherwise explicitly stated.

To test the second hypothesis that participants would show less attitude polarization in the WRE Condition, we submitted feelings toward the ingroup and outgroup targets to a mixed-model regression, entering Condition as the predictor. Results are presented in full in the upper partition of Table S9. We found a non-significant interaction between Condition and Target Group membership, *F* (1, 789) = 1.932, *_1-/2-tailed_* = .083/.165, η^2^*_p_* = .002 (WRE vs. AC vs. PC: *F* (1, 788)= 0.980, *p_1-/2-tailed_* = .141/.376, η^2^*_p_* = .001), but as expected, WRE produced i) significantly stronger positivity toward the outgroup (*p_1-/2-tailed_* = .006/=.011). It also showed ii) a weaker, nonsignificant trend toward stronger positivity toward the ingroup (*p* = .362). Though the effects of WRE was driven by the increase in positivity toward the outgroup (not ingroup), this combination of effects explained the weak interaction. Nonetheless, as shown in Table S9, attitude polarization (the difference between ingroup vs. outgroup positivity) was smaller and nonsignificant in WRE, whereas the polarization was larger and significant in the Control Conditions. Further, participants in the WRE showed more positivity toward the outgroup than participants in the Control Conditions, whether combined or taken separately. [Similar effects were found when analyzing the three conditions separately: Participants in the WRE condition showed: i) significantly stronger positivity toward the outgroup compared to AC (*p_1-/2-tailed_* = .002/.043), and PC (*p_1-/2-tailed_* < .001/=.015), and ii) no difference in positivity toward the ingroup compared to AC (*p* = .600) and iii) a trend of stronger positivity toward the ingroup compared to PC (*p* =.287)]. Altogether, these findings indicated partial support for Hypothesis 2.

Testing the relation between political orientation, we found that stronger conservative orientation correlated with more positivity toward the ingroup (*r* = .507, *p* < .001) and less positivity toward the outgroup (*r* = -.098, *p* = .006). As indicated by the preliminary correlations (Table S3), we found that conservatism was a strong predictor of heightened attitude polarization, *F* (1, 787) = 156.889, *p* < .001, η^2^*_p_* = .17, supporting Hypothesis 3.

To test the fourth hypothesis, we submitted feelings toward the ingroup and outgroup targets to a mixed-model regression, entering political orientation, Condition, and their interaction as predictors in the model. Results are presented in full in the lower partition of Table S9. A significant 3-way interaction emerged between political orientation, Condition, and target group, *F* (1, 787) = 4.808, *p* *_1-/2-tailed_* < .001/=.029, η^2^*_p_* = .01 [WRE vs. PC vs. AC: *F* (1, 785)= 2.494, *p* *_1-/2-tailed_* = .007/=.083, η^2^*_p_* = .01], indicating that the effect of conservatism on attitude polarization differed significantly for participants who had partaken in the wise reasoning exercise. To examine the moderating effect of wise reasoning, we next tested the relation between conservatism and feelings toward in- and outgroup separately per condition. Whereas political conservatism predicted much more positivity toward the ingroup for participants in the control conditions (*t*=9.38, *p* < .001) the effect was weaker for participants in WRE (*t*=5.78, *p* < .001); and, whereas conservatism predicted less positivity toward the outgroup among participants in the Control Conditions (*t*=-3.95, *p* < .001), it did not among those in the WRE (*t*=-.41, *p* =.648). Taken together, participants in the Control Conditions showed much stronger politically-oriented intergroup attitude polarization than those in the WRE. These results indicate full support for the fourth hypothesis, that wise reasoning exercise would reduce the effect of political orientation on intergroup attitude polarization. [PC vs. AC: conservatism predicted: i) stronger positivity toward the ingroup in the AC Condition (*p* < .001) and the PC Condition (*p* < .001), and ii) less positivity toward the outgroup in the AC Condition (*p* < .001) and the PC Condition (*p* =.052), resulting in iii) strong intergroup attitude polarization in the AC Condition (*p* < .001) and in the PC Condition (*p* < .001)].

***Supplementary analyses including governmental targets as part of the dependent variable***. To be thorough in our analyses, we report additional analyses including feelings to the U.S. and to the Chinese Governments as part of the ingroup and outgroup positivity measures respectively. This attempt was motivated by two observations. Several participants’ written response focused on the tension between U.S. and China, and the news article stimulus mentioned specific conflicts at the inter-government level; as such, participants’ feelings toward the governments may capture potentially meaningful variance of the intergroup bias construct. These results are presented in Table S9 in lightened fonts. Overall, we found polarization in all condition. The means in the WRE condition were significantly different from the Control Conditions—the effect was driven by more negative and differential feelings to governmental targets. In sum, results were consistent with or without including governmental targets as part of the dependent variable.

### Supplementary Table 9. Results of Study 7: Tests of attitude polarization as a function of Conditions and political orientation.

| **Study 7** (*n=*791) | | **2-Way Condition x Factor Interaction**  (Hypothesis 2) | | |
| --- | --- | --- | --- | --- |
|  | WRE vs. Controls (AC & PC) | *F*(1, 789) = 1.932, *p*_1-/2-tailed_ = .083/.165, η2*_p_* = .002  *F*(1, 789) = 2.300, *p*_1-/2-tailed_ = .065/.130, η2*_p_* = .003 | | |
|  |  |  | | |
|  | WRE vs. AC vs. PC | *F*(1, 788) = 0.980, *p*_1-/2-tailed_ = .188/.376, η2*_p_* = .003  *F*(1, 788) = 1.242, *p*_1-/2-tailed_ = .145/.289, η2*_p_* = .003 | | |
|  |  |  | | |
|  |  | ***Mean Positivity Toward***  ***Outgroup Targets*** | ***Polarization*** | ***Mean Positivity Toward***  ***Ingroup Targets*** |
|  | WRE | 66.548  51.945 | *t*=2.34, *p* = .020  *t*=6.07, *p* < .001 | 69.540  60.011 |
|  | Controls Combined | 62.906  48.274 | *t*=5.84, *p* < .001  *t*=11.55, *p* < .001 | 68.121  58.831 |
|  | PC | 62.592  47.541 | *t*=4.18, *p* < .001  *t*=8.27, *p* < .001 | 67.656  57.718 |
|  | AC | 63.233  49.036 | *t*=4.07, *p* < .001  *t*=8.07, *p* < .001 | 68.605  59.990 |
|  |  | **3-Way Political Orientation x Condition x Factor Interaction**  (Hypothesis 4) | | |
|  | WRE vs. Controls (AC & PC) | *F*(1, 787) = 4.808, *p* = .029, η2*_p_* = .006  *F*(1, 787) = 8.459, *p* = .004, η2*_p_* = .011 | | |
|  |  |  | | |
|  | WRE vs. AC vs. PC | *F*(1, 785) = 2.494, *p* = .083, η2*_p_* = .006  *F*(1, 785) = 4.337, *p* = .013, η2*_p_* = .011 | | |
|  |  |  | | |
|  |  | ***Political Orientation***  ***🡪 Outgroup Positivity*** | ***Political Orientation***  ***🡪 Ingroup Positivity*** | ***Political Orientation 🡪 Attitude Polarization*** |
|  | WRE | *t*=-.41, *p* =.684  *t*=.20, *p* =.843 | *t*=5.78, *p* < .001  *t*=7.89, *p* < .001 | *t*=6.02, *p* < .001  *t*=6.90, *p* < .001 |
|  | Controls Combined | *t*=-3.95, *p* < .001  *t*=-3.28, *p* < .001 | *t*=9.38, *p* < .001  *t*=14.62, *p* < .001 | *t*=13.90, *p* < .001  *t*=17.52, *p* < .001 |
|  | PC | *t*=-1.95, *p* = .052  *t*=-1.90, *p* = .058 | *t*=7.11, *p* < .001  *t*=10.50, *p* < .001 | *t*=9.39, *p* < .001  *t*=12.38, *p* < .001 |
|  | AC | *t*=-3.60, *p* < .001  *t*=-2.73, *p* = .007 | *t*=6.13, *p* < .001  *t*=10.10, *p* < .001 | *t*=10.24, *p* < .001  *t*=12.33, *p* < .001 |

*Note*. Mixed-model regressions. One- and two-sided tests in upper partition; two-sided tests in lower partition. WRE= Wise Reasoning Experimental Condition (*n* = 238); PC = Pure Control Condition (*n* = 282); AC = Active Control Condition (*n* = 271). Lightened font: Results of analyses when including governmental targets as part of the dependent variable.

***Exploratory Content Analysis of Written Responses: Wise Reasoning Experimental vs. Active Control.*** We also examined differences in the participants’ writing between WRE and AC conditions. These analyses shed light on 1) potential differences between wise reasoning and more general deliberative processes, and 2) potential mediating mechanisms that explain WRE’s effects on attitude polarization. To do so, we submitted all written responses to the Linguistic Inquiry and Word Count (LIWC) software ^50^, which is a validated text analysis software that detects psychologically meaningful differences in writing ^51^.

Using the software, we categorized participants’ choice of words based on the 2015 LIWC dictionary. Within each participant’s response, we counted the proportion of word use that reflected certain psycholinguistic categories validated by prior research. A higher frequency of the word count in a category (e.g., anxiety-related words), the higher intensity of the psychological processes associated with the category of the participant when writing (e.g., anxiety) ^50^. The LIWC manual contains further detail about the development of the software, the definition and examples of each psycholinguistic category, and the validity evidence of each category; the most recent version of the manual at the time of the study can be found here: <https://repositories.lib.utexas.edu/bitstream/handle/2152/31333/LIWC2015_LanguageManual.pdf>

Based on the LIWC analysis, we first tested the correlation between the LIWC results with the conditions, political orientation, and attitude polarization. The correlations are presented in Table S10. As evidenced by the correlations in the table, participants’ writing in WRE and AC differed. For instance, compared to AC, those in WRE wrote more (i.e., higher word count), described more social processes, used larger words (words with more than 6 letters), used more tentative language (e.g., maybe, perhaps), and used fewer personal pronouns and common verbs. Among these significant differences between the conditions, four categories were also correlated with attitude polarization and self-report wise reasoning. They include past focus, tentative language, the use of larger words, and use of 3^rd^-person plurals (e.g., ‘they’).

Mediating models. We proceeded to examine whether the above four categories might meaningfully explain the effects of WRE (vs. AC), as observed earlier. To do so, we subjected the four categories as simultaneous mediators in a mediation model using PROCESS on SPSS 24 with 10,000 bootstrap samples (“Model 4” with 10,000 bootstrapped sample ^46^). Word count was entered as a control in all subsequent analyses to make sure the length of writing, or the amount of mere deliberation, was not driving the effects, though with or without the control did not affect the pattern of the following results. As presented in Table S11, two of the variables emerged to be possible mediators: the use of larger words and 3^rd^-person plurals. WRE (vs. AC) increased the use of larger words (*B* = 2.76, *SE* = .50, *p* < .001) and decreased the use of 3^rd^-person plural (e.g., ‘they’; *B* = -.51, *SE* = .19, *p* = .008), in turn, both pathways were associated with reduced attitude polarization, i.e., the negative indirect effects. Next, we also tested whether the same four psycholinguistic categories would explain the moderating effect of WRE on the relation between political orientation and attitude polarization. To do so, we entered the four categories as simultaneous mediators in a moderated mediation model (“Model 8”). As seen in Table S11, one variable emerged to be a significant mediator of the interaction (the moderation effect of WRE). Specifically, among those in the AC condition, political conservatism was related to using 3^rd^-person plurals more in their writing (*B* = .340, *SE* = .08, *p* < .001), which in turn was associated with increased attitude polarization. In contrast, the negative relationship between political conservatism and the use of 3^rd^-person plurals was weaker in the WRE condition (*B* = .18, *SE* = .07, *p* = .015), and therefore the indirect association between political conservatism and increased attitude polarization was no longer significant. However, the difference between the two indirect effects did not reach statistical significance, *B* = -.18, *SE* = .14, 95% CI[-.51, .03].

Together, the exploratory mediation analyses showed preliminary evidence for why the wise reasoning exercise could be linked to reduced attitude polarization, specifically its ability to counter people’s thinking about the outgroup as “them.” This finding is aligned with intergroup research showing that “we-they” thinking—differentiating outgroup members as invariant and categorically different from the ingroup—to be a problematic root of intergroup bias ^52–55^, and demonstrated a process in which wise reasoning reduces polarization.

### Supplementary Table 10. Correlations between the LIWC results and key study variables.

|  | WRE vs. AC | |  | Political orientation  (higher more conservative) | |  | Attitude Polarization (ingroup - outgroup) | |  | Self-report Wise Reasoning | |
| --- | --- | --- | --- | --- | --- | --- | --- | --- | --- | --- | --- |
|  | *r* | *p-value* |  | *r* | *p-value* |  | *r* | *p-value* |  | *r* | *p-value* |
| Word count | .133 | .003 |  | -.214 | <.001 |  | -.162 | <.001 |  | .018 | .680 |
| Words/sentence | -.032 | .478 |  | -.097 | .029 |  | -.088 | .047 |  | .043 | .335 |
| Words > 6 letters | **.248** | **<.001** |  | **-.190** | **<.001** |  | **-.182** | **<.001** |  | **.113** | **.010** |
| Dictionary words | -.090 | .042 |  | .102 | .021 |  | .083 | .061 |  | -.035 | .426 |
| Total function words | -.210 | <.001 |  | .047 | .289 |  | .057 | .202 |  | -.099 | .026 |
| Total pronouns | -.422 | <.001 |  | .12 | .007 |  | .096 | .030 |  | -.059 | .185 |
| Personal pronouns | -.438 | <.001 |  | .162 | <.001 |  | .130 | .003 |  | -.076 | .088 |
| 1st person singulars (e.g., I) | -.434 | <.001 |  | .034 | .441 |  | -.016 | .726 |  | -.021 | .629 |
| 1st person plurals (e.g., we) | -.019 | .663 |  | -.032 | .466 |  | .025 | .569 |  | -.022 | .622 |
| 2nd persons (e.g., you) | -.024 | .594 |  | .010 | .823 |  | -.012 | .785 |  | -.023 | .600 |
| 3rd person singulars (e.g., she) | -.029 | .521 |  | .075 | .090 |  | .016 | .726 |  | .076 | .087 |
| 3rd person plurals (e.g., they) | **-.129** | **.003** |  | **.229** | **<.001** |  | **.224** | **<.001** |  | **-.102** | **.021** |
| Impersonal pronouns (e.g., it) | -.154 | <.001 |  | -.003 | .949 |  | -.002 | .967 |  | -.005 | .919 |
| Article | .314 | <.001 |  | -.109 | .014 |  | -.077 | .085 |  | .016 | .721 |
| Prepositions | .107 | .016 |  | -.004 | .921 |  | .021 | .633 |  | -.100 | .025 |
| Auxiliary verbs | -.148 | .001 |  | .043 | .335 |  | .033 | .463 |  | -.057 | .202 |
| Common adverbs | -.136 | .002 |  | .009 | .841 |  | .038 | .388 |  | -.013 | .765 |
| Conjunctions | .125 | .005 |  | -.070 | .114 |  | .012 | .787 |  | .086 | .053 |
| Negations | -.157 | <.001 |  | .015 | .739 |  | -.053 | .236 |  | -.039 | .379 |
| Common verbs | -.269 | <.001 |  | .144 | .001 |  | .120 | .007 |  | -.059 | .181 |
| Common adjectives | -.018 | .678 |  | .054 | .224 |  | .058 | .191 |  | .028 | .531 |
| Comparisons | .117 | .008 |  | -.045 | .311 |  | .039 | .383 |  | -.009 | .836 |
| Interrogatives | -.028 | .527 |  | .002 | .966 |  | .059 | .183 |  | .037 | .405 |
| Numbers | -.104 | .019 |  | .027 | .548 |  | .033 | .451 |  | -.033 | .463 |
| Quantifiers | .056 | .208 |  | .011 | .810 |  | -.021 | .639 |  | -.004 | .933 |
| Affective processes | .010 | .824 |  | .113 | .011 |  | .060 | .178 |  | .028 | .535 |
| Positive Emotions | .045 | .311 |  | .093 | .037 |  | .009 | .832 |  | .044 | .317 |
| Negative Emotions | -.038 | .397 |  | .055 | .217 |  | .079 | .076 |  | -.025 | .567 |
| Anxiety | .142 | .001 |  | .004 | .933 |  | .021 | .633 |  | .097 | .028 |
| Anger | -.040 | .374 |  | .033 | .458 |  | .057 | .202 |  | -.098 | .027 |
| Sadness | .033 | .454 |  | .070 | .116 |  | .057 | .202 |  | -.015 | .735 |
| Social Processes | .144 | .001 |  | .176 | <.001 |  | .147 | .001 |  | .017 | .699 |
| Family | -.091 | .040 |  | .119 | .007 |  | .073 | .098 |  | -.038 | .394 |
| Friends | -.061 | .167 |  | -.014 | .756 |  | .033 | .463 |  | .004 | .932 |
| Female references | -.033 | .451 |  | .078 | .078 |  | .145 | .001 |  | .076 | .085 |
| Male References | -.025 | .574 |  | .038 | .393 |  | -.005 | .905 |  | .038 | .395 |
| Cognitive processes | -.104 | .019 |  | -.049 | .272 |  | -.042 | .350 |  | .067 | .131 |
| Insight | -.263 | <.001 |  | .022 | .623 |  | -.032 | .474 |  | .039 | .385 |
| Causation | .048 | .280 |  | -.062 | .164 |  | .065 | .145 |  | .024 | .588 |

|  | WRE vs. AC | |  | Political orientation  (higher more conservative) | |  | Attitude Polarization  (ingroup - outgroup) | |  | Self-report Wise Reasoning | |
| --- | --- | --- | --- | --- | --- | --- | --- | --- | --- | --- | --- |
|  | *r* | *p-value* |  | *r* | *p-value* |  | *r* | *p-value* |  | *r* | *p-value* |
| Discrepancy | -.049 | .271 |  | -.017 | .697 |  | .036 | .420 |  | .039 | .377 |
| Tentative | **.085** | **.055** |  | **-.076** | **.086** |  | **-.136** | **.002** |  | **.108** | **.015** |
| Certainty | -.059 | .180 |  | .002 | .959 |  | .010 | .815 |  | -.038 | .398 |
| Differentiation | -.005 | .919 |  | -.080 | .070 |  | -.100 | .024 |  | .024 | .594 |
| Perceptual processes | -.081 | .069 |  | .036 | .418 |  | -.007 | .878 |  | .067 | .128 |
| See | .096 | .031 |  | -.020 | .645 |  | -.01 | .829 |  | .005 | .919 |
| Hear | -.098 | .026 |  | -.021 | .637 |  | -.003 | .951 |  | -.053 | .233 |
| Feel | -.130 | .003 |  | .082 | .064 |  | .011 | .809 |  | .084 | .058 |
| Biological Processes | -.065 | .145 |  | .120 | .007 |  | .062 | .164 |  | -.013 | .772 |
| Body | .015 | .735 |  | .002 | .962 |  | -.002 | .964 |  | -.011 | .797 |
| Health | -.070 | .114 |  | .109 | .014 |  | .057 | .196 |  | -.013 | .763 |
| Sexual | .002 | .964 |  | .011 | .805 |  | .007 | .877 |  | .012 | .783 |
| Ingestion | -.068 | .125 |  | .079 | .075 |  | .046 | .304 |  | .011 | .812 |
| Drives | .201 | <.001 |  | .035 | .435 |  | .05 | .262 |  | -.014 | .749 |
| Affiliation | .242 | <.001 |  | .036 | .418 |  | <.001 | .992 |  | .104 | .019 |
| Achievement | .155 | <.001 |  | .047 | .295 |  | .03 | .502 |  | -.066 | .138 |
| Power | .109 | .014 |  | -.011 | .807 |  | .002 | .965 |  | -.140 | .002 |
| Reward | .010 | .817 |  | .076 | .085 |  | .049 | .265 |  | -.004 | .924 |
| Risk | -.046 | .303 |  | -.060 | .174 |  | .033 | .453 |  | .056 | .209 |
| Past Focus | **-.266** | **<.001** |  | **.108** | **.015** |  | **.107** | **.015** |  | **-.114** | **.010** |
| Present Focus | -.188 | <.001 |  | .139 | .002 |  | .039 | .383 |  | -.002 | .960 |
| Future Focus | .283 | <.001 |  | -.030 | .502 |  | .024 | .591 |  | .008 | .856 |
| Relativity | .193 | <.001 |  | .013 | .764 |  | .016 | .726 |  | .045 | .314 |
| Motion | .093 | .035 |  | .007 | .867 |  | .017 | .706 |  | .037 | .401 |
| Space | .296 | <.001 |  | -.021 | .634 |  | -.032 | .469 |  | .074 | .096 |
| Time | -.124 | .005 |  | .050 | .263 |  | .058 | .193 |  | -.055 | .216 |
| Work | .162 | <.001 |  | -.037 | .408 |  | -.074 | .095 |  | -.036 | .422 |
| Leisure | .284 | <.001 |  | .022 | .624 |  | -.042 | .348 |  | .129 | .003 |
| Home | -.074 | .094 |  | .035 | .432 |  | .021 | .633 |  | -.068 | .123 |
| Money | -.033 | .452 |  | .106 | .017 |  | .094 | .035 |  | .008 | .848 |
| Religion | -.088 | .046 |  | .037 | .408 |  | .011 | .808 |  | .006 | .887 |
| Death | -.030 | .504 |  | .069 | .120 |  | .07 | .117 |  | -.023 | .611 |
| Informal languages | -.137 | .002 |  | .011 | .800 |  | -.024 | .585 |  | -.013 | .770 |
| Swear words | -.084 | .057 |  | -.021 | .637 |  | -.025 | .574 |  | .026 | .562 |
| Netspeak | .021 | .640 |  | -.036 | .416 |  | -.064 | .148 |  | .044 | .322 |
| Assent | -.220 | <.001 |  | .038 | .395 |  | .004 | .934 |  | -.063 | .156 |
| Nonfluencies | .040 | .368 |  | .005 | .905 |  | <.001 | .996 |  | .001 | .989 |
| Fillers | .001 | .990 |  | .028 | .534 |  | -.019 | .670 |  | .029 | .520 |

*Note.* Pearson’s correlations; two-sided tests. Tested mediators in bold.

### Supplementary Table 11. Psycholinguistic variables as potential mediating mechanisms of the wise reasoning experimental (vs. active control) condition.

|  | **Indirect Effects** | | |
| --- | --- | --- | --- |
| Model | 1. WRE (vs. AC) ***🡪*** Mediator ***🡪*** Attitude Polarization | 1. Political Orientation × WRE (vs. AC) ***🡪*** Mediator ***🡪*** Attitude Polarization | |
|  |  | WRE | AC |
| Mediators |  |  |  |
| Past focus | *B*=.70, *SE=*.51, 95% CI[-1.74, .29] | *B*=.04, *SE=*.05, 95% CI[-.06, .16] | *B*=.05, *SE=*.07, 95% CI[-.07, .22] |
| Words > 6 letters | ***B*=-1.29, *SE=*.52, 95% CI[-2.36, -.35]** | *B*=.12, *SE=*.10, 95% CI[-.02, .37] | *B*=.18, *SE=*.12, 95% CI[-.02, .45] |
| 3^rd^-person plurals | ***B*=-.84, *SE=*.39, 95% CI[-1.69, -.19]** | ***B*=.15, *SE=*.10, 95% CI[-.01, .37]** | ***B*=.33, *SE=*.16, 95% CI[.05, .68]** |
| Tentative thinking | *B*=-.33, *SE=*.25, 95% CI[-.93, .06] | *B*=.02, *SE=*.07, 95% CI[-.14, .17] | *B*=.09, *SE=*.07, 95% CI[-.03, .25] |

*Note* Significant effects in bold.

# Supplementary References

1. Brienza, J. P., Kung, F. Y. H., Santos, H. C., Bobocel, D. R. & Grossmann, I. Wisdom, bias, and balance : Toward a process-sensitive measurement of wisdom-related cognition. *J. Pers. Soc. Psychol.* **115**, 1093–1126 (2018).

2. Faul, F., Erdfelder, E., Buchner, A. & Lang, A.-G. Statistical power analyses using G*Power 3.1: tests for correlation and regression analyses. *Behav. Res. Methods* **41**, 1149–1160 (2009).

3. Kaiman, J. Hong Kong’s umbrella revolution - the Guardian briefing. *Guardian* (2014).

4. Miller, A. G. Role of physical attractiveness in impression formation. *Psychon. Sci.* **19**, 241–243 (1970).

5. Fiske, S. T., Cuddy, A. J. C. & Glick, P. Universal dimensions of social cognition: warmth and competence. *Trends Cogn. Sci.* **11**, 77–83 (2007).

6. Anger at mainland visitors escalates with “locust” ad | South China Morning Post. *South China Morning Post* (2015).

7. Gervais, W. M., Shariff, A. F. & Norenzayan, A. Do you believe in atheists? Distrust is central to anti-atheist prejudice. *J. Pers. Soc. Psychol.* **101**, 1189 (2011).

8. Bellow, M. At Supreme Court, gay marriage protests divide a family. *USA Today* (2015).

9. ProCon.org. Background of the issue: Should gay marriage be legal? (2017). Available at: https://gaymarriage.procon.org/view.resource.php?resourceID=006275#. (Accessed: 11th December 2017)

10. van der Toorn, J., Jost, J. T., Packer, D. J., Noorbaloochi, S. & Van Bavel, J. J. In defense of tradition: Religiosity, conservatism, and opposition to same-sex marriage in North America. *Personal. Soc. Psychol. Bull.* **43**, 1455–1468 (2017).

11. Cowan, G., Heiple, B., Marquez, C., Khatchadourian, D. & McNevin, M. Heterosexuals’ attitudes toward hate crimes and hate speech against gays and lesbians: Old-fashioned and modern heterosexism. *J. Homosex.* **49**, 67–82 (2005).

12. Cianni, V. Gays in the Military: How America Thanked Me. *J. Gay Lesbian Ment. Health* **16**, 322–333 (2012).

13. Galinsky, A. D. & Moskowitz, G. B. Perspective-taking: decreasing stereotype expression, stereotype accessibility, and in-group favoritism. *J. Pers. Soc. Psychol.* **78**, 708 (2000).

14. Roets, A. & Van Hiel, A. Item selection and validation of a brief, 15-item version of the Need for Closure Scale. *Pers. Individ. Dif.* **50**, 90–94 (2011).

15. Todd, A. R. & Galinsky, A. D. Perspective-taking as a strategy for improving intergroup relations: Evidence, mechanisms, and qualifications. *Soc. Personal. Psychol. Compass* **8**, 374–387 (2014).

16. Wang, C. S., Lee, M., Ku, G. & Leung, A. K. y. K. -y. The cultural boundaries of perspective-taking: When and why perspective-taking reduces stereotyping. *Personal. Soc. Psychol. Bull.* **44**, 014616721875745 (2018).

17. Bruneau, E. G. & Saxe, R. The power of being heard: The benefits of “perspective-giving” in the context of intergroup conflict. *J. Exp. Soc. Psychol.* **48**, 855–866 (2012).

18. Galinsky, A. D. & Ku, G. The effects of perspective-taking on prejudice: The moderating role of self-evaluation. *Personal. Soc. Psychol. Bull.* **30**, 594–604 (2004).

19. Vorauer, J. D., Martens, V. & Sasaki, S. J. When trying to understand detracts from trying to behave: Effects of perspective taking in intergroup interaction. *J. Pers. Soc. Psychol.* **96**, 811–827 (2009).

20. Galinsky, A. D., Maddux, W. W., Gilin, D. & White, J. B. Why it pays to get inside the head of your opponent: The differential effects of perspective taking and empathy in negotiations. *Psychol. Sci.* **19**, 378–384 (2008).

21. Pierce, J. R., Kilduff, G. J., Galinsky, A. D. & Sivanathan, N. From glue to gasoline How competition turns perspective takers unethical. *Psychol. Sci.* **24**, 1986–1994 (2013).

22. Bloom, P. Empathy and its discontents. *Trends Cogn. Sci.* **21**, 24–31 (2017).

23. Grossmann, I. Wisdom in context. *Perspect. Psychol. Sci.* **12**, 233–257 (2017).

24. Webster, D. M. & Kruglanski, A. W. Individual differences in need for cognitive closure. *J. Pers. Soc. Psychol.* **67**, 1049–1062 (1994).

25. Roets, A. & Van Hiel, A. Allport’s prejudiced personality today: Need for closure as the motivated cognitive basis of prejudice. *Curr. Dir. Psychol. Sci.* **20**, 349–354 (2011).

26. Tadmor, C. T., Hong, Y., Chao, M. M., Wiruchnipawan, F. & Wang, W. Multicultural experiences reduce intergroup bias through epistemic unfreezing. *J. Pers. Soc. Psychol.* **103**, 750–772 (2012).

27. Brandt, M. J. & Reyna, C. The role of prejudice and the need for closure in religious fundamentalism. *Personal. Soc. Psychol. Bull.* **36**, 715–725 (2010).

28. Baltes, P. B. & Staudinger, U. M. The search for a psychology of wisdom. *Curr. Dir. Psychol. Sci.* **2**, 75–80 (1993).

29. Nobles, R., Tran, L. & Ly, L. Despite Supreme Court ruling, same-sex marriage conflicts continue. *CNN* (2015).

30. Matas, R. Knights entitled to deny lesbians, tribunal says. *The Globe and Mail* (2005).

31. BBC News. Canada approves “homophobic” law school. (2013).

32. Crawford, J. T., Brandt, M. J., Inbar, Y. & Mallinas, S. R. Right-wing authoritarianism predicts prejudice equally toward “gay men and lesbians” and “homosexuals.” *J. Pers. Soc. Psychol.* **111**, 31–45 (2016).

33. Rios, K. Right-wing authoritarianism predicts prejudice against “homosexuals” but not “gay men and lesbians.” *J. Exp. Soc. Psychol.* **49**, 1177–1183 (2013).

34. Davis, M. H. Measuring individual differences in empathy: Evidence for a multidimensional approach. *J. Pers. Soc. Psychol.* **44**, 113 (1983).

35. Jolliffe, I. *Principal component analysis*. (Wiley Online Library, 2002).

36. Viechtbauer, W. Conducting meta-analyses in R with the metafor package. *J. Stat. Softw.* **36**, 1–48 (2010).

37. Grossmann, I., Brienza, J. P. & Bobocel, D. R. Wise deliberation sustains cooperation. *Nat. Hum. Behav.* **1**, (2017).

38. Baltes, P. B. & Staudinger, U. M. Wisdom: a metaheuristic (pragmatic) to orchestrate mind and virtue toward excellence. *Am. Psychol.* **55**, 122 (2000).

39. Staudinger, U. M. & Glück, J. Psychological wisdom research: Commonalities and differences in a growing field. *Annu. Rev. Psychol.* **62**, 215–241 (2011).

40. Press Association. Race hate crime on UK railways soared after Brexit vote, figures show. *The Guardian* (2016).

41. Stone, J. Brexit: Surge in anti-immigrant hate crime in areas that voted to leave EU. *The Independent* (2016).

42. Brownstein, R. Why Trump voters need the immigrants they want to turn away. *CNN* (2018).

43. Meleady, R., Seger, C. R. & Vermue, M. Examining the role of positive and negative intergroup contact and anti-immigrant prejudice in Brexit. *Br. J. Soc. Psychol.* **56**, 799–808 (2017).

44. Hill, A. At least 1,000 highly skilled migrants wrongly face deportation, experts reveal | UK news. *The Guardian* (2018).

45. Gentleman, A. Windrush scandal: no passport for thousands who moved to Britain. *The Guardian* (2018).

46. Hayes, A. F. PROCESS SPSS Macro [Computer software and manual]. (2013). doi:10.1093/JOC

47. Pomfret, J. The coronavirus reawakens old racist tropes against Chinese people. *The Washington Post* (2020).

48. Peng, S. Smashed windows and racist graffiti: Vandals target Asian Americans amid coronavirus. *ABC News* (2020).

49. Bradsher, K. China Hails Its Virus Triumphs, and Glosses Over Its Mistakes. *The New York Times* (2020). Available at: https://www.nytimes.com/2020/06/07/world/asia/china-coronavirus.html. (Accessed: 1st July 2020)

50. Pennebaker, J. W., Booth, R. J. & Francis, M. E. Linguistic inquiry and word count: LIWC [Computer software]. *Austin, TX liwc. net* (2007).

51. Tausczik, Y. R. & Pennebaker, J. W. The Psychological Meaning of Words: LIWC and Computerized Text Analysis Methods. *J. Lang. Soc. Psychol.* **29**, 24–54 (2010).

52. Brewer, M. B. & Brown, R. J. Intergroup relations. in *The handbook of social psychology* (eds. Gilbert, D. T., Fiske, S. T. & Lindzey, G.) 554–594 (McGraw-Hill, 1998).

53. Fiske, S. T. What we know now about bias and intergroup conflict, the problem of the century. *Curr. Dir. Psychol. Sci.* **11**, 123–128 (2002).

54. Kung, F. Y. H. *et al.* Bridging racial divides: Social constructionist (vs. essentialist) beliefs facilitate trust in intergroup contexts. *J. Exp. Soc. Psychol.* **74**, 121–134 (2018).

55. Miller, D. T. & Prentice, D. A. Some consequences of a belief in group essence: The category divide hypothesis. in *Cultural divides: Understanding and overcoming group conflict* (eds. Prentice, D. A. & Miller, D.) 213–238 (Russell Sage Foundation, 1999).

1. We conducted additional post-hoc tests to examine the short version of the wise reasoning scale more closely. First for a larger sample, we used Study 3 responses to compare the psychometric properties of the full-length vs. short wise reasoning scale via confirmatory factor analysis (CFA). Study 3 responses were the closest comparison because participants from this study came from the same population as Study 1 participants—students at an English-speaking university in Hong Kong. We used the lavaan package (0.6.6) in R to conduct the tests. We found that compared to the full scale (AIC = 11891.47, CFI = .80, TLI = .78), the short version of the scale (AIC = 6821.27, CFI = .87, TLI = .84) demonstrated significantly better fit (i.e., AIC differences, *p* < .001). We then returned to Study 1 responses to test whether responses shortened scale demonstrated comparable properties, and we found similarly acceptable fit (CFI = .80, TLI = .76). [↑](#footnote-ref-1)
2. We asked participants to report their political view on social policy issues on a scale from 1 = *Very liberal* to 7 = *Very conservative*. Those with a score between 1 to 3 belong to the liberal group and those with a score between 5 to 7 belong to the conservative group. One hundred and twelve self-identified as Christian (e.g., Catholic, Protestant; 37%) and 139 were non-religious (59%); 49 reported to have non-heterosexual interests (e.g., homosexual, bisexual; 16%); 181 endorsed liberal (59%) and 71 endorsed conservative (23%) socio-political orientation. [↑](#footnote-ref-2)
3. Alternatively, we could compare only Christian heterosexual participants (*n* = 113) with non-Christian LGBTQ participants (*n* = 40). However, doing so would reduce the sample size almost by half (and reduce statistical power). Therefore, to retain as many participants as possible, we reported results using the current categorization of group membership. In the next study, we specifically recruited Christian heterosexual and sexual minority participants for a replication and extension. [↑](#footnote-ref-3)
4. Religious (vs. non-religious) LGB participants showed less negativity Christians; when excluded in analyses, the overall patterns of results did not change. [↑](#footnote-ref-4)
5. This was because positivity toward immigrants and fellow home citizens *both* predicted subscribing to volunteer. It is possible that people are aware of both self- and other-benefits to positive intergroup interactions, and that such interactions may therefore be variously motivated and not as dependent on polarization *per se*. Wise reasoning scores correlated with subscribing to volunteer, *r*=.117, *p*=.001, independent of condition, nationality, and political orientation (when controlling for these variables, *r*=.129, *p*<.001). [↑](#footnote-ref-5)
