# Supplementary material for: Wise reasoning, intergroup positivity, and attitude polarization across contexts
Source: Nat Commun. 2021 Jun 3;12:3313. doi: 10.1038/s41467-021-23432-1 (PMC8175723; doi:10.1038/s41467-021-23432-1)
Supplement: Supplementary file 2 — Reporting Summary [file 41467_2021_23432_MOESM2_ESM.pdf]

nature research

Corresponding author(s): Brienza, J. B., & Kung, F. Y. H.

Last updated by author(s): Oct 20, 2020

Reporting Summary

Nature Research wishes to improve the reproducibility of the work that we publish. This form provides structure for consistency and transparency in reporting. For further information on Nature Research policies, see Authors & Referees and the Editorial Policy Checklist.

Statistics

For all statistical analyses, confirm that the following items are present in the figure legend, table legend, main text, or Methods section.

n/a	Confirmed	
☐	☒	The exact sample size (*n*) for each experimental group/condition, given as a discrete number and unit of measurement
☐	☒	A statement on whether measurements were taken from distinct samples or whether the same sample was measured repeatedly
☐	☒	The statistical test(s) used AND whether they are one- or two-sided<br>*Only common tests should be described solely by name; describe more complex techniques in the Methods section.*
☐	☒	A description of all covariates tested
☐	☒	A description of any assumptions or corrections, such as tests of normality and adjustment for multiple comparisons
☐	☒	A full description of the statistical parameters including central tendency (e.g. means) or other basic estimates (e.g. regression coefficient) AND variation (e.g. standard deviation) or associated estimates of uncertainty (e.g. confidence intervals)
☐	☒	For null hypothesis testing, the test statistic (e.g. *F*, *t*, *r*) with confidence intervals, effect sizes, degrees of freedom and *P* value noted<br>*Give P values as exact values whenever suitable.*
☒	☐	For Bayesian analysis, information on the choice of priors and Markov chain Monte Carlo settings
☒	☐	For hierarchical and complex designs, identification of the appropriate level for tests and full reporting of outcomes
☐	☒	Estimates of effect sizes (e.g. Cohen's *d*, Pearson's *r*), indicating how they were calculated

Our web collection on statistics for biologists contains articles on many of the points above.

Software and code

Policy information about availability of computer code

Data collection	Data were collected through SONA university subject pools (Studies 1, 3, and 5), Mechanical Turk (Studies 2, 4, and 6) and Prolific (Studies 6 and 7). All surveys were administered on Qualtrics.
Data analysis	IMB SPSS Statistics v.22 (Studies 1 to 6) and v.24 (Study 7) was used for inferential analysis. Linguistic Inquiry and Word Count (LIWC) software (2015) was used for text analysis in Study 7.

For manuscripts utilizing custom algorithms or software that are central to the research but not yet described in published literature, software must be made available to editors/reviewers. We strongly encourage code deposition in a community repository (e.g. GitHub). See the Nature Research guidelines for submitting code & software for further information.

Data

Policy information about availability of data

All manuscripts must include a data availability statement. This statement should provide the following information, where applicable:

- Accession codes, unique identifiers, or web links for publicly available datasets
- A list of figures that have associated raw data
- A description of any restrictions on data availability

All data and syntax supporting the findings of this article are provided at https://osf.io/r247w/

Field-specific reporting

Please select the one below that is the best fit for your research. If you are not sure, read the appropriate sections before making your selection.

☐ Life sciences ☒ Behavioural & social sciences ☐ Ecological, evolutionary & environmental sciences

Behavioural & social sciences study design

All studies must disclose on these points even when the disclosure is negative.

Study description	Studies 1 to 5 were quantitative and correlational; Study 6 was quantitative and experimental.
Research sample	Study 1: Chinese undergraduate students in Hong Kong;<br>Study 2: US residents who were English speaking adults;<br>Study 3: Chinese undergraduate students in Hong Kong;<br>Study 4: US residents who were English speaking adults. They were either self-identified as Christian and/or endorsed conservative sociopolitical orientation or they were non-religious, regardless of sexual orientation, who endorsed liberal sociopolitical orientation;<br>Study 5: Christian heterosexual and Lesbian/Gay/Bisexual undergraduate students in Canada;<br>Study 6: White adults who were residents in the US and UK<br>Study 7: White adults who were US citizens or lawful permanent residents living in the US<br>Additional key demographics of all samples are summarized in Table S1 in the online Supporting Information. The studies recruited only adult samples; their mean age ranged from 19.1 to 39.42, so the results may not generalize to younger and older populations.
Sampling strategy	Studies 1, 3, and 5 used convenience sample from university subject pools; Studies 2, 4, 6 and 7 involved random sample on Mturk and Prolific. The sensitivity power analysis of each study is reported in Supporting information, indicating the minimum effect size the study affords the focal analysis (with the given sample size, with 80% power, and at the conventional significance level of .05; Faul, Erdfelder, Buchner, & Lang, 2009). As past research suggested, these sources of data overall are representative of the populations; the samples are reasonably representative of the general population across most psychological dimensions such as political orientation (Clifford, Jewell, Waggoner, 2015; McCredie & Morey, 2018).
Data collection	All responses from participants were collected via Qualtrics. In Studies 6 and 7, because the randomization was conducted on Qualtrics automatically, researchers were blind to the condition participants were in during the experiment.
Timing	Study 1: November 2014, Hong Kong; Study 2: April 2015, USA; Study 3: September-December, 2015, Hong Kong; Study 4: June, 2015, USA; Study 5: October 2014-February 2015, Canada; Study 6: March, 2018, USA and UK; Study 7: June, 2020, USA.
Data exclusions	Data exclusion, if any, in each study was reported in the "Participants, Procedure, and Power" section in Supporting Information.<br>Study 1: One participant had missing data on key measures and was excluded from analyses, resulting in a final sample size of 74.<br>Study 2: Nine participants had missing data on key measures and were not included in the analysis, resulting in a final sample of 337 participants.<br>Study 3: No missing data and exclusion.<br>Study 4: 306 have participated. Within this sample, we selected participants that could represent either side of the conflict on same-sex marriage in USA (as a function of religiosity and political conservatism). For one group, we included heterosexual participants who self-identified as Christian and/or endorsed conservative sociopolitical orientation (n = 144). For the other group, we included non-religious participants, regardless of sexual orientation, who endorsed liberal sociopolitical orientation (n = 99). This resulted in a final sample of 243 in the analysis.<br>Study 5: No missing data and exclusion.<br>Study 6: 932 were recruited to participate in the survey, but for the purpose of the experiment, we focus only on White majority participants' intergroup bias (fellow citizen and immigrant minority target groups), resulting in a final total sample of 776.<br>Study 7: 903 were recruited; after pre-registered demographic and data quality exclusion criteria, the final sample was 791.<br><br>In each study, participants voluntarily signed up to participate in the study. They were either self-identified or were pre-selected based on demographics as part of a group that was involved in the intergroup conflict. They reflected on stimulus materials related to a recent and self-relevant conflict (e.g., news clippings of the conflicts). They then indicated their attitudes to groups in the conflict as a measure of intergroup bias (warmth and trust or feeling thermometer ratings) and responded to the wise reasoning measure assessing their reflections on the conflict. In the experiment, we used a brief online wise reasoning reflection exercise (described in full in the supplement). Participants' demographics (e.g., age, gender, education, and race) were collected at the end of the study. In all studies, the researchers were not blind to the hypotheses, however, the studies were conducted online, not in the presence of the researchers.
Non-participation	The studies yielded no reliable estimates of participation rate, due to the nature of the data collection method that there was no documentation of the number of participants who have signed up but did not begin or complete the survey. Less than 2.6% participants dropped out in Studies 1 to 5. Study 6 (which used a writing task) yielded a higher attrition rate of 14% (USA) and 10% (UK) respectively. When possible, we conducted attrition analyses (using univariate tests of variance) which indicated no significant differences between the participants who completed the survey and participants who dropped out (p's > .095), with two exceptions which are noted in Study 5 in the online Supporting Information.
Randomization	Random assignment of participants into experimental vs. control condition was executed through the randomization function in survey flow on Qualtrics.

Reporting for specific materials, systems and methods

We require information from authors about some types of materials, experimental systems and methods used in many studies. Here, indicate whether each material, system or method listed is relevant to your study. If you are not sure if a list item applies to your research, read the appropriate section before selecting a response.

Materials & experimental systems

n/a	Involved in the study
☒	☐ Antibodies
☒	☐ Eukaryotic cell lines
☒	☐ Palaeontology
☒	☐ Animals and other organisms
☐	☒ Human research participants
☒	☐ Clinical data

Methods

n/a	Involved in the study
☒	☐ ChIP-seq
☒	☐ Flow cytometry
☒	☐ MRI-based neuroimaging

Human research participants

Policy information about studies involving human research participants

Population characteristics	See above.
Recruitment	Participants were recruited through SONA university subject pools (Studies 1, 3, and 5), Mechanical Turk (Studies 2, 4, and 6) and Prolific (Studies 6 and 7). All studies were advertised to participants as a survey concerning their opinions about social issues in general to minimize self-selection bias. People who do not have opinions about social issues may self-select not to participate in the study, though it is unclear whether this is an issue and to what extent it may impact the results.
Ethics oversight	The studies were carried out in accordance with the recommendations of the Human Participants Research Panel of the Hong Kong University of Science and Technology, the Human Research Ethics Committee at the University of Waterloo, the Office for Human Research Protections at Purdue University, and the University of Queensland and the National Statement on Ethical Conduct in Human Research of Australia.

Note that full information on the approval of the study protocol must also be provided in the manuscript.
